# Supplementary material for: Oxidative 1,2-carboamination of alkenes with alkyl nitriles and amines toward γ-amino alkyl nitriles
Source: Nat Commun. 2017 Apr 10;8:14720. doi: 10.1038/ncomms14720 (PMC5394231; doi:10.1038/ncomms14720)
Supplement: Supplementary Information — Supplementary figures, supplementary tables, supplementary methods, and supplementary references. [file ncomms14720-s1.pdf]

Supplementary Methods

1. Materials

Substrates **1i**[1], **1m**[2], **1n**[3], **1p**[4], **1r**[5], **1s**[6], and **1t**[7] were prepared according to literature procedures. Other alkenes, amines and nitriles were purchased from commercial sources and were used as received.

2. General Procedures for Silver-Mediated Intermolecular 1,2-Carboamination of Alkenes with Nitriles and Amines:

To a Schlenk tube were added $Fe(OTf)_3$ or $FeCl_3$ (10 mol%), Ag_2CO_3 (0.6 mmol), alkene **1** (0.3 mmol), amine **3** (0.6mmol) and MeCN (2 mL). Then the tube was recharged with argon, and the mixture was stirred at 120 ºC for 24 hours. After cooling to room temperature, the mixture was filtered through a small plug of silica gel to remove the precipitate and washed with with EtOAc (3×10 mL). The solvent was then removed in vacuo and the residue was further purifiedby silica gel flash column chromatography (10-40% ethyl acetate/hexane + 0.1% Et_3N) to afford the desired product.

3. Analytical data for 4-15, 17-26, 28-29 and 31-44

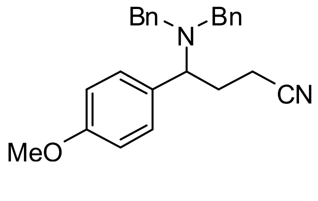

4-(Dibenzylamino)-4-(4-methoxyphenyl)butanenitrile (4): Light yellow solid; mp 140.3-140.9 ºC (uncorrected); ^{1}H NMR (400 MHz, $CDCl_3$) δ: 7.36-7.31 (m, 8H), 7.26-7.22 (m, 2H), 7.13 (d, *J* = 8.0 Hz, 2H), 6.94 (d, *J* = 8.0 Hz, 2H), 3.84 (s, 3H), 3.79 (d, *J* = 13.6 Hz, 2H), 3.69 (t, *J* = 7.6 Hz, 1H), 3.10 (d, *J* = 14.0 Hz, 2H), 2.54-2.47 (m, 1H), 2.42-2.24 (m, 2H), 2.03-1.94 (m, 1H); ^{13}C NMR (100 MHz,

$CDCl_3$) δ: 159.0, 139.5, 129.8, 128.7, 128.6, 128.4, 127.1, 119.8, 113.6, 60.4, 55.2, 53.7, 28.1, 14.8; LRMS (EI, 70 eV) *m/z* (%): 370 (M^+, 2), 316 (93), 174 (19), 134 (22), 91 (100); HRMS *m/z* (ESI) calcd for $C_{25}H_{27}N_2O$ $(M+H)^+$ 371.2118, found 371.2124.

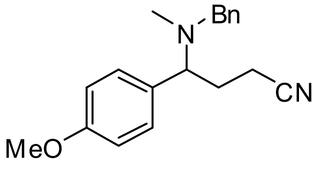

4-(Benzyl(methyl)amino)-4-(4-methoxyphenyl)butanenitrile (5): Light yellow oil; ^1H NMR (400 MHz, $CDCl_3$) δ: 7.33-7.22 (m, 5H), 7.16 (d, *J* = 8.0 Hz, 2H), 6.91 (d, *J* = 8.4 Hz, 2H), 3.81 (s, 3H), 3.63 (t, *J* = 7.2 Hz, 1H), 3.50 (d, *J* = 13.6 Hz, 1H), 3.10 (d, *J* = 13.2 Hz, 1H), 2.44-2.29 (m, 3H), 2.07 (s, 3H), 2.01-1.93 (m, 1H); ^{13}C NMR (100 MHz, $CDCl_3$) δ: 159.0, 139.3, 129.5, 129.3, 128.6, 128.2, 126.9, 119.9, 113.6, 65.4, 58.7, 55.2, 37.2, 28.3, 14.6; LRMS (EI, 70 eV) *m/z* (%): 294 (M^+, 2), 240 (98), 174 (17), 134 (24), 91 (100); HRMS *m/z* (ESI) calcd for $C_{19}H_{23}N_2O$ $(M+H)^+$ 295.1805, found 295.1813.

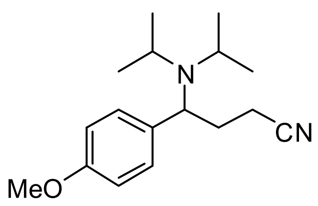

4-(Diisopropylamino)-4-(4-methoxyphenyl)butanenitrile (6): Colorless oil; ^1H NMR (400 MHz, $CDCl_3$) δ: 7.23 (d, *J* = 8.4 Hz, 2H), 6.83 (d, *J* = 8.4 Hz, 2H), 3.89 (t, *J* = 7.6 Hz, 1H), 3.80 (s, 3H), 3.27-3.17 (m, 2H), 2.37-2.22 (m, 2H), 2.19-2.09 (m, 1H), 2.05-1.96 (m, 1H), 1.09 (d, *J* = 6.8 Hz, 6H), 0.85 (d, *J* = 6.4 Hz, 6H); ^{13}C NMR (100 MHz, $CDCl_3$) δ: 158.4, 134.6, 129.3, 120.2, 113.5, 56.6, 55.2, 45.1, 30.4, 23.8, 22.1, 15.1; LRMS (EI, 70 eV) *m/z* (%): 274 (M^+, 6), 259 (4), 220 (53), 174 (100), 134 (38); HRMS *m/z* (ESI) calcd for $C_{17}H_{27}N_2O$ $(M+H)^+$ 275.2118, found 275.2126.

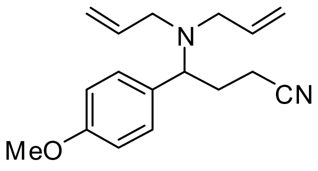

4-(Diallylamino)-4-(4-methoxyphenyl)butanenitrile (7): Colorless oil; ^{1}H NMR (400 MHz, $CDCl_3$) δ: 7.11 (d, *J* = 8.0 Hz, 2H), 6.88 (d, *J* = 8.0 Hz, 2H), 5.86-5.76 (m, 2H), 5.20-5.14 (m, 4H), 3.84-3.81 (m, 4H), 3.25 (dd, *J* = 2.8, 14.4 Hz, 2H), 2.68 (dd, *J* = 7.6, 14.4 Hz, 2H), 2.47-2.37 (m, 2H), 2.35-2.24 (m, 1H), 1.98-1.89 (m, 1H); ^{13}C NMR (100 MHz, $CDCl_3$) δ: 158.9, 136.5, 129.5, 119.9, 117.2, 113.6, 60.8, 55.2, 52.5, 28.3, 14.7; LRMS (EI, 70 eV) *m/z* (%): 270 (M^+, 2), 216 (100), 174 (29), 134 (35), 121 (9); HRMS *m/z* (ESI) calcd for $C_{17}H_{23}N_2O$ $(M+H)^+$ 271.1805, found 271.1800.

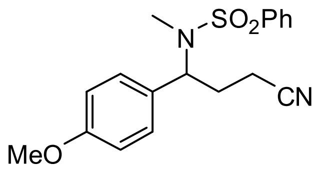

***N*-(3-Cyano-1-(4-methoxyphenyl)propyl)-*N*-methylbenzenesulfonamide (8):** Light yellow oil; ^{1}H NMR (400 MHz, $CDCl_3$) δ: 7.78 (d, *J* = 8.0 Hz, 2H), 7.60-7.48 (m, 3H), 6.92 (d, *J* = 8.4 Hz, 2H), 6.74 (d, *J* = 8.4 Hz, 2H), 5.04 (t, *J* = 8.0 Hz, 1H), 3.76 (s, 3H), 2.61 (s, 3H), 2.42 (t, *J* = 7.2 Hz, 2H), 2.19 (q, *J* = 7.2 Hz, 2H); ^{13}C NMR (100 MHz, $CDCl_3$) δ: 159.4, 139.7, 132.6, 129.1, 129.0, 127.8, 127.0, 119.0, 113.9, 58.6, 55.2, 29.0, 27.1, 14.8; LRMS (EI, 70 eV) *m/z* (%): 344 (M^+, 2), 290 (100), 203 (48), 174 (98), 148 (77), 134 (64); HRMS *m/z* (ESI) calcd for $C_{18}H_{21}N_2O_3S$ $(M+H)^+$ 345.1267, found 345.1277.

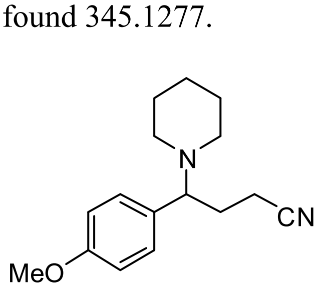

4-(4-Methoxyphenyl)-4-(piperidin-1-yl)butanenitrile (9): Colorless oil; ^{1}H NMR (400 MHz, $CDCl_3$) δ: 7.09 (d, *J* = 8.4 Hz, 2H), 6.87 (d, *J* = 8.4 Hz, 2H), 3.81 (s, 3H), 3.44 (t, *J* = 7.2 Hz, 1H), 2.41-2.24 (m, 7H), 1.99-1.91 (m, 1H), 1.58-1.47 (m, 4H), 1.37-1.30 (m, 2H); ^{13}C NMR (100 MHz, $CDCl_3$) δ: 158.9, 129.7, 129.5, 120.0, 113.5, 67.7, 55.2,

50.8, 28.0, 26.3, 24.5, 14.5; LRMS (EI, 70 eV) *m/z* (%): 258 (M^+, 2), 204 (100), 174(10), 134 (19)1 121 (11); HRMS *m/z* (ESI) calcd for $C_{16}H_{23}N_2O$ $(M+H)^+$ 259.1805, found 259.1816.

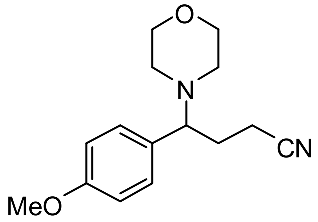

4-(4-Methoxyphenyl)-4-morpholinobutanenitrile (10): Colorless oil; ^1H NMR (400 MHz, $CDCl_3$) δ: 7.13 (d, *J* = 8.0 Hz, 2H), 6.88 (d, *J* = 8.4 Hz, 2H), 3.81 (s, 3H), 3.67 (t, *J* = 4.4 Hz, 4H), 3.37 (t, *J* = 7.2 Hz, 1H), 2.39 (brs, 4H), 2.36-2.25 (m, 2H), 2.21-2.13 (m, 1H), 1.99-1.89 (m, 1H); ^{13}C NMR (100 MHz, $CDCl_3$) δ: 159.2, 129.6, 129.4, 119.6, 113.8, 67.8, 67.0, 55.2, 50.6, 27.9, 14.3; LRMS (EI, 70 eV) *m/z* (%): 260 (M^+, 3), 206 (100), 174 (24), 134 (28), 119 (6); HRMS *m/z* (ESI) calcd for $C_{15}H_{21}N_2O_2$ $(M+H)^+$ 261.1598, found 261.1612.

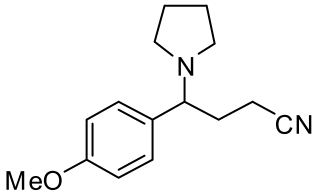

4-(4-Methoxyphenyl)-4-(pyrrolidin-1-yl)butanenitrile (11): Colorless oil; ^1H NMR (400 MHz, $CDCl_3$) δ: 7.21 (d, *J* = 8.0 Hz, 2H), 6.87 (d, *J* = 8.4 Hz, 2H), 3.81 (s, 3H), 3.20 (t, *J* = 4.8 Hz, 1H), 2.55-2.50 (m, 2H), 2.39-2.36 (m, 2H), 2.29-2.17 (m, 2H), 2.04-1.95 (m, 2H), 1.79-1.69 (m, 4H); ^{13}C NMR (100 MHz, $CDCl_3$) δ: 159.0, 132.6, 128.9, 119.7, 113.9, 68.2, 55.2, 52.4, 31.2, 23.2, 13.9; LRMS (EI, 70 eV) *m/z* (%): 244 (M^+, 1), 190 (100), 174 (7), 134 (14), 121 (7); HRMS *m/z* (ESI) calcd for $C_{15}H_{21}N_2O$ $(M+H)^+$ 245.1648, found 245.1661.

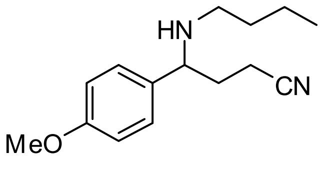

4-(Butylamino)-4-(4-methoxyphenyl)butanenitrile (12): Light yellow oil; ^1H NMR (400 MHz, $CDCl_3$) δ: 7.19 (d, *J* = 8.0 Hz, 2H), 6.89 (d, *J* = 8.4 Hz, 2H), 3.81 (s, 3H), 3.65 (t, *J* = 6.4 Hz, 1H),

2.47-2.41 (m, 2H), 2.39-2.31 (m, 1H), 2.20-2.12 (m, 1H), 2.07-1.98 (m, 1H), 1.93-1.84 (m, 1H), 1.48-1.25 (m, 5H), 0.87 (t, J = 7.2 Hz, 3H); ^{13}C NMR (100 MHz, $CDCl_3$) δ: 159.0, 134.2, 128.0, 119.8, 114.1, 61.2, 55.3, 47.2, 33.2, 32.3, 20.4, 14.1, 14.0; LRMS (EI, 70 eV) *m/z* (%): 246 (M^+, 1), 192 (100), 174 (26), 134 (22), 121 (6); HRMS *m/z* (ESI) calcd for $C_{15}H_{23}N_2O$ $(M+H)^+$ 247.1805, found 247.1817.

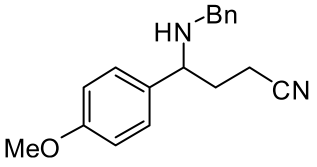

4-(Benzylamino)-4-(4-methoxyphenyl)butanenitrile (13): Light yellow oil; ^{1}H NMR (400 MHz, $CDCl_3$) δ: 7.33-7.30 (m, 7H), 6.91 (d, J = 8.0 Hz, 2H), 3.82 (s, 3H), 3.70-3.63 (m, 2H), 3.55 (d, J = 13.2 Hz, 1H), 2.39-2.17 (m, 2H), 2.06-1.87 (m, 2H), 1.57 (s, 1H); ^{13}C NMR (100 MHz, $CDCl_3$) δ: 159.1, 140.1, 133.7, 128.4, 128.1, 128.0, 127.0, 119.7, 114.1, 60.3, 55.2, 51.3, 33.3, 14.1; LRMS (EI, 70 eV) *m/z* (%): 280 (M^+, 1), 226 (83), 174 (5), 134 (14), 91 (100); HRMS *m/z* (ESI) calcd for $C_{18}H_{21}N_2O$ $(M+H)^+$ 281.1648, found 281.1641.

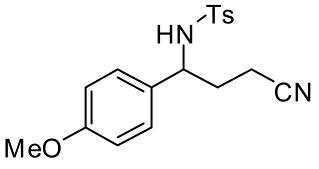

***N*-(3-Cyano-1-(4-methoxyphenyl)propyl)-4-methylbenzenesulfonamide (14):** Colorless oil; ^{1}H NMR (400 MHz, $CDCl_3$) δ: 7.57 (d, J = 7.6 Hz, 2H), 7.16 (d, J = 8.0 Hz, 2H), 6.89 (d, J = 8.4 Hz, 2H), 6.69 (d, J = 8.4 Hz, 2H), 5.49 (d, J = 7.2 Hz, 1H), 4.24 (q, J = 7.6 Hz, 1H), 3.74 (s, 3H), 2.38 (s, 3H), 2.42-2.25 (m, 2H), 2.23-2.13 (m, 1H), 2.05-1.94 (m, 1H); ^{13}C NMR (100 MHz, $CDCl_3$) δ: 159.3, 143.4, 137.0, 130.7, 129.5, 127.5, 127.0, 118.9, 114.2, 56.6, 55.2, 32.8, 21.4, 14.3; LRMS (EI, 70 eV) *m/z* (%): 344 (M^+, 3), 290 (100), 189 (36), 155 (59), 134 (45); HRMS *m/z* (ESI) calcd for $C_{18}H_{21}N_2O_3S$ $(M+H)^+$ 345.1267, found 345.1259.

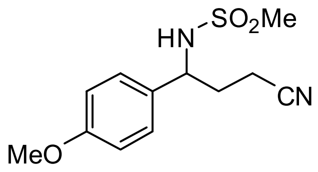

***N*-(3-Cyano-1-(4-methoxyphenyl)propyl)methanesulfonamide (15):** Colorless oil; ^{1}H NMR (400 MHz, $CDCl_3$) δ: 7.24 (d, *J* = 8.0 Hz, 2H), 6.93 (d, *J* = 8.0 Hz, 2H), 5.28 (d, *J* = 8.0 Hz, 1H), 4.46 (q, *J* = 8.0 Hz, 1H), 3.82 (s, 3H), 2.63 (s, 3H), 2.47-2.32 (m, 2H), 2.26-2.17 (m, 1H), 2.10-2.01 (m, 1H); ^{13}C NMR (100 MHz, $CDCl_3$) δ: 159.8, 131.2, 127.6, 118.9, 114.7, 56.8, 55.3, 41.7, 33.0, 14.5; LRMS (EI, 70 eV) *m/z* (%): 268 (M^+, 10), 214 (100), 187 (16), 134 (61), 108 (24); HRMS *m/z* (ESI) calcd for $C_{12}H_{17}N_2O_3S$ $(M+H)^+$ 269.0954, found 269.0943.

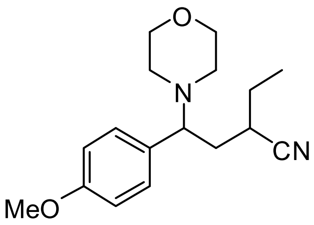

2-Ethyl-4-(4-methoxyphenyl)-4-morpholinobutanenitrile (17): Colorless oil; d.r. = 1.3:1; ^{1}H NMR (400 MHz, $CDCl_3$) δ: 7.18 (d, *J* = 7.6 Hz, 2H), 7.09 (d, *J* = 7.6 Hz, 2H), 6.90-6.87 (m, 4H), 3.81 (s, 6H), 3.67-3.61 (m, 9H), 3.48-3.42 (m, 1H), 2.81-2.73 (m, 1H), 2.42-2.06 (m, 11H), 1.90-1.74 (m, 2H), 1.71-1.55 (m, 4H), 1.09 (t, *J* = 7.2 Hz, 3H), 1.01 (t, *J* = 7.2 Hz, 3H); ^{13}C NMR (100 MHz, $CDCl_3$) δ: 159.2, 159.1, 130.2, 129.6, 129.4, 128.7, 122.2, 121.9, 113.9, 113.5, 67.6, 67.2, 67.0, 66.1, 55.2, 51.0, 49.7, 35.0, 33.7, 30.7, 30.4, 25.9, 25.3, 11.5, 11.4; LRMS (EI, 70 eV) *m/z* (%): 288 (M^+, 2), 206 (100), 134 (26), 121 (12); HRMS *m/z* (ESI) calcd for $C_{17}H_{25}N_2O_2$ $(M+H)^+$ 289.1911, found 289.1925.

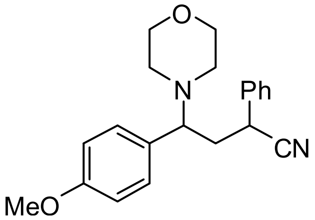

4-(4-Methoxyphenyl)-4-morpholino-2-phenylbutanenitrile (18): Colorless oil; d.r. = 1:1; ^{1}H NMR (400 MHz, $CDCl_3$) δ: 7.41-7.26 (m, 10H), 7.17 (d, *J* = 8.0 Hz, 2H), 7.05 (d, *J* = 8.4 Hz, 2H), 6.92-6.87 (m, 4H), 4.04 (t, *J* = 7.6 Hz, 1H), 3.82 (s, 6H),

3.73-3.55 (m, 10H), 3.44 (t, *J* = 8.0 Hz, 1H), 2.67-2.19 (m, 11H), 2.09-2.02 (m, 1H); ^{13}C NMR (100 MHz, $CDCl_3$) δ: 159.3, 159.2, 135.9, 135.8, 129.8, 129.6, 129.4, 129.1 (2C), 128.1, 128.0, 127.4, 127.3, 121.1, 120.9, 113.9, 113.6, 67.2, 67.1, 66.5, 65.8, 55.3, 50.5, 49.6, 38.8, 37.6, 34.6; LRMS (EI, 70 eV) *m/z* (%): 336 (M^+, 2), 249 (5), 206 (100), 134 (29), 116 (8); HRMS *m/z* (ESI) calcd for $C_{21}H_{25}N_2O_2$ $(M+H)^+$ 337.1911, found 337.1923.

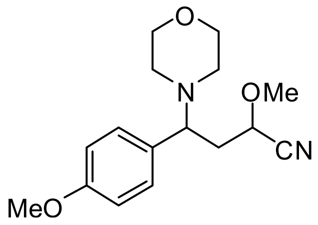

2-Methoxy-4-(4-methoxyphenyl)-4-morpholinobutanenitrile (19): Colorless oil; d.r. = 1.2:1; ^{1}H NMR (400 MHz, $CDCl_3$) δ: 7.12 (d, *J* = 8.4 Hz, 4H), 6.89 (d, *J* = 8.0 Hz, 4H), 4.04 (t, *J* = 7.2 Hz, 1H), 3.83-3.80 (m, 7H), 3.67-3.53 (m, 10H), 3.44 (s, 3H), 3.39 (s, 3H), 2.62-2.34 (m, 10H), 2.14-1.96 (m, 2H); ^{13}C NMR (100 MHz, $CDCl_3$) δ: 159.2, 129.6, 129.5, 129.2, 128.9, 118.4, 118.2, 113.8, 113.7, 68.4, 67.1 (2C), 65.0, 64.4, 58.0, 57.9, 55.2, 50.6, 50.1, 36.1, 35.8; LRMS (EI, 70 eV) *m/z* (%): 290 (M^+, 4), 206 (100), 172 (4), 134 (37), 121 (9); HRMS *m/z* (ESI) calcd for $C_{16}H_{23}N_2O_3$ $(M+H)^+$ 291.1703, found 291.1712.

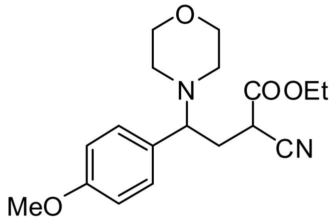

Ethyl 2-cyano-4-(4-methoxyphenyl)-4-morpholinobutanoate (20): Colorless oil; d.r. = 1.1:1; ^{1}H NMR (400 MHz, $CDCl_3$) δ: 7.15 (d, *J* = 8.0 Hz, 2H), 7.09 (d, *J* = 8.4 Hz, 2H), 6.91-6.88 (m, 4H), 4.30-4.22 (m, 4H), 3.93 (dd, *J* = 5.2, 9.2 Hz, 1H), 3.82 (s, 6H), 3.69-3.60 (m, 10H), 3.38 (t, *J* = 6.8 Hz, 1H), 2.77-2.64 (m, 2H), 2.48-2.31 (m, 8H), 2.20-2.05 (m, 2H), 1.33 (t, *J* = 7.2 Hz, 6H); ^{13}C NMR (100 MHz, $CDCl_3$) δ: 166.5, 166.1, 159.3 (2C), 129.7, 129.6, 128.3,

127.6, 116.7, 116.5, 113.9, 113.6, 67.1, 67.0, 66.5, 65.6, 62.8, 55.2, 50.3, 49.4, 35.5, 35.1, 31.6, 31.5, 14.0 (2C); LRMS (EI, 70 eV) *m/z* (%): 332 (M^+, 2), 206 (100), 173 (11), 134 (7); HRMS *m/z* (ESI) calcd for $C_{18}H_{25}N_2O_4$ $(M+H)^+$ 333.1809, found 333.1817.

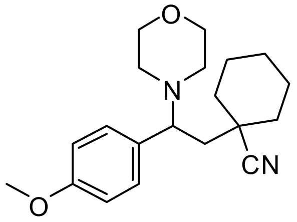

1-(2-(4-Methoxyphenyl)-2-morpholinoethyl)cyclohexanecarbonitrile (21): Colorless oil; ^{1}H NMR (400 MHz, $CDCl_3$) δ: 7.09 (d, *J* = 8.4 Hz, 2H), 6.87 (d, *J* = 8.4 Hz, 2H), 3.81 (s, 3H), 3.74 (t, *J* = 7.2 Hz, 1H), 3.68 (t, *J* = 4.8 Hz, 4H), 2.48-2.43 (m, 2H), 2.36-2.31 (m, 2H), 2.15 (d, *J* = 13.6 Hz, 1H), 1.81-1.53 (m, 7H), 1.35-1.26 (m, 2H), 1.18-1.08 (m, 2H); ^{13}C NMR (100 MHz, $CDCl_3$) δ: 159.0, 129.9, 129.3, 123.6, 113.4, 67.0, 65.5, 55.2, 49.9, 41.7, 37.9, 37.5, 35.4, 25.3, 22.9 (2C); LRMS (EI, 70 eV) *m/z* (%): 328 (M^+, 1), 242 (6), 206 (100), 135 (12), 121 (8); HRMS *m/z* (ESI) calcd for $C_{20}H_{29}N_2O_2$ $(M+H)^+$ 329.2224, found 329.2233.

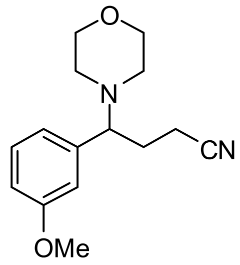

4-(3-Methoxyphenyl)-4-morpholinobutanenitrile (22): Colorless oil; ^{1}H NMR (400 MHz, $CDCl_3$) δ: 7.27 (t, *J* = 8.0 Hz, 1H), 6.85-6.76 (m, 3H), 3.82 (s, 3H), 3.68 (t, *J* = 4.0 Hz, 4H), 3.37 (t, *J* = 6.8 Hz, 1H), 2.41 (brs, 4H), 2.38-2.13 (m, 3H), 1.98-1.91 (m, 1H); ^{13}C NMR (100 MHz, $CDCl_3$) δ: 159.7, 139.5, 129.5, 120.7, 119.6, 114.3, 112.8, 68.5, 67.1, 55.2, 50.7, 27.9, 14.2; LRMS (EI, 70 eV) *m/z* (%): 260 (M^+, 2), 206 (100), 174 (5), 134 (13), 91 (7); HRMS *m/z* (ESI) calcd for $C_{15}H_{21}N_2O_2$ $(M+H)^+$ 261.1598, found 261.1609.

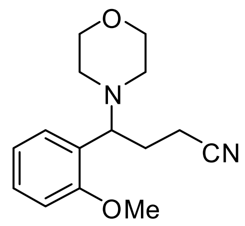

4-(2-Methoxyphenyl)-4-morpholinobutanenitrile (23): Colorless oil; ^{1}H NMR (400 MHz, $CDCl_3$) δ: 7.28-7.20 (m, 2H), 6.97-6.89 (m, 2H), 4.07 (t, *J* = 6.4 Hz, 1H), 3.81 (s, 3H), 3.67 (t, *J* = 4.4 Hz, 4H), 2.42 (brs, 4H), 2.34-2.22 (m, 3H), 2.00-1.92 (m, 1H); ^{13}C NMR (100 MHz, $CDCl_3$) δ: 158.9, 136.5, 129.5, 119.9, 117.2, 113.6, 60.8, 55.2, 52.5, 28.3, 14.7; LRMS (EI, 70 eV) *m/z* (%): 260 (M^+, 2), 206 (100), 174 (4), 147 (12), 91 (12); HRMS *m/z* (ESI) calcd for $C_{15}H_{21}N_2O_2$ $(M+H)^+$ 261.1598, found 261.1608.

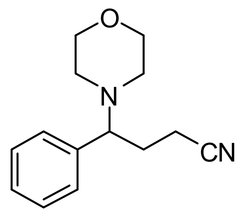

4-Morpholino-4-phenylbutanenitrile (24): Colorless oil; ^{1}H NMR (400 MHz, $CDCl_3$) δ: 7.37-7.29 (m, 3H), 7.21 (d, *J* = 7.2 Hz, 2H), 3.68 (t, *J* = 4.8 Hz, 4H), 3.42 (t, *J* = 7.2 Hz, 1H), 2.41 (brs, 4H), 2.37-2.27 (m, 2H), 2.23-2.14 (m, 1H), 2.03-1.93 (m, 1H); ^{13}C NMR (100 MHz, $CDCl_3$) δ: 137.7, 128.5, 128.4, 127.9, 119.6, 68.5, 67.1, 50.6, 27.8, 14.2; LRMS (EI, 70 eV) *m/z* (%): 230 (M^+, 2), 176 (100), 144 (6), 105 (9), 91 (16); HRMS *m/z* (ESI) calcd for $C_{14}H_{19}N_2O$ $(M+H)^+$ 231.1492, found 231.1504.

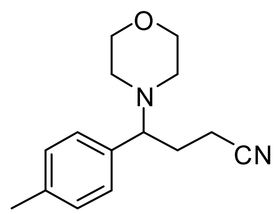

4-Morpholino-4-*p*-tolylbutanenitrile (25): Colorless oil; ^{1}H NMR (400 MHz, $CDCl_3$) δ: 7.16 (d, *J* = 7.2 Hz, 2H), 7.09 (d, *J* = 7.6 Hz, 2H), 3.67 (t, *J* = 4.8 Hz, 4H), 3.38 (t, *J* = 7.2 Hz, 1H), 2.39 (brs, 4H), 2.35 (s, 3H), 2.32-2.25 (m, 2H), 2.22-2.14 (m, 1H), 2.01-1.91 (m, 1H); ^{13}C NMR (100 MHz, $CDCl_3$) δ: 137.7, 134.6, 129.2, 128.3, 119.7, 68.2, 67.1, 50.6, 27.9, 21.1, 14.3; LRMS (EI, 70 eV) *m/z* (%): 244 (M^+, 2), 190 (100), 158 (8), 117 (10); HRMS *m/z* (ESI) calcd for $C_{15}H_{21}N_2O$ $(M+H)^+$ 245.1648, found 245.1653.

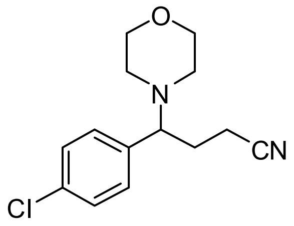

4-(4-Chlorophenyl)-4-morpholinobutanenitrile (26): Colorless oil; ^{1}H NMR (400 MHz, $CDCl_3$) δ: 7.34 (d, *J* = 8.0 Hz, 2H), 7.16 (d, *J* = 8.4 Hz, 2H), 3.67 (t, *J* = 4.4 Hz, 4H), 3.42 (t, *J* = 7.2 Hz, 1H), 2.39-2.25 (m, 6H), 2.22-2.15 (m, 1H), 1.97-1.89 (m, 1H); ^{13}C NMR (100 MHz, $CDCl_3$) δ: 136.2, 133.7, 129.6, 128.7, 119.3, 67.8, 67.0, 50.5, 27.8, 14.2; LRMS (EI, 70 eV) *m/z* (%): 266(M^{+}+2, 1), 264 (M^{+}, 3), 210 (100), 178 (8), 138 (9), 125 (15); HRMS *m/z* (ESI) calcd for $C_{14}H_{18}{}^{35}ClN_2O$ $(M+H)^{+}$ 265.1102, found 265.1111.

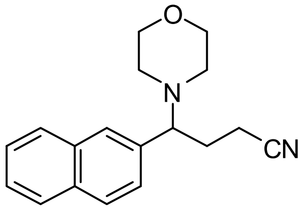

4-Morpholino-4-(naphthalen-2-yl)butanenitrile (28): Colorless oil; ^{1}H NMR (400 MHz, $CDCl_3$) δ: 7.86-7.82 (m, 3H), 7.67 (s, 1H), 7.53-7.48 (m, 2H), 7.38 (d, *J* = 8.4 Hz, 1H), 3.69 (t, *J* = 4.4 Hz, 4H), 3.56 (t, *J* = 7.2 Hz, 1H), 2.47-2.30 (m, 6H), 2.20-2.02 (m, 2H); ^{13}C NMR (100 MHz, $CDCl_3$) δ: 135.6, 133.1, 128.5, 127.8, 127.7, 127.6, 126.4, 126.2, 125.6, 119.5, 68.8, 67.1, 51.0, 28.0, 14.3; LRMS (EI, 70 eV) *m/z* (%): 280 (M^{+}, 4), 226 (100), 194 (6), 154 (23), 127 (7); HRMS *m/z* (ESI) calcd for $C_{18}H_{21}N_2O$ $(M+H)^{+}$ 281.1648, found 281.1654.

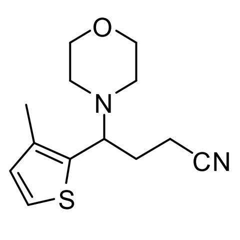

4-(3-Methylthiophen-2-yl)-4-morpholinobutanenitrile (29): Colorless oil; ^{1}H NMR (400 MHz, $CDCl_3$) δ: 7.20 (d, *J* = 4.8 Hz, 1H), 6.82 (d, *J* = 4.8 Hz, 1H), 3.89 (t, *J* = 7.2 Hz, 1H), 3.73-3.67 (m, 4H), 2.54-2.21 (m, 10H), 1.99-1.92 (m, 1H); ^{13}C NMR (100 MHz, $CDCl_3$) δ: 136.2, 134.9, 129.9, 123.8, 119.4, 67.0, 61.0, 50.5, 29.8, 14.4, 14.3; LRMS (EI, 70 eV)

m/z (%): 250 (M^+, 3), 196 (100), 164 (32), 124 (22); HRMS *m/z* (ESI) calcd for $C_{13}H_{19}N_2OS$ $(M+H)^+$ 251.1213, found 251.1221.

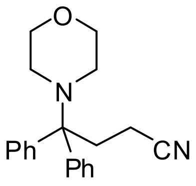

4-Morpholino-4,4-diphenylbutanenitrile (31): White solid; mp 164.1-164.8 ºC (uncorrected); ^{1}H NMR (400 MHz, $CDCl_3$) δ: 7.38-7.26 (m, 10H), 3.75 (t, *J* = 4.8 Hz, 4H), 2.56 (t, *J* = 7.6 Hz, 2H), 2.30 (brs, 4H), 2.06 (t, *J* = 8.0 Hz, 2H); ^{13}C NMR (100 MHz, $CDCl_3$) δ: 139.0, 129.0, 127.7, 127.3, 120.1, 69.8, 67.4, 47.5, 33.8, 11.9; LRMS (EI, 70 eV) *m/z* (%): 306 (M^+, 4), 252 (100), 220 (66), 179 (33), 165 (18); HRMS *m/z* (ESI) calcd for $C_{20}H_{23}N_2O$ $(M+H)^+$ 307.1805, found 307.1813.

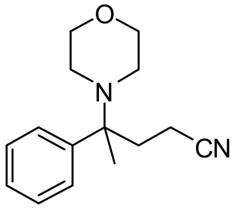

4-Morpholino-4-phenylpentanenitrile (32): Colorless oil; ^{1}H NMR (400 MHz, $CDCl_3$) δ: 7.43 (d, *J* = 8.0 Hz, 2H), 7.35 (t, *J* = 7.6 Hz, 2H), 7.26 (t, *J* = 7.2 Hz, 1H), 3.75-3.66 (m, 4H), 2.52-2.43 (m, 4H), 2.19-2.05 (m, 2H), 2.01-1.87 (m, 2H), 1.40 (s, 3H); ^{13}C NMR (100 MHz, $CDCl_3$) δ: 143.4, 128.5, 127.1, 126.8, 120.0, 67.6, 61.8, 46.7, 37.0, 16.0, 12.2; LRMS (EI, 70 eV) *m/z* (%): 244 (M^+, 3), 229 (13), 190 (100), 158 (10), 117 (10); HRMS *m/z* (ESI) calcd for $C_{15}H_{21}N_2O$ $(M+H)^+$ 245.1648, found 245.1660.

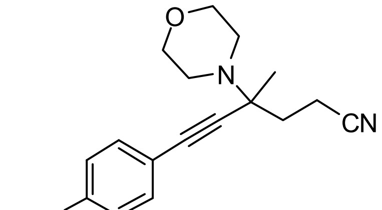

6-(4-Methoxyphenyl)-4-methyl-4-morpholinohex-5-ynenitrile (33): Colorless oil; ^{1}H NMR (400 MHz, $CDCl_3$) δ: 7.35 (d, *J* = 7.6 Hz, 2H), 6.84 (d, *J* = 8.0 Hz, 2H), 3.81 (s, 3H), 3.75 (brs, 4H), 2.70-2.55 (m, 6H), 2.18-2.05 (m, 2H), 1.39 (s, 3H); ^{13}C NMR (100 MHz, $CDCl_3$) δ: 159.6, 133.1, 120.2, 114.5, 113.9, 87.5, 85.7, 67.2, 57.1, 55.3, 47.0, 34.6, 22.7, 12.0; LRMS (EI, 70 eV) *m/z* (%): 298 (M^+, 1), 283 (15),

244 (100), 212 (17), 172 (14); HRMS *m/z* (ESI) calcd for $C_{18}H_{23}N_2O_2$ $(M+H)^+$ 299.1754, found 299.1763.

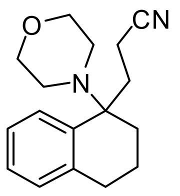

3-(1-Morpholino-1,2,3,4-tetrahydronaphthalen-1-yl)propanenitrile (34): Colorless oil; ^{1}H NMR (400 MHz, $CDCl_3$) δ: 7.45 (t, *J* = 4.4 Hz, 1H), 7.15-7.08 (m, 3H), 3.74-3.65 (m, 4H), 2.79-2.72 (m, 2H), 2.53-2.36 (m, 5H), 2.21-2.02 (m, 4H), 1.88-1.82 (m, 2H), 1.68-1.62 (m, 1H); ^{13}C NMR (100 MHz, $CDCl_3$) δ: 138.3, 137.7, 129.6, 127.3, 126.8, 125.6, 120.3, 67.6, 60.2, 46.4, 35.6, 29.4, 26.7, 20.1, 11.7; LRMS (EI, 70 eV) *m/z* (%): 270 (M^+, 3), 216(100), 184 (63), 143 (41), 128 (31); HRMS *m/z* (ESI) calcd for $C_{17}H_{23}N_2O$ $(M+H)^+$ 271.1805, found 271.1801.

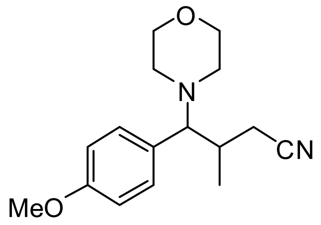

4-(4-Methoxyphenyl)-3-methyl-4-morpholinobutanenitrile (35):

d.r. = 1.3:1 (from ***E*-1o**);

Colorless oil;^{1}H NMR (400 MHz, $CDCl_3$) δ: 7.05-7.02 (m, 4H), 6.89-6.87 (m, 4H), 3.81 (s, 6H), 3.71-3.61 (m, 8H), 3.21-3.14 (m, 2H), 2.65-2.28 (m, 13H), 1.94-1.88 (m, 1H), 1.16 (d, *J* = 6.4 Hz, 3H), 0.91 (d, *J* = 6.4 Hz, 3H); ^{13}C NMR (100 MHz, $CDCl_3$) δ: 159.1, 159.0, 130.1, 130.0, 127.2, 126.4, 119.2, 119.0, 113.6, 113.4, 73.0, 72.8, 67.1 (2C), 55.2, 50.3, 49.6, 30.6, 30.3, 22.4, 20.7, 17.4, 16.8; LRMS (EI, 70 eV) *m/z* (%): 274 (M^+, 1), 206 (100), 188 (4), 148 (8), 121 (8); HRMS *m/z* (ESI) calcd for $C_{16}H_{23}N_2O_2$ $(M+H)^+$ 275.1754, found 275.1766.

d.r. = 1.4:1 (from ***Z*-1p**)

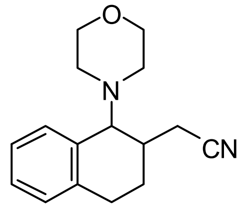

2-(1-Morpholino-1,2,3,4-tetrahydronaphthalen-2-yl)acetonitrile (36): Colorless oil; d.r. = 3:1; ^{1}H NMR (400 MHz, $CDCl_3$) δ: 7.37-7.09 (m, 4H), 3.75-3.52 (m, 5H), 2.89-2.13 (m, 10H), 1.80-1.68 (m, 1H); ^{13}C NMR (100 MHz, $CDCl_3$) δ: 137.5, 136.3, 134.4, 133.6, 130.2, 130.1, 129.1, 128.9, 127.6, 127.2, 125.8, 125.5, 119.8, 118.9, 67.7, 67.6, 66.8, 63.5, 54.4, 50.8, 38.2, 34.3, 28.2, 27.1, 25.8, 23.7, 21.9, 20.7; LRMS (EI, 70 eV) *m/z* (%): 256 (M^+, 7), 188 (98), 169 (33), 129 (100), 115 (14); HRMS *m/z* (ESI) calcd for $C_{16}H_{21}N_2O$ $(M+H)^+$ 257.1648, found 257.1656.

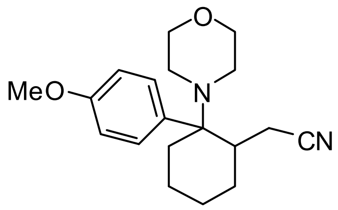

2-(2-(4-Methoxyphenyl)-2-morpholinocyclohexyl)acetonitrile (37): Colorless oil; d.r. = 1:1; ^{1}H NMR (400 MHz, $CDCl_3$) δ: 7.15 (d, *J* = 8.4 Hz, 4H), 6.93-6.88 (m, 4H), 3.82 (s, 3H), 3.81 (s, 3H), 3.66-3.61 (m, 8H), 3.14-3.02 (m, 2H), 2.80 (d, *J* = 7.2 Hz, 1H), 2.57-2.04 (m, 13H), 1.93-1.83 (m, 2H), 1.72-1.15 (m, 12H); ^{13}C NMR (100 MHz, $CDCl_3$) δ: 158.3, 158.2, 130.0, 128.2, 127.8, 126.6, 120.8, 119.9, 113.3, 67.6, 67.4, 62.4, 61.8, 55.1, 45.5, 45.3, 35.6, 34.1, 27.0, 25.8, 24.8, 23.5, 21.9, 21.0, 20.2, 19.3, 17.6, 15.5; LRMS (EI, 70 eV) *m/z* (%): 314 (M^+, 43), 274 (15), 232 (73), 187 (25), 147 (29), 121 (100); HRMS *m/z* (ESI) calcd for $C_{19}H_{27}N_2O_2$ $(M+H)^+$ 315.2067, found 315.2075.

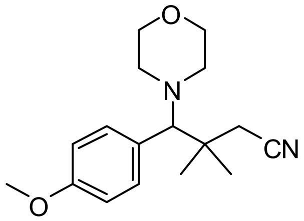

4-(4-Methoxyphenyl)-3,3-dimethyl-4-morpholinobutanenitrile (38): Colorless oil; ^{1}H NMR (400 MHz, $CDCl_3$) δ: 7.15 (d, *J* = 8.4 Hz, 2H), 6.88 (d, *J* = 8.0 Hz, 2H), 3.82 (s, 3H), 3.72-3.64 (m, 4H), 3.30 (s, 1H), 2.68-2.62 (m, 3H), 2.36-2.31 (m, 3H), 1.30 (s,

3H), 1.00 (s, 3H); ^{13}C NMR (100 MHz, $CDCl_3$) δ: 158.9, 131.7, 126.8, 119.0, 113.3, 76.7, 67.4, 55.2, 53.0, 39.4, 30.5, 25.9, 24.6; LRMS (EI, 70 eV) *m/z* (%): 288 (M^+, 1), 206 (100), 162 (3), 121 (9); HRMS *m/z* (ESI) calcd for $C_{17}H_{25}N_2O_2$ $(M+H)^+$ 289.1911, found 289.1919.

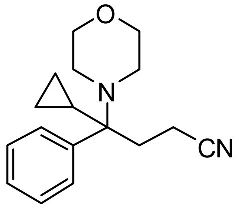

4-Cyclopropyl-4-morpholino-4-phenylbutanenitrile (39): White solid; mp 106.1-107.1 ºC (uncorrected); ^{1}H NMR (400 MHz, $CDCl_3$) δ: 7.64 (d, *J* = 8.0 Hz, 2H), 7.34 (t, *J* = 7.2 Hz, 2H), 7.27-7.23 (m, 1H), 3.78-3.67 (m, 4H), 2.80-2.76 (m, 2H), 2.63-2.59 (m, 2H), 240-2.32 (m, 1H), 2.09-2.00 (m, 1H), 1.92-1.84 (m, 1H), 1.50-1.42 (m, 1H), 1.26-1.17 (m, 1H), 0.74-0.66 (m, 2H), 0.61-0.48 (m, 2H); ^{13}C NMR (100 MHz, $CDCl_3$) δ: 144.0, 128.2, 127.5, 127.0, 120.3, 67.8, 63.0, 47.4, 28.8, 14.1, 11.2, 1.3, 0.7; LRMS (EI, 70 eV) *m/z* (%): 270 (M^+, 1), 229 (15), 216 (100), 141 (34); HRMS *m/z* (ESI) calcd for $C_{17}H_{23}N_2O$ $(M+H)^+$ 271.1805, found 271.1813.

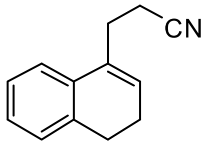

3-(3,4-Dihydronaphthalen-1-yl)propanenitrile (40)[8]: Colorless oil; ^{1}H NMR (400 MHz, $CDCl_3$) δ: 7.25-7.11 (m, 4H), 6.00 (t, *J* = 4.4 Hz, 1H), 2.82-2.74 (m, 4H), 2.56 (t, *J* = 7.6 Hz, 2H), 2.31-2.26 (m, 2H); ^{13}C NMR (100 MHz, $CDCl_3$) δ: 136.8, 133.1, 133.0, 128.0, 127.4, 127.2, 126.5, 121.7, 119.3, 28.6, 28.0, 22.9, 16.7.

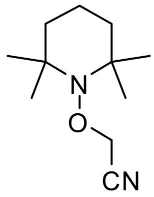

2-(2,2,6,6-Tetramethylpiperidin-1-yloxy)acetonitrile (41)[9]: Colorless oil; ^{1}H NMR (400 MHz, $CDCl_3$) δ: 4.51 (s, 2H), 1.58-1.31 (m, 6H), 1.20 (s, 6H), 1.10 (s, 6H); ^{13}C NMR (100 MHz, $CDCl_3$) δ: 116.0, 62.6, 60.3, 39.5, 32.9, 19.8, 16.8.

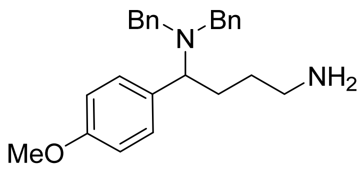

N^1,N^1-dibenzyl-1-(4-methoxyphenyl)butane-1,4-diamine (42):[10]

To a suspension of $LiAlH_4$ (0.3 mmol) in THF (1 mL) at 0 °C was slowly added a solution of **4** (0.15 mmol) in THF (1.0 mL), then, the mixture was warmed to room temperature, and stirred for an additional 4 h. Water was cautiously added until gas evolution ceased, followed by addition of 1M NaOH until the solution became basic (pH > 9). The aqueous layer was extracted with EtOAc (3×10 mL). The combined organic layer was washed with H_2O (2×10 mL) and brine (10 mL), and dried (anhyd Na_2SO_4). The residue was further purified by flash chromatography (CH_2Cl_2/MeOH/NH_4OH, 90/10/1 to 80/20/1), yielding the corresponding amine **42**.

Colorless oil; ^1H NMR (400 MHz, $CDCl_3$) δ: 7.39-7.28 (m, 8H), 7.24-7.12 (m, 4H), 6.90 (d, *J* = 8.0 Hz, 2H), 3.80-3.77 (m, 7H), 3.60 (t, *J* = 7.2 Hz, 1H), 3.08 (d, *J* = 13.6 Hz, 2H), 2.59 (brs, 2H), 2.10 (dd, *J* = 9.6, 17.2 Hz, 1H),1.67 (brs, 2H), 1.42 (brs, 1H); ^{13}C NMR (100 MHz, $CDCl_3$) δ: 158.5, 140.3, 130.4, 129.9, 128.7, 128.2, 126.7, 113.2, 60.7, 55.1, 53.5, 41.3, 29.1, 28.8; HRMS *m/z* (ESI) calcd for $C_{25}H_{31}N_2O$ $(M+H)^+$ 375.2431, found 375.2442.

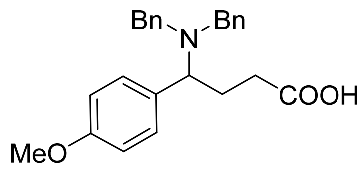

4-(Dibenzylamino)-4-(4-methoxyphenyl)butanoic acid (43):[10]

The mixture of **4** (0.15 mmol) and NaOH (0.15 mmol) in ethanol/water (1:1, 2 mL) was heated to reflux for 72 h. After cooling to room tempertrue, the ethanol was removed in vacuo and the aqueous phase was acidified by

1 M HCl (to pH 5.5-6) and extracted with EtOAc (3×10 mL). The organic layer was dried with anhydrous Na_2SO_4. The solvent was evaperated under reduced pressure. The residue was purified by flash chromatography(EtOAc/Hexane, 1/2), yielding the corresponding acid **43**

Colorless oil; ^{1}H NMR (400 MHz, $CDCl_3$) δ: 7.39 (d, *J* = 7.2 Hz, 4H), 7.31 (t, *J* = 7.6 Hz, 4H), 7.22 (t, *J* = 7.2 Hz, 2H), 7.16 (d, *J* = 8.4 Hz, 2H), 6.93 (d, *J* = 8.0 Hz, 2H), 3.87 (d,*J* = 13.6 Hz, 2H), 3.83 (s, 3H), 3.72 (t, *J* = 7.2 Hz, 1H), 3.10 (d, *J* = 13.6 Hz, 2H), 2.55-2.35 (m, 3H),2.01-1.93 (m, 1H); ^{13}C NMR (100 MHz, $CDCl_3$) δ: 179.2, 158.9, 139.0, 130.1, 129.2, 129.0, 128.4, 127.0, 113.4, 60.9, 55.2, 53.6, 32.4, 26.4; HRMS *m/z* (ESI) calcd for $C_{25}H_{28}NO_3$ $(M+H)^+$ 390.2064, found 390.2076.

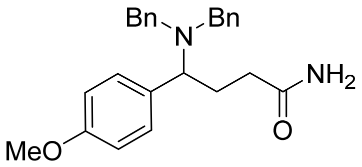

4-(Dibenzylamino)-4-(4-methoxyphenyl)butanamide (44):[11]

Nitrile **4** (0.15 mmol) was dissolved in a mixture of water/THF (1:3,2 mL). Acetamide (4 equiv) and $PdCl_2$ (0.015 mmol) were added, and the mixture was stirred at room temperature for 24 h. The reaction was diluted with EtOAc and washed with $NaHCO_3$ and brine. After dried over Na_2SO_4, it was concentrated and purified by flash chromatography (EtOAc/Hexane, 1/2) to give the compound amide **44**.

Colorless oil; ^{1}H NMR (400 MHz, $CDCl_3$) δ: 7.37-7.14 (m, 12H), 7.20 (d, *J* = 8.0 Hz, 2H), 5.52 (s, 1H), 5.20 (s, 1H), 3.83 (s, 3H), 3.80 (d, *J* = 13.6 Hz, 2H), 3.62 (t, *J* = 7.2 Hz, 1H), 3.12 (d, *J* = 13.6 Hz, 2H), 2.40-2.29 (m, 2H), 2.17-2.04 (m, 2H); ^{13}C NMR (100 MHz, $CDCl_3$) δ: 175.4, 158.7, 140.1, 130.0, 129.9, 128.8, 128.2, 126.8, 113.4,

60.7, 55.2, 53.5, 33.3, 27.2; HRMS *m/z* (ESI) calcd for $C_{25}H_{29}N_2O_2$ $(M+H)^+$ 389.2224, found 389.2232.

Supplementary References

(1) Gajare, A. S., Jensen, R. S., Toyota, K., Yoshifuji, M. & Ozawa, F. Low-coordinated diphosphinidenecyclobutene ligands: a New entry for stille cross-coupling of aryl bromides. *Synlett* 144-148 (2005).

(2) Larsen, C. R. &Grotjahn, D. B. Stereoselective alkene isomerization over one position. *J. Am. Chem. Soc.* **134,** 10357-10360 (2012).

(3) Lee, C.-L. *et al.* First total synthesis of antrocamphin A and its analogs as anti-inflammatory and anti-platelet aggregation agents. *Org. Biomol. Chem.* **9**, 70-73 (2011).

(4) Blum, T. R., Zhu, Y., Nordeen, S. A. & Yoon, T. P. Photocatalytic synthesis of dihydrobenzofurans by oxidative [3+2] cycloaddition of phenols. *Angew. Chem. Int. Ed.* **53,** 11056-11059 (2014).

(5) Ojha, D. P. & Prabhu, K. R. Palladium catalyzed coupling of tosylhydrazones with aryl and heteroaryl halides in the absence of external ligang: synthesis of substituted olefins *J. Org. Chem.* **77,** 11027-11033 (2012).

(6) Yan, J., Jin, S. & Wang, B. A novel redox-sensitive protecting group for boronic acids, MPMP-diol. *Tetrahedron Lett.* **46,** 8503-8505 (2005).

(7) Liwosz, T. W. & Chemler, S. R. Copper-catalyzed oxidative amination and allylic amination of alkenes. *Chem.-Eur. J.* **19,** 12771-12777 (2013).

(8) Chatalova-Sazepin, C., Wang, Q. M., Sammis, G. & Zhu, J. Copper-catalyzed intermolecular carboetherification of unactivated alkenes by alkyl nitriles and alcohols. *Angew. Chem. Int. Ed.* **54,** 5443-5446 (2015).

(9) Bunescu, A., Wang, Q. & Zhu, J. Synthesis of functionalized epoxides by copper-catalyzed alkylative epoxidation of allylic Alcohols with alkyl nitriles. *Org. Lett.* **17,** 1890-1893 (2015).

(10) Rappoport, Z. *The Chemistry of the Cyano Group*. (Wiley, London, 1970).

(11) Maffioli, S. I., Marzorati, E. &Marazzi, A. Mild and reversible dehydration of primary amides with $PdCl_2$ in aqueous acetonitrile. *Org. Lett.* **7,** 5237-5239 (2005).

Supplementary Figures

Supplementary Figure 1. ^{1}H, ^{13}C-NMR spectra of product **4**

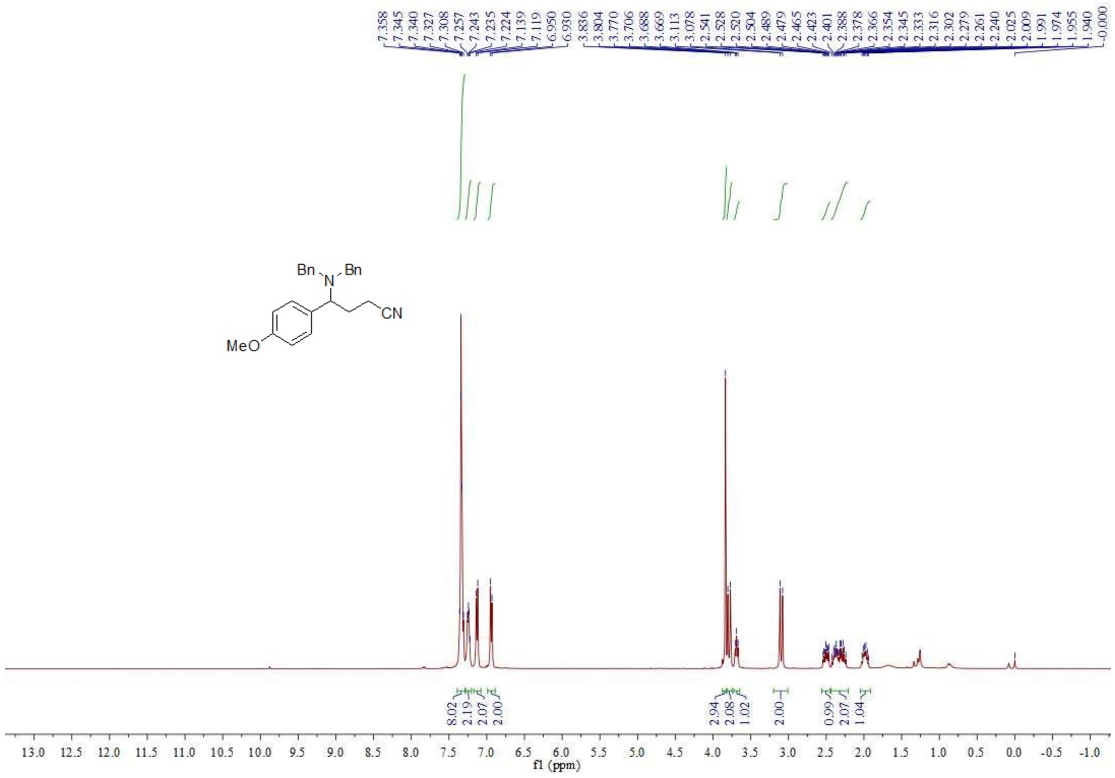

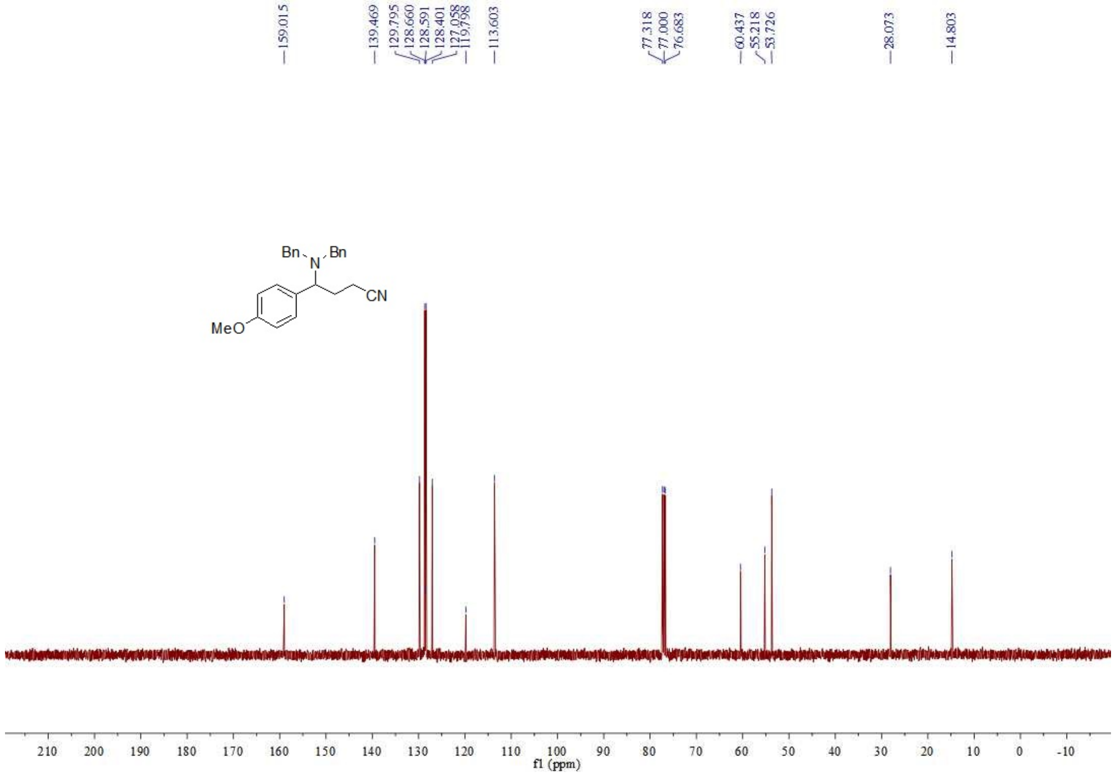

Supplementary Figure 2. ^{1}H, ^{13}C-NMR spectra of product **5**

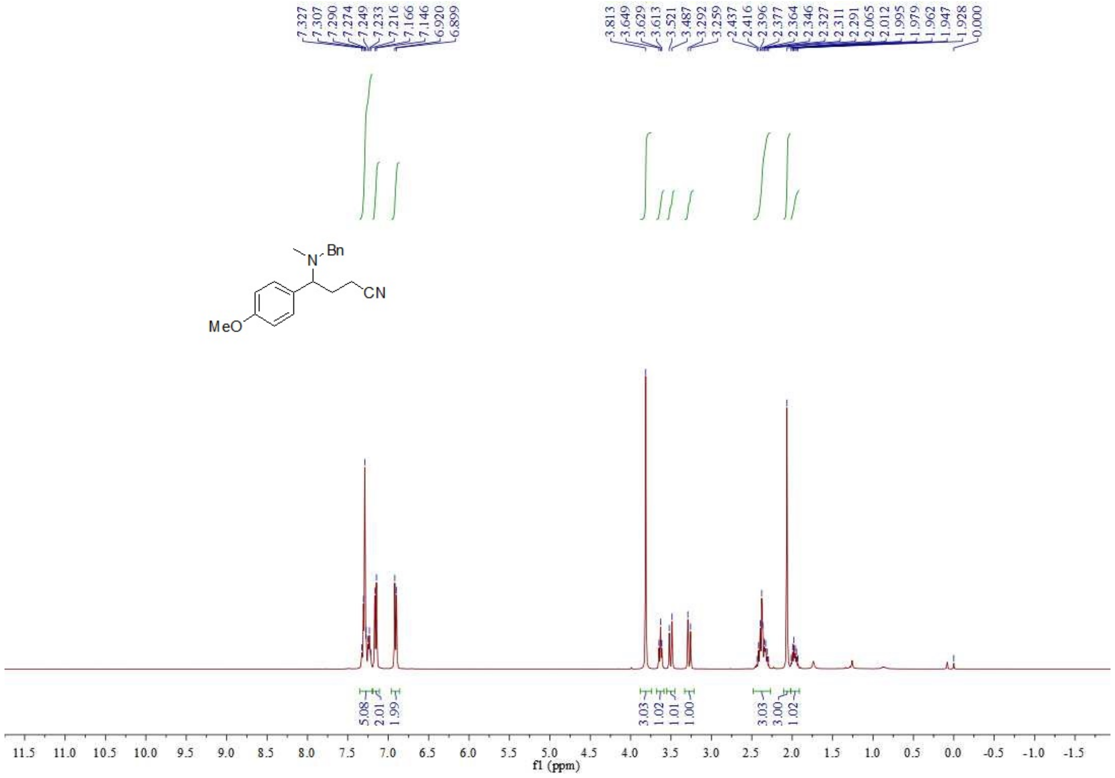

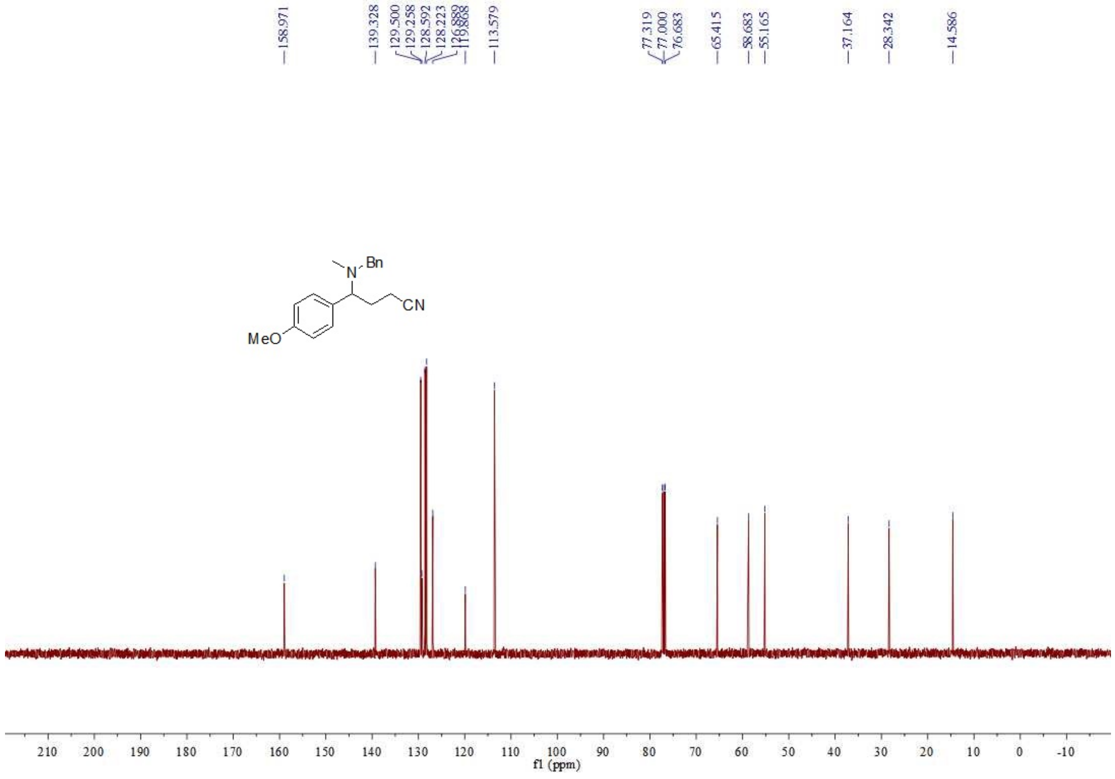

Supplementary Figure 3. ^{1}H, ^{13}C-NMR spectra of product **6**

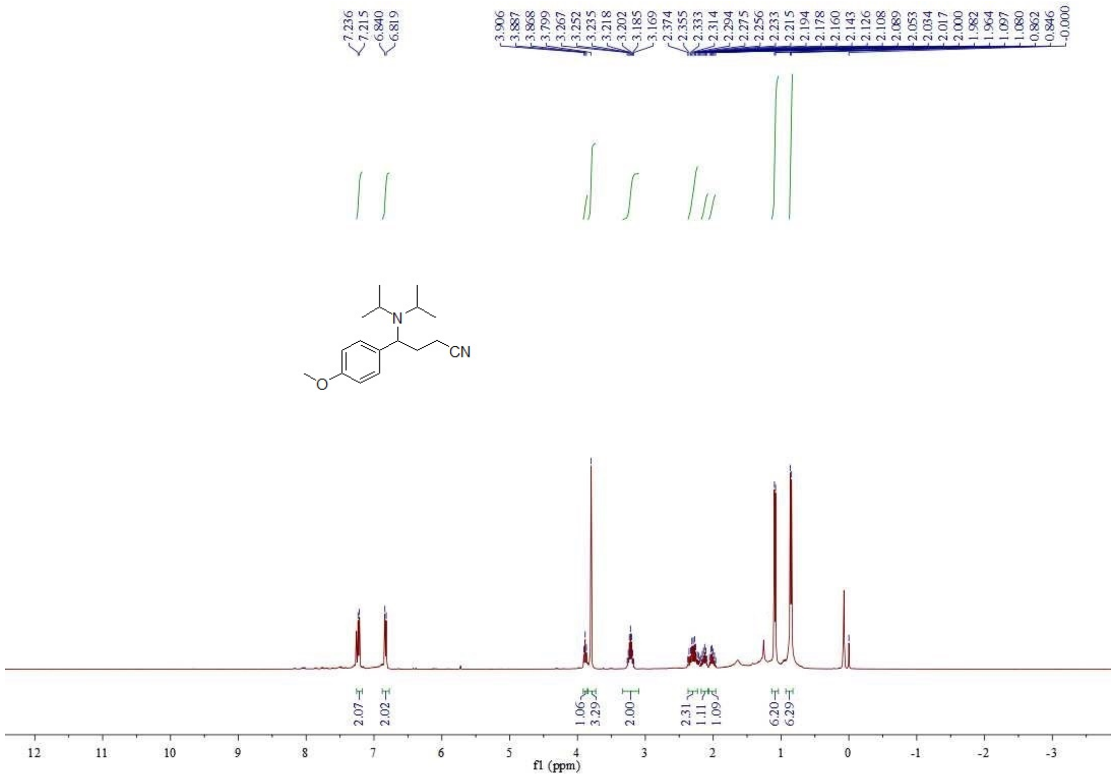

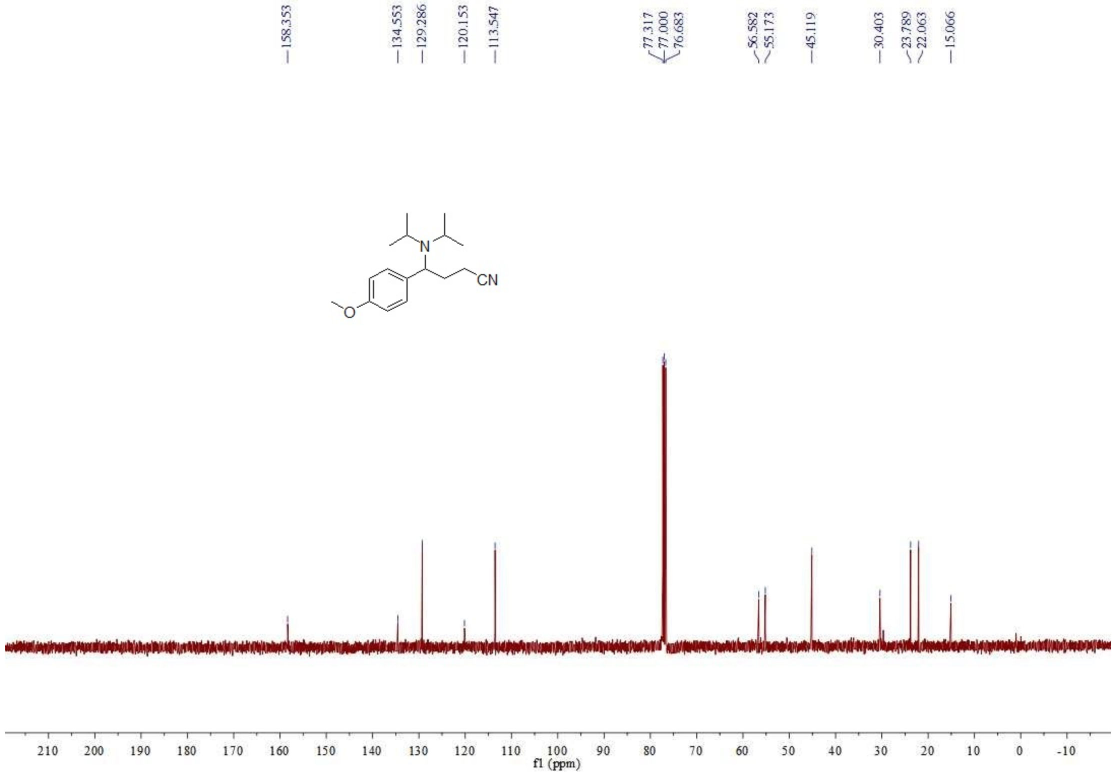

Supplementary Figure 4. ^{1}H, ^{13}C-NMR spectra of product **7**

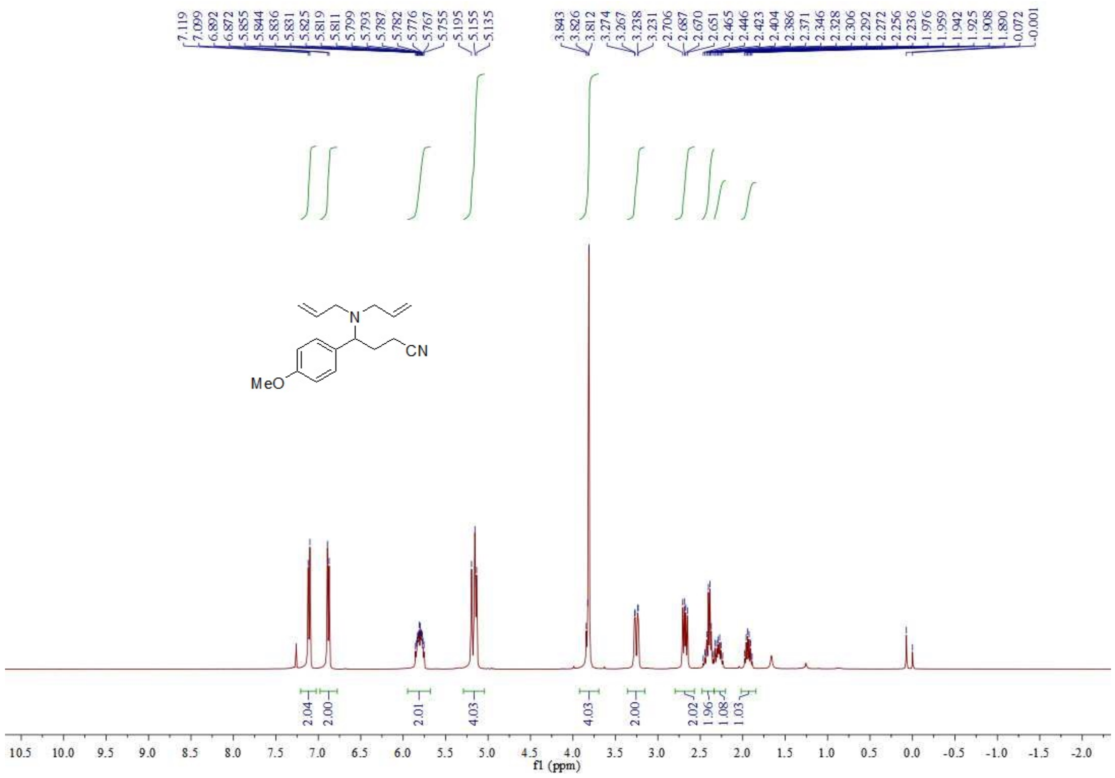

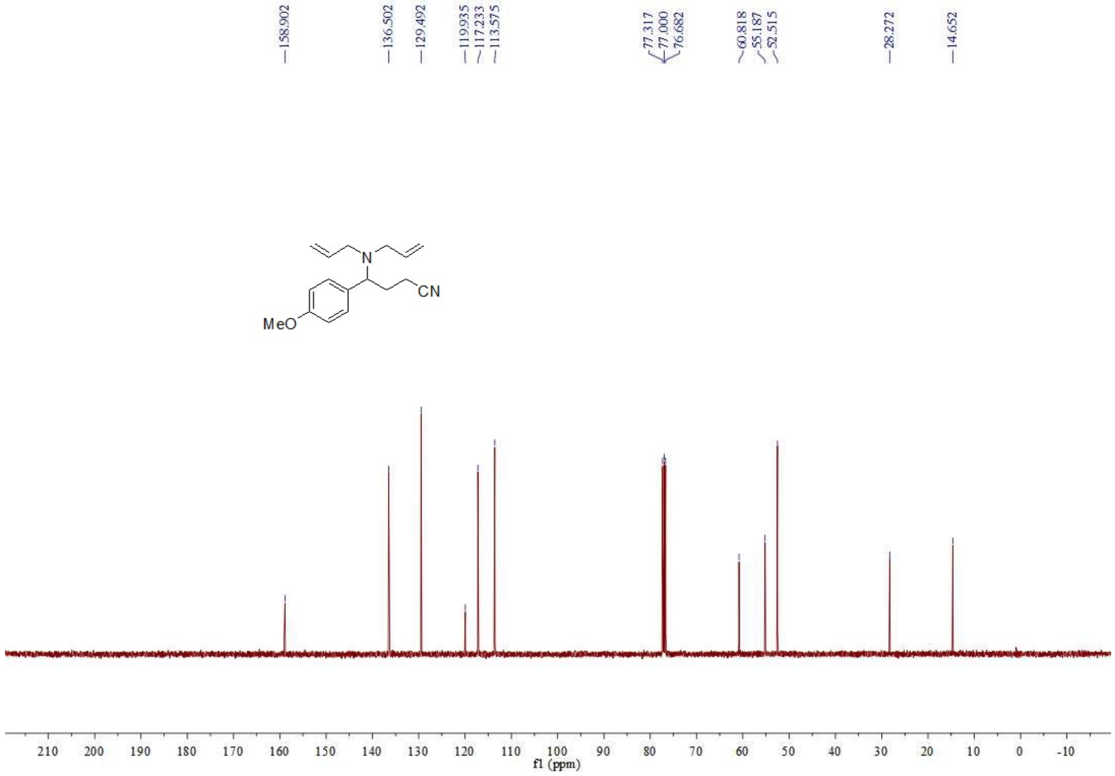

Supplementary Figure 5. ^{1}H, ^{13}C-NMR spectra of product **8**

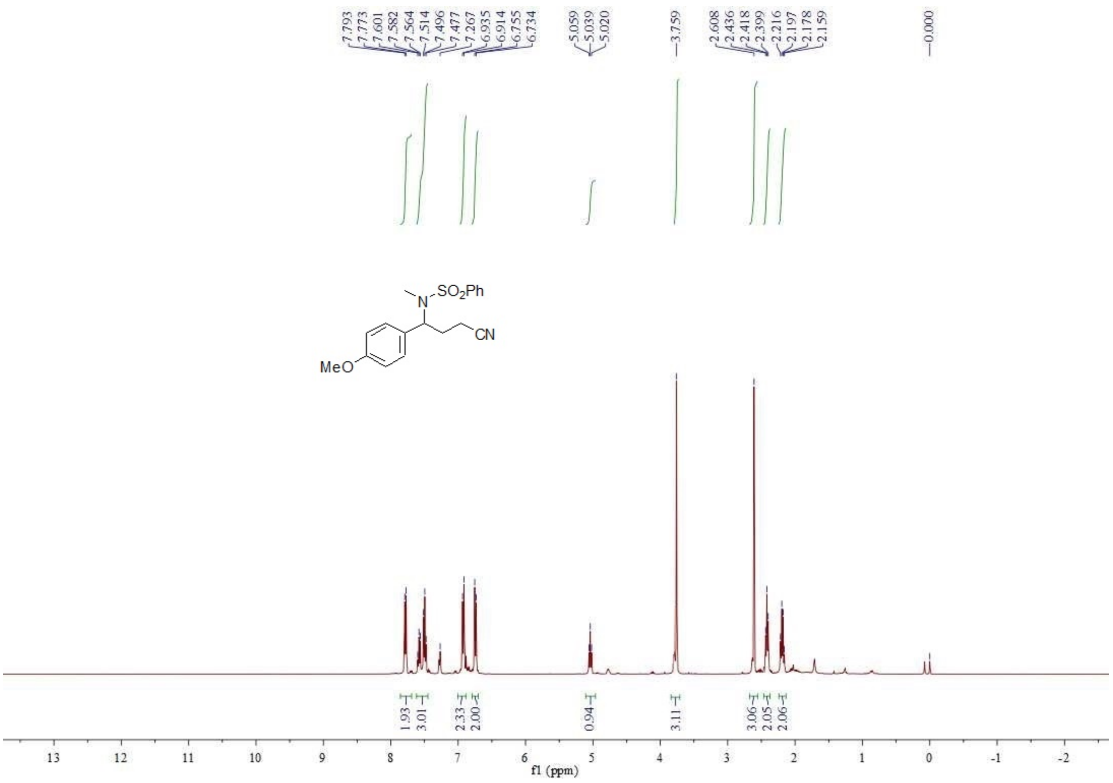

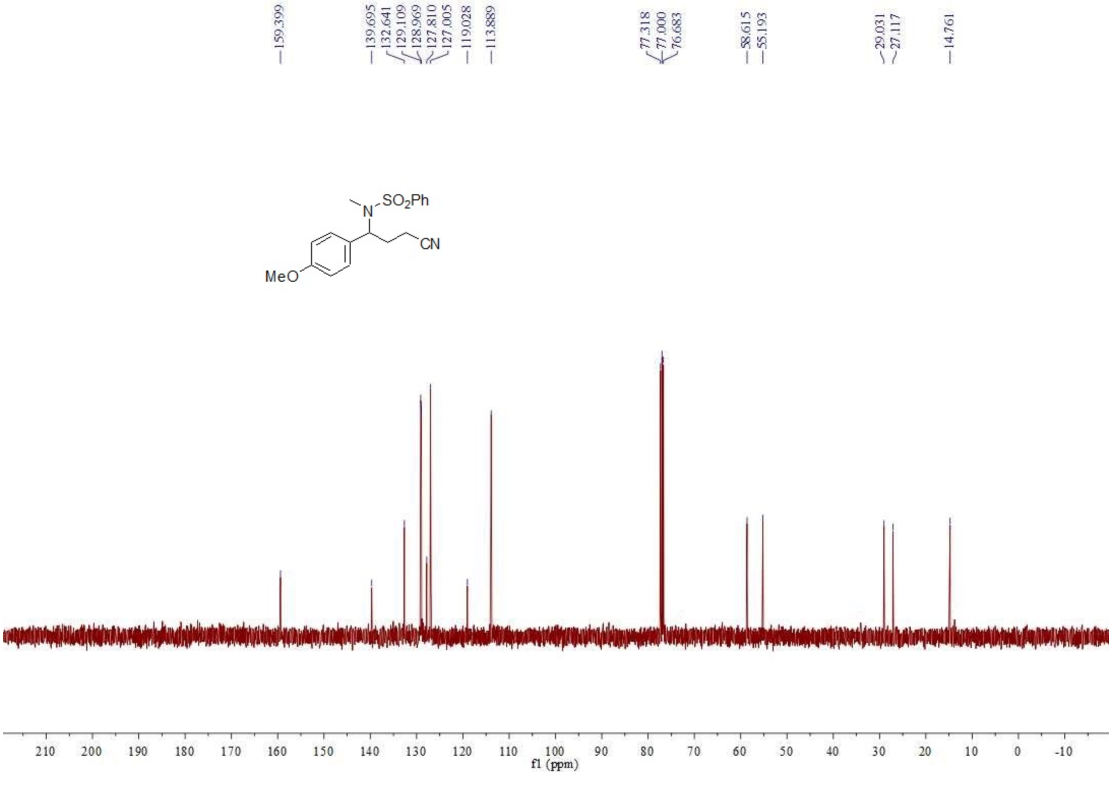

Supplementary Figure 6. ^{1}H, ^{13}C-NMR spectra of product **9**

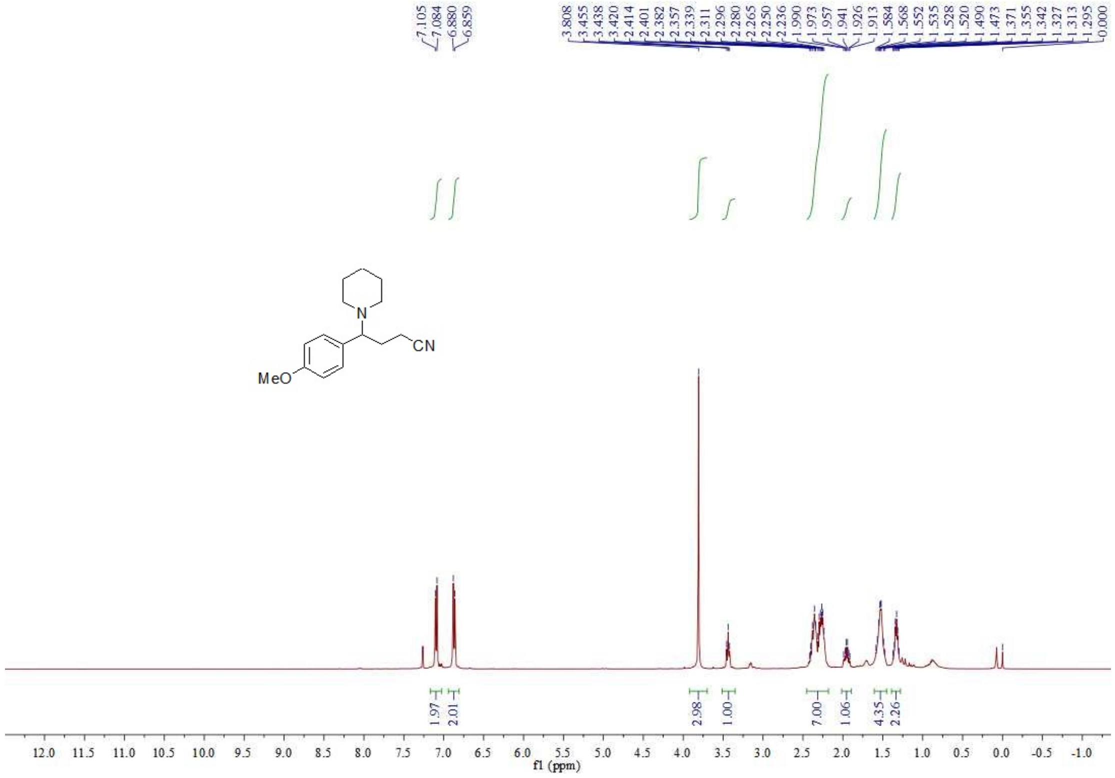

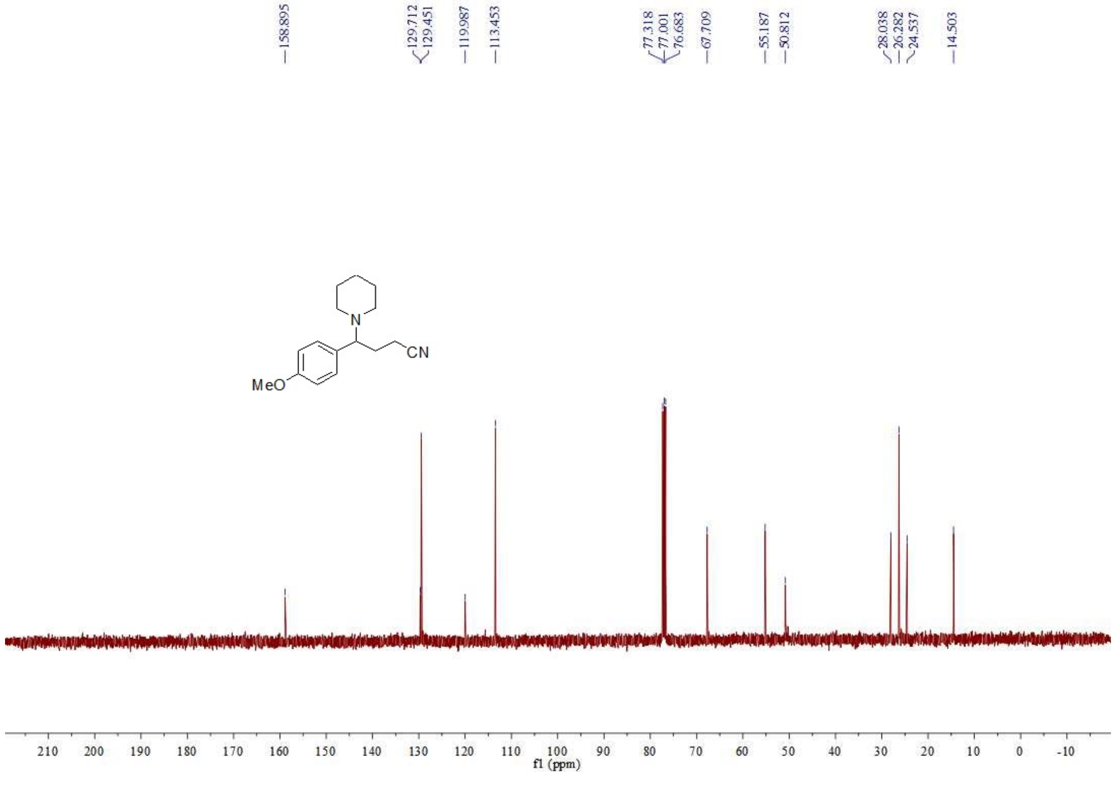

Supplementary Figure 7. ^{1}H, ^{13}C-NMR spectra of product **10**

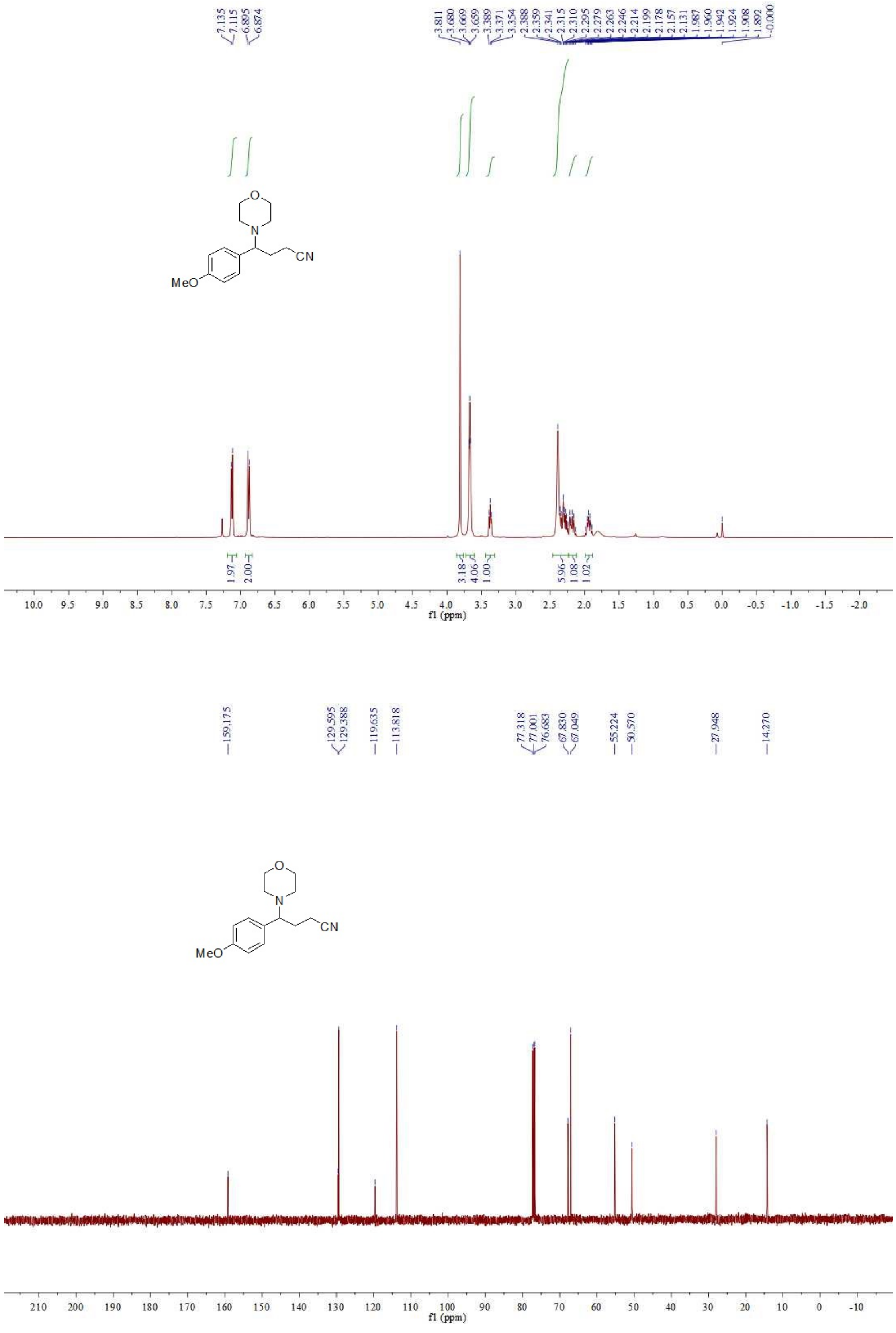

Supplementary Figure 8. ^{1}H, ^{13}C-NMR spectra of product **11**

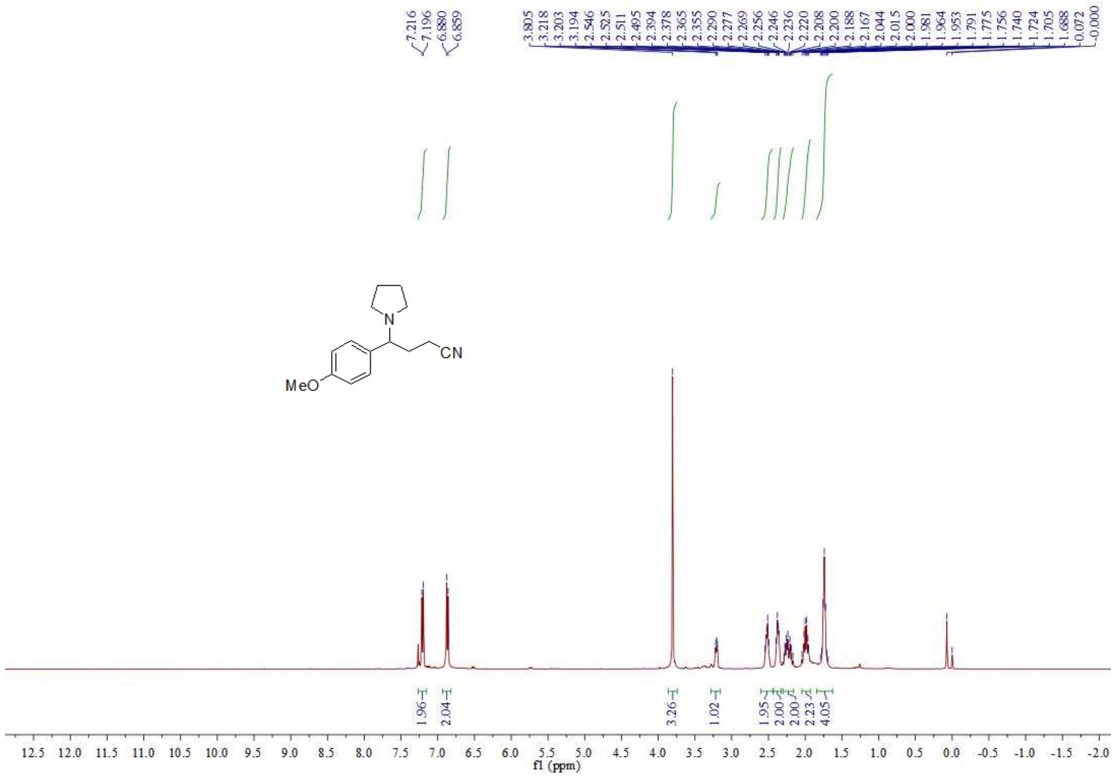

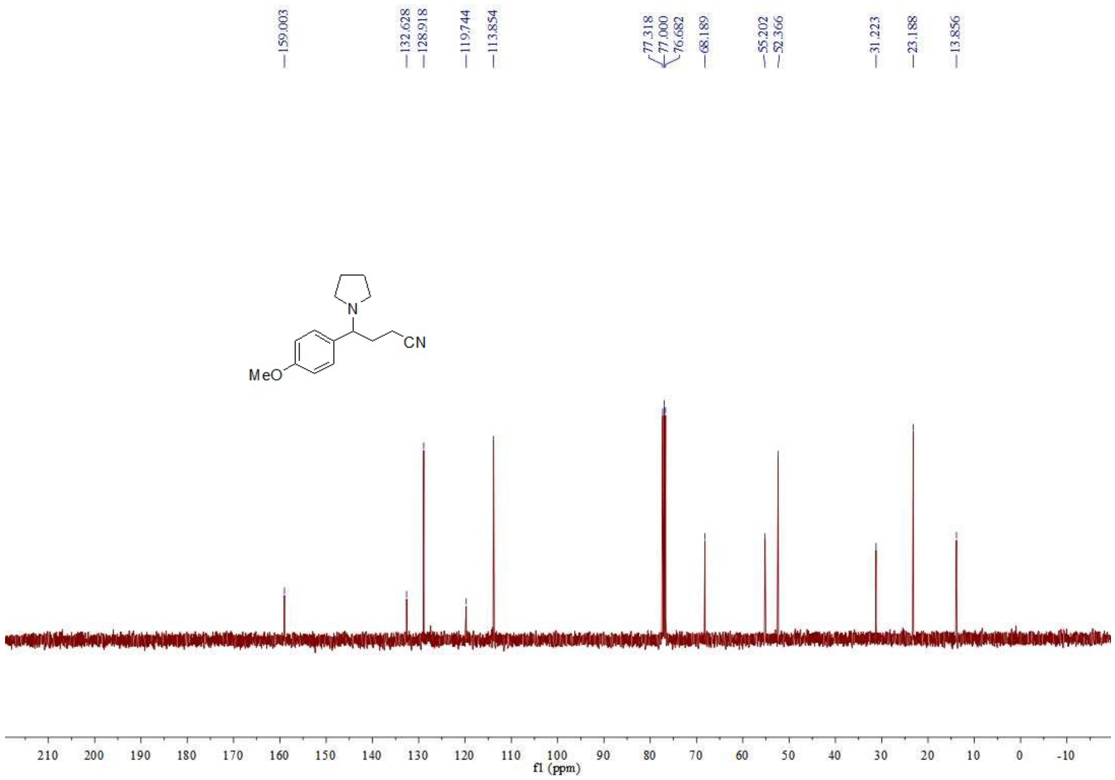

Supplementary Figure 9. ^{1}H, ^{13}C-NMR spectra of product **12**

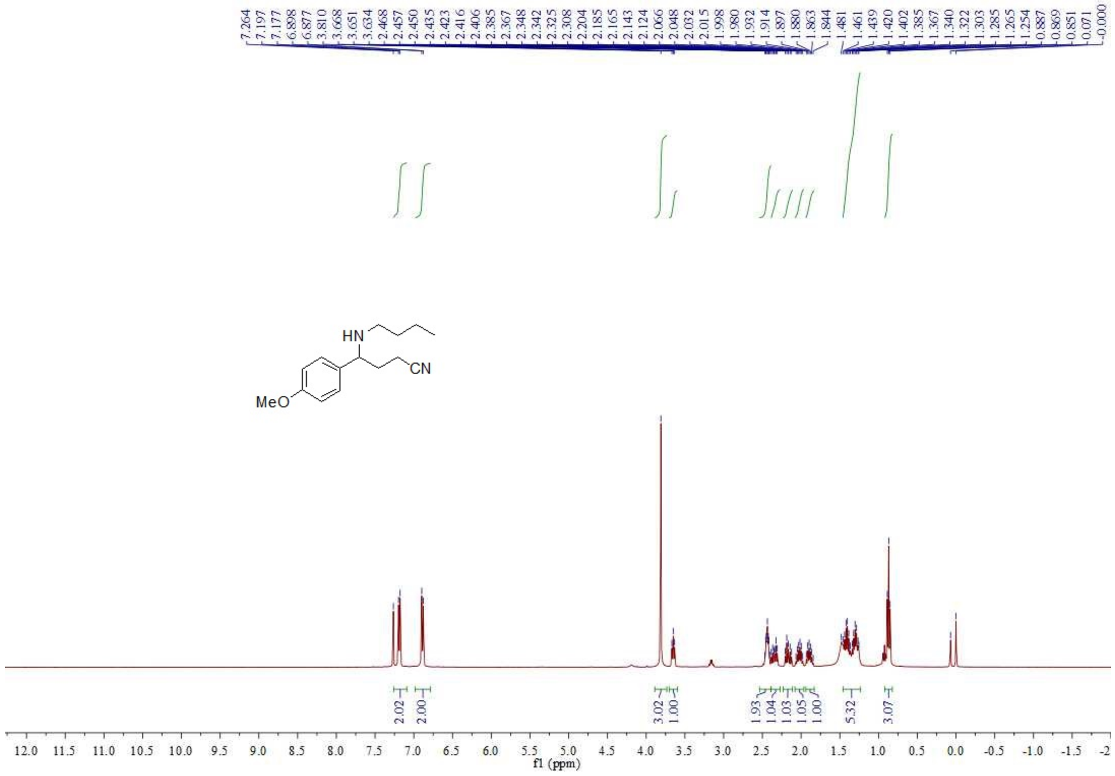

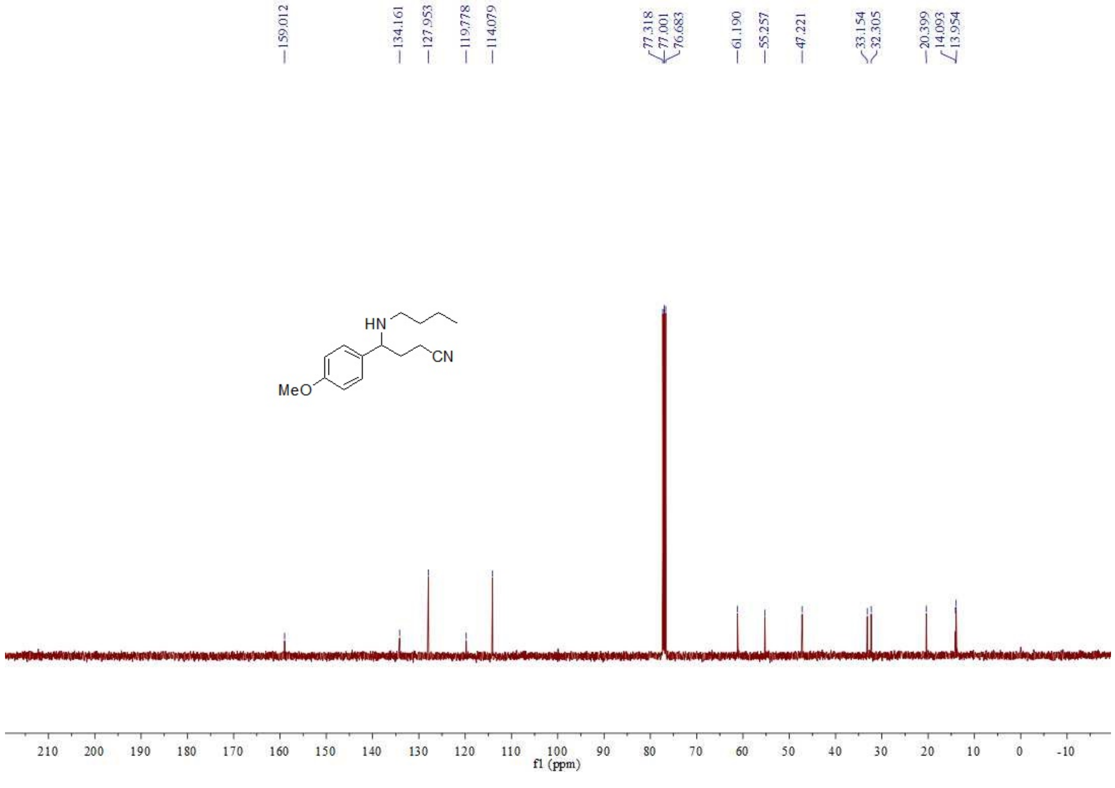

Supplementary Figure 10. ^{1}H, ^{13}C-NMR spectra of product **13**

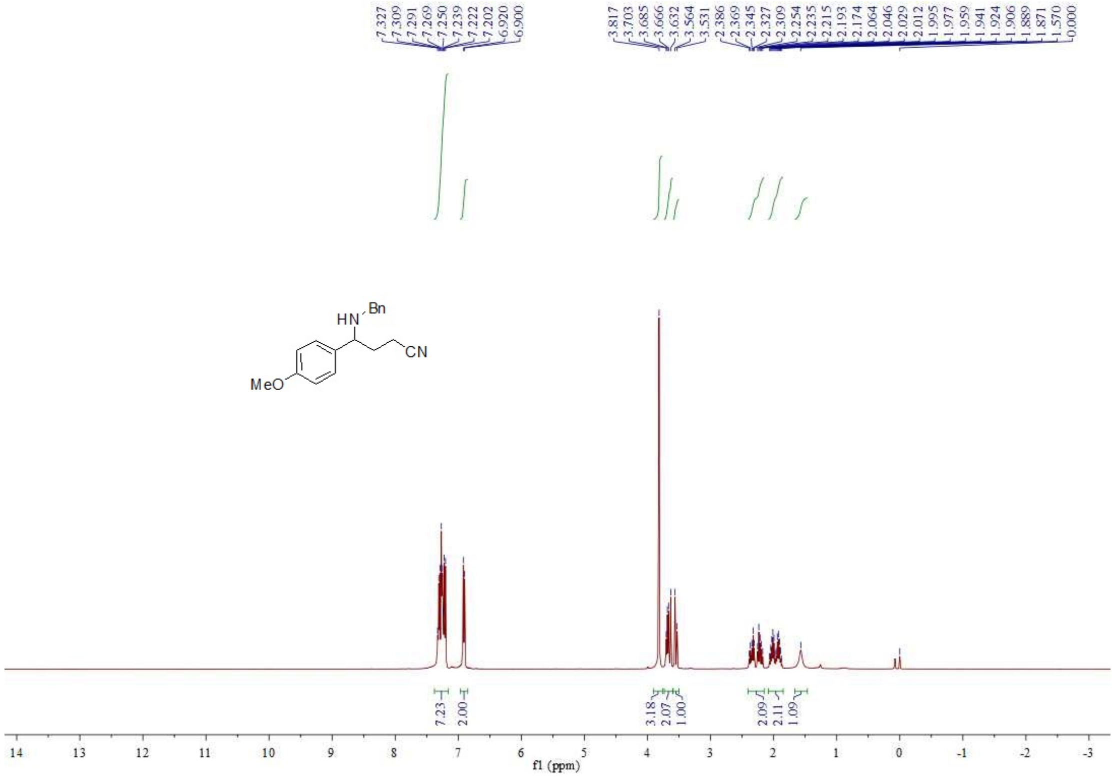

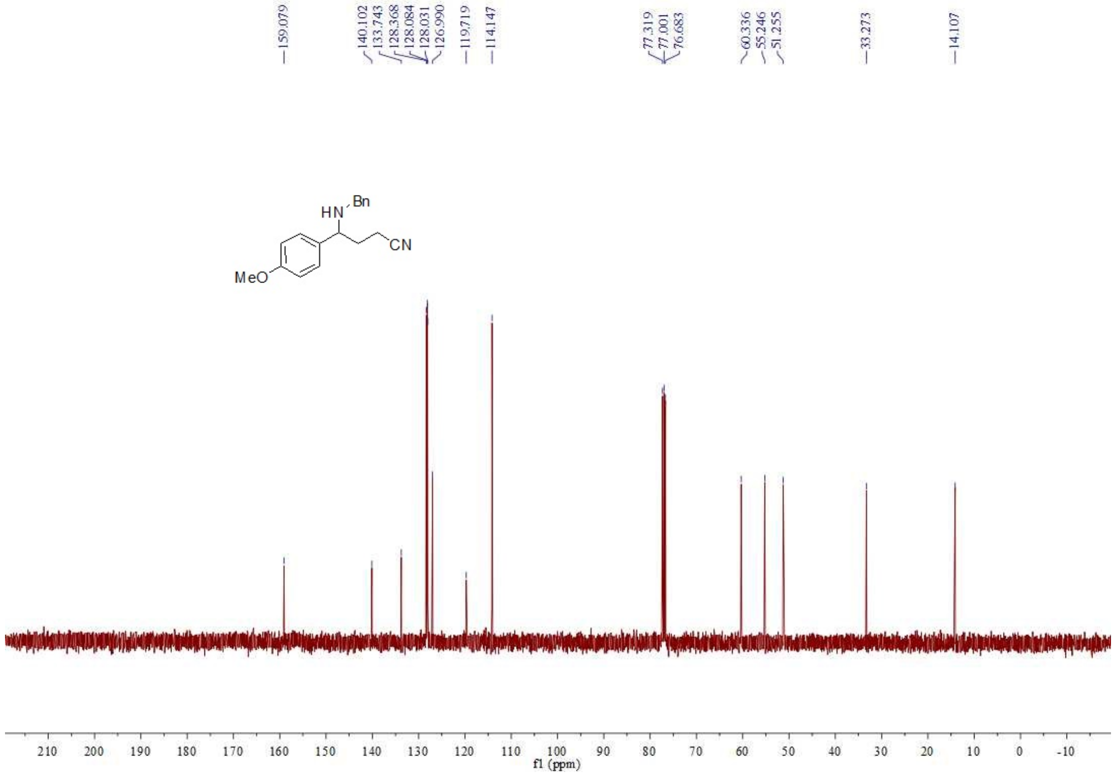

Supplementary Figure 11. ^{1}H, ^{13}C-NMR spectra of product **14**

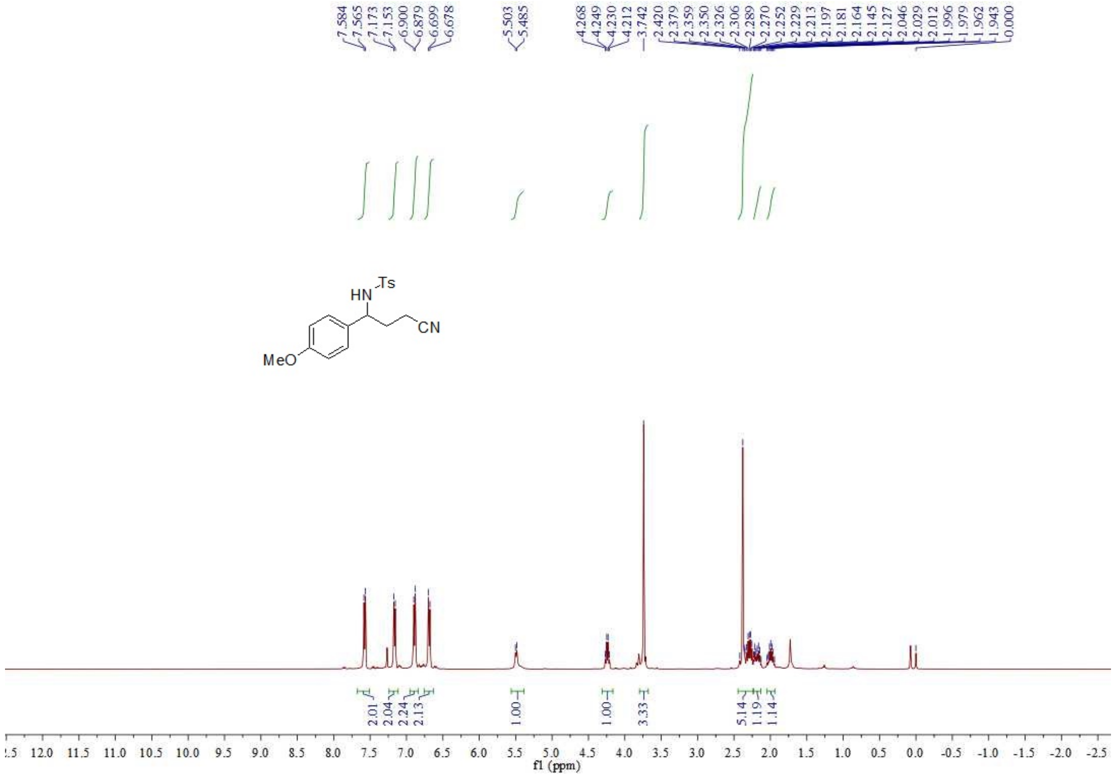

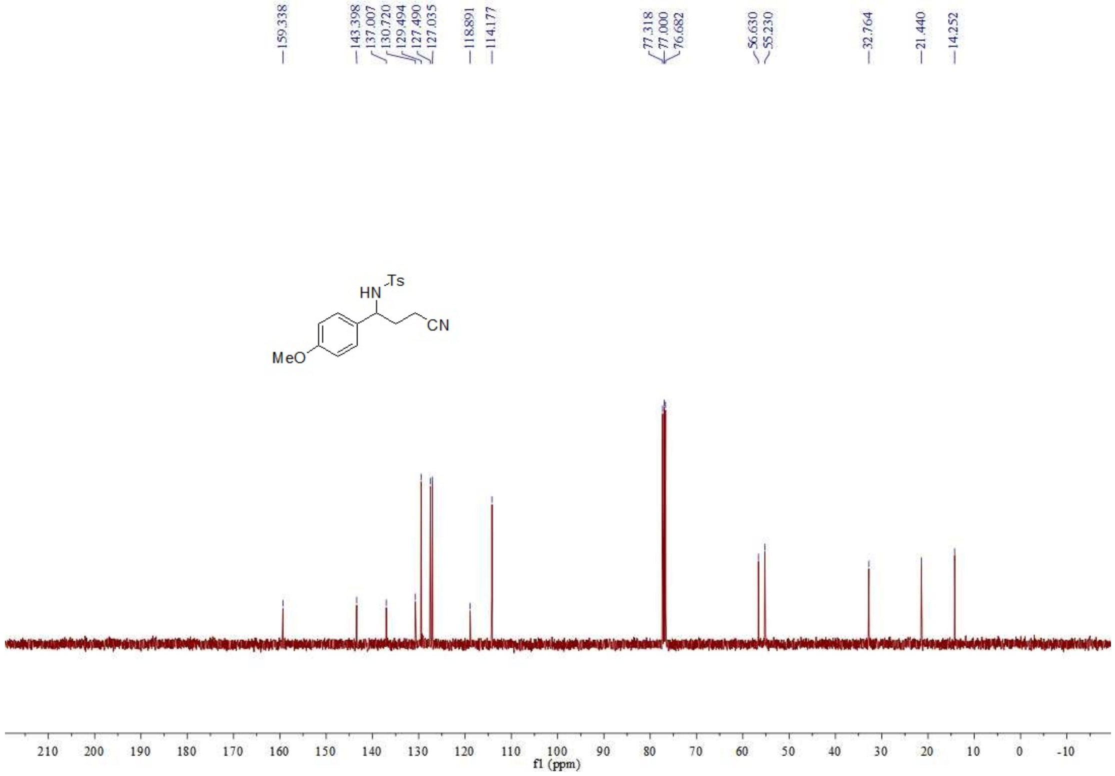

Supplementary Figure 12. ^{1}H, ^{13}C-NMR spectra of product **15**

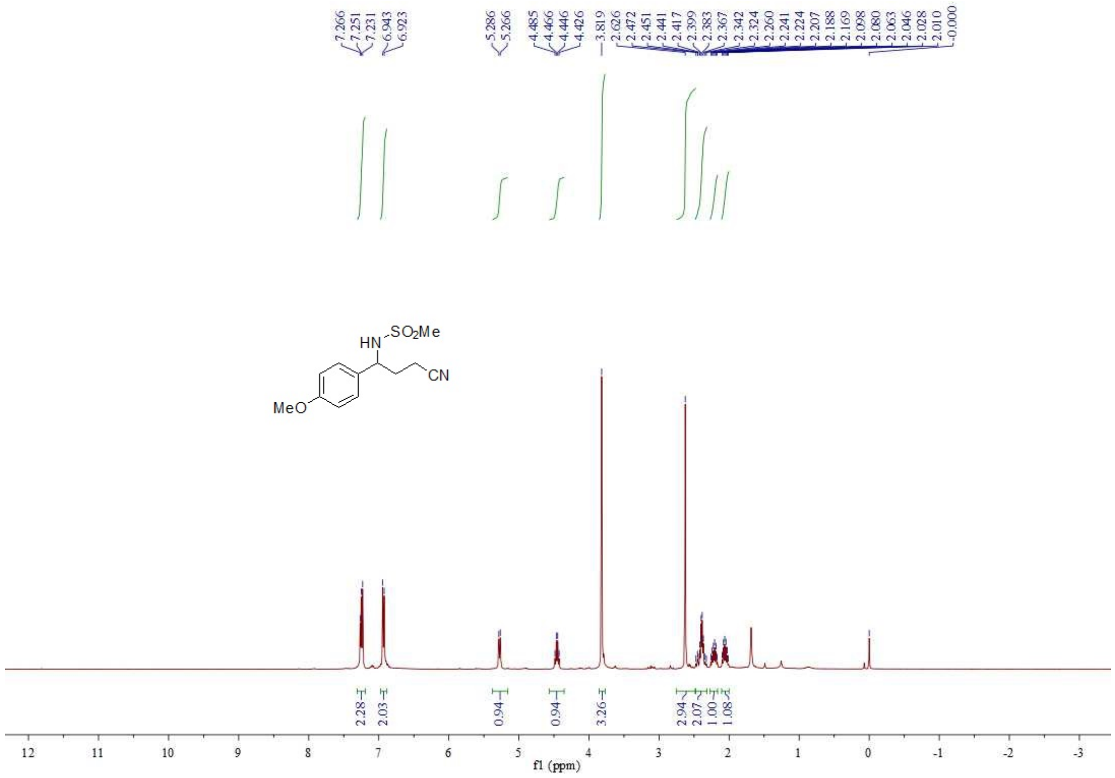

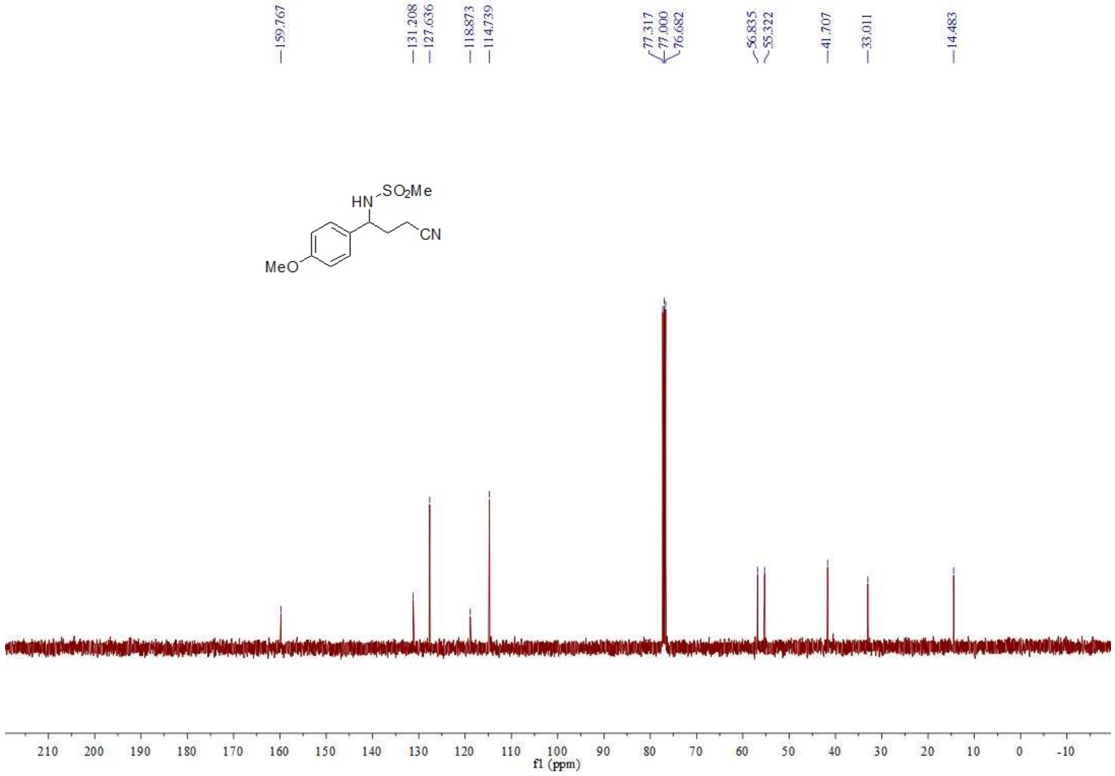

Supplementary Figure 13. ^1H, ^{13}C-NMR spectra of product **17**

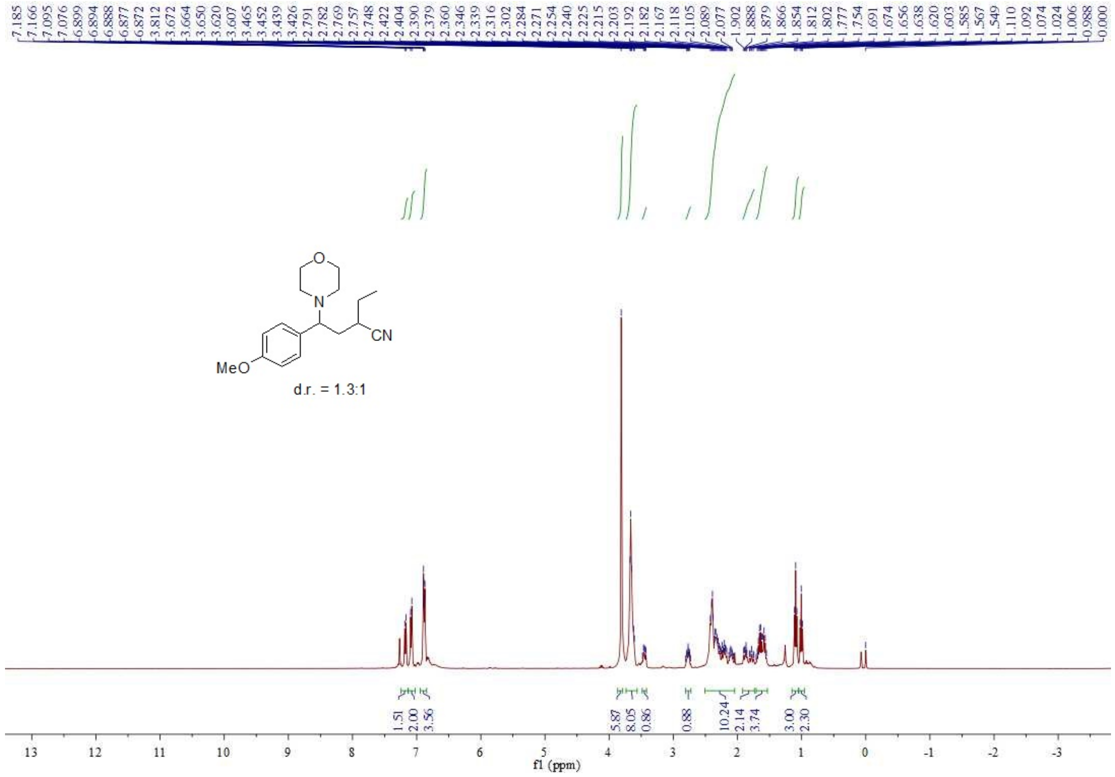

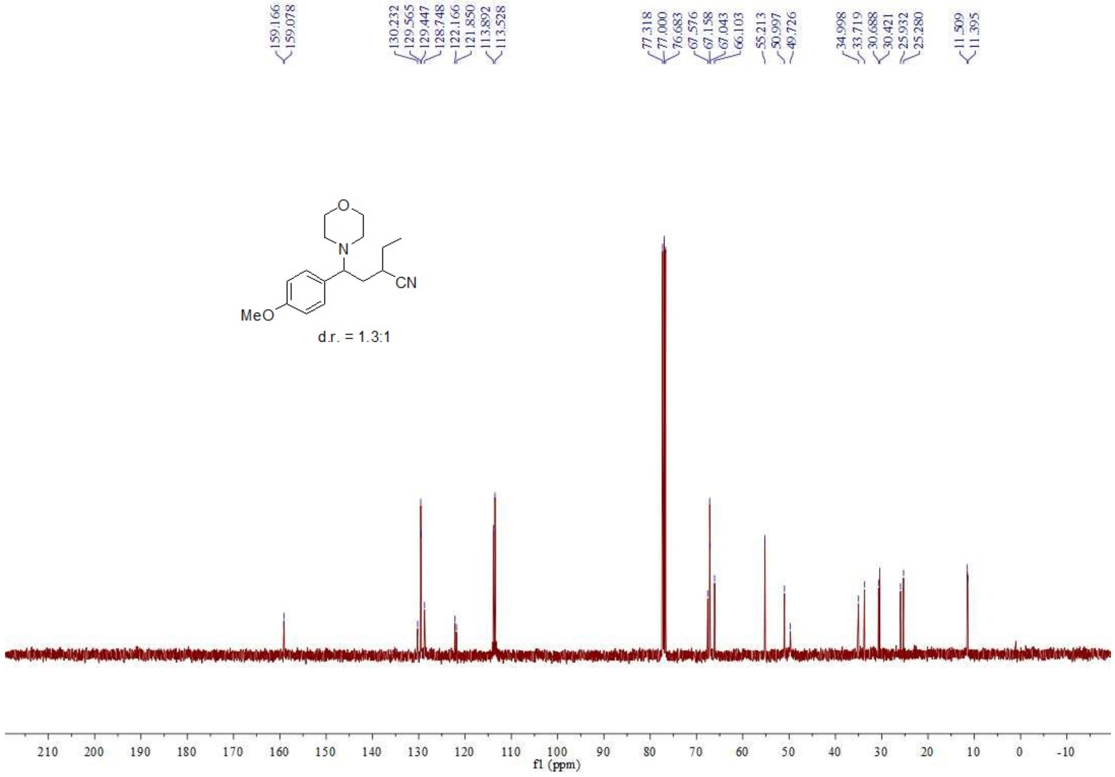

Supplementary Figure 14. ^{1}H, ^{13}C-NMR spectra of product **18**

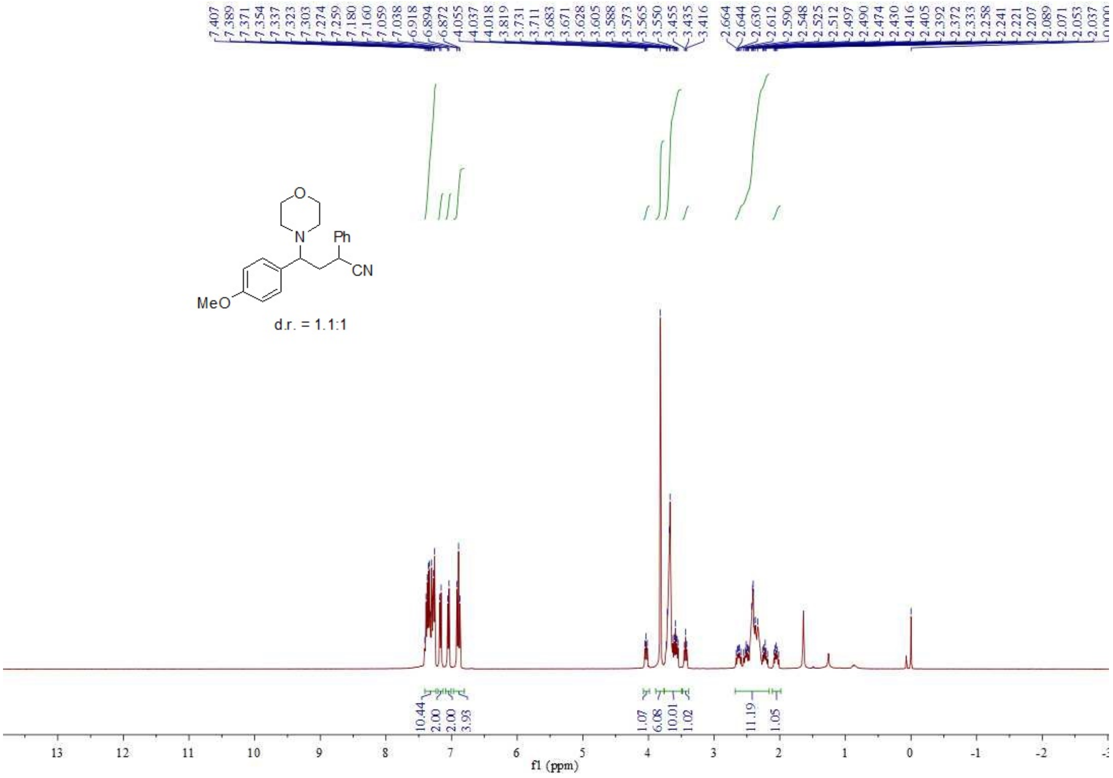

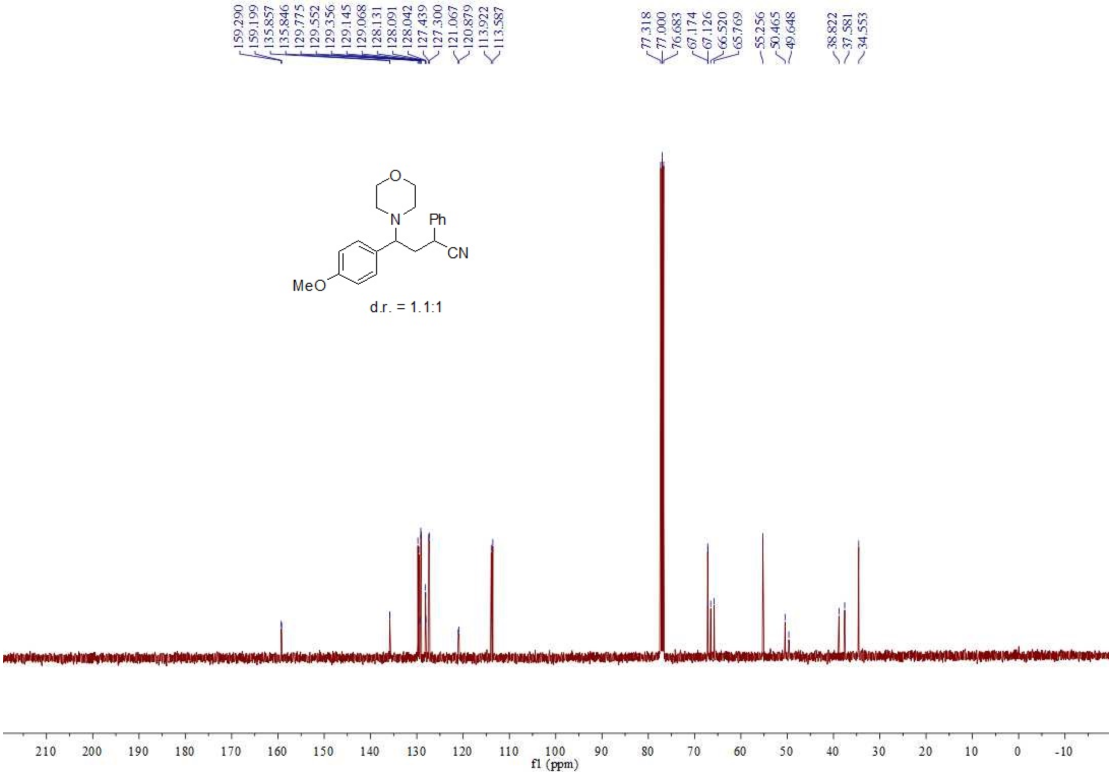

Supplementary Figure 15. ^{1}H, ^{13}C-NMR spectra of product **19**

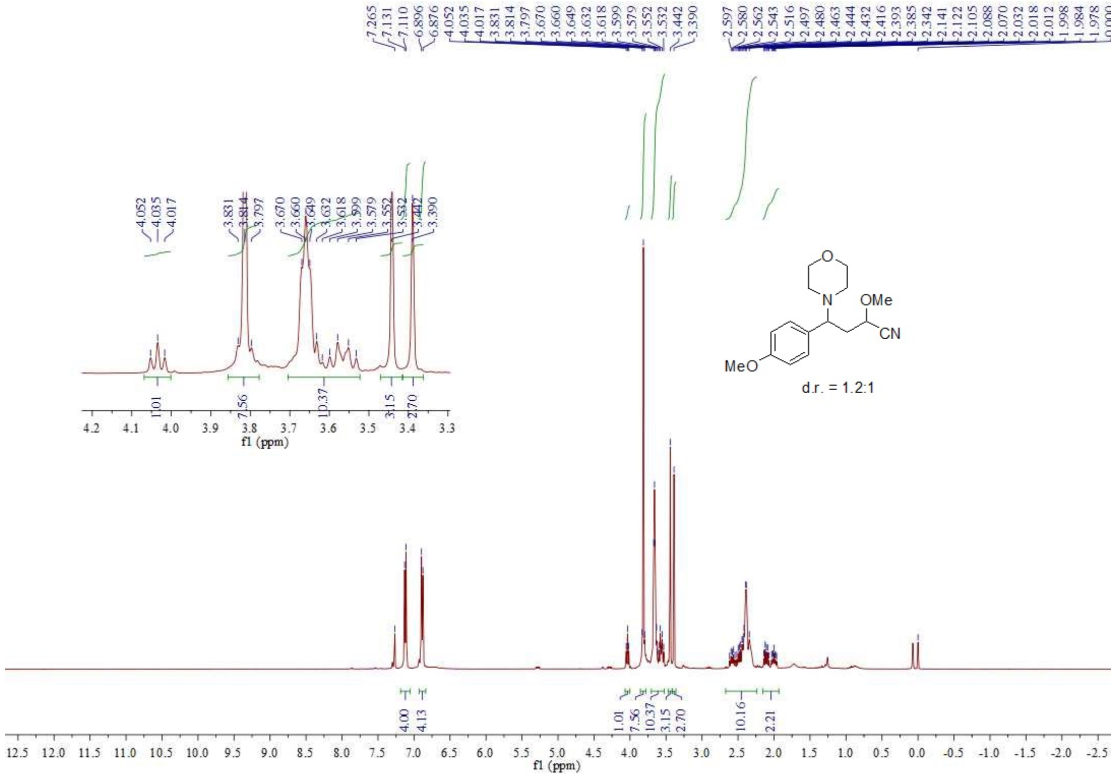

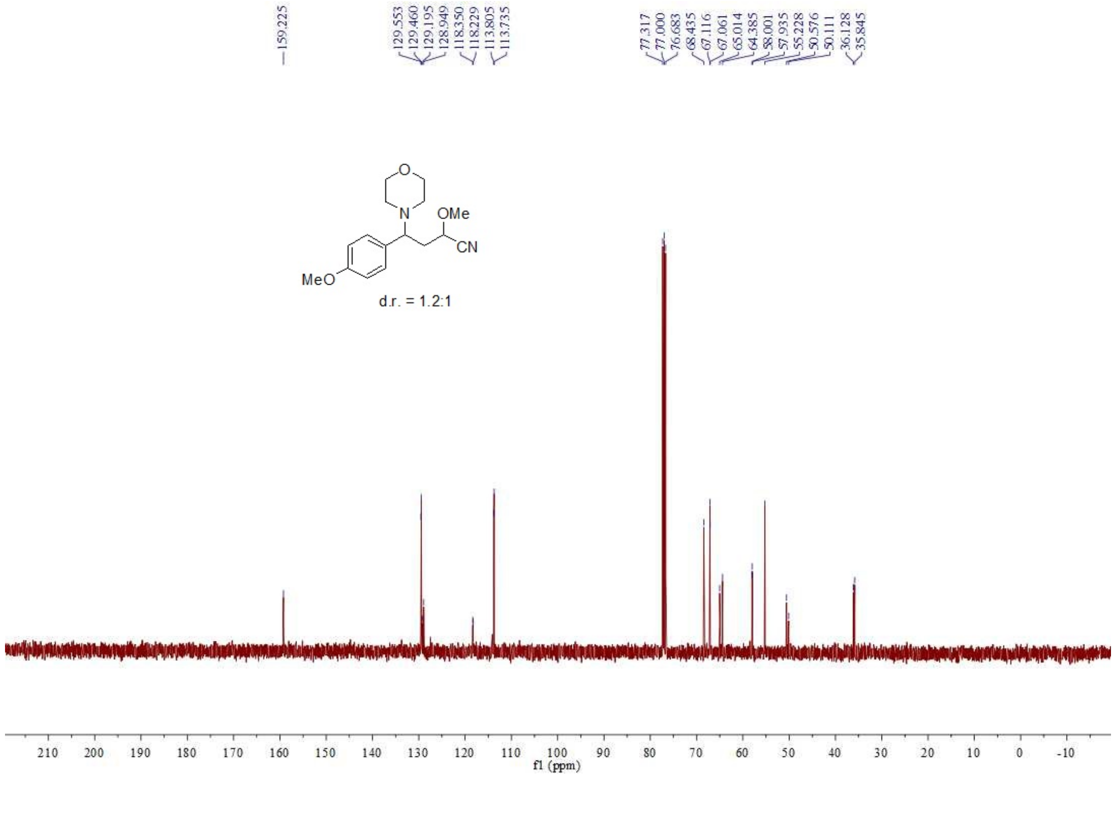

Supplementary Figure 16. ^{1}H, ^{13}C-NMR spectra of product **20**

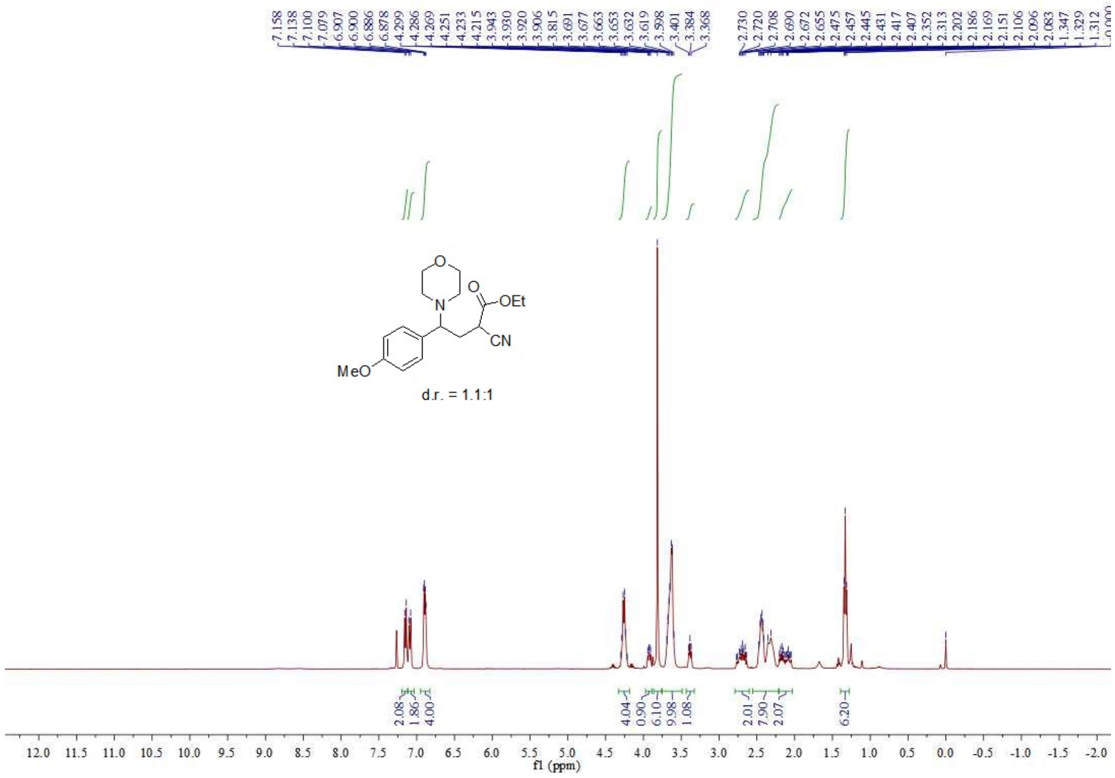

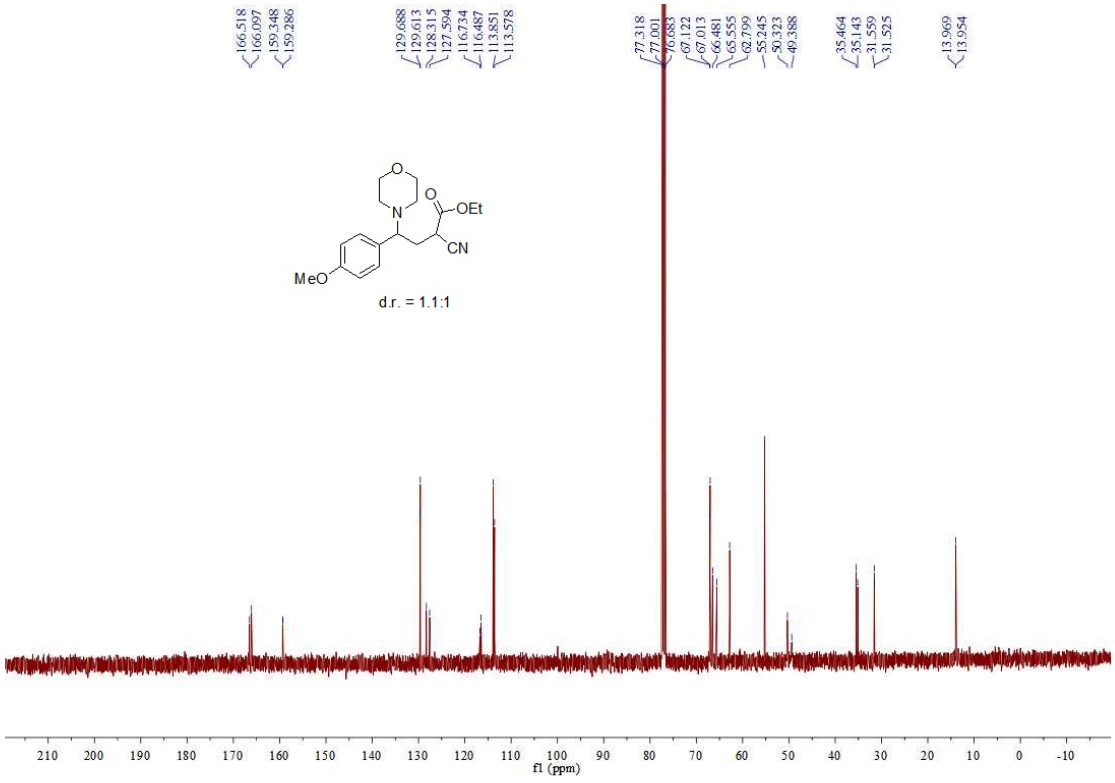

Supplementary Figure 17. ^{1}H, ^{13}C-NMR spectra of product **21**

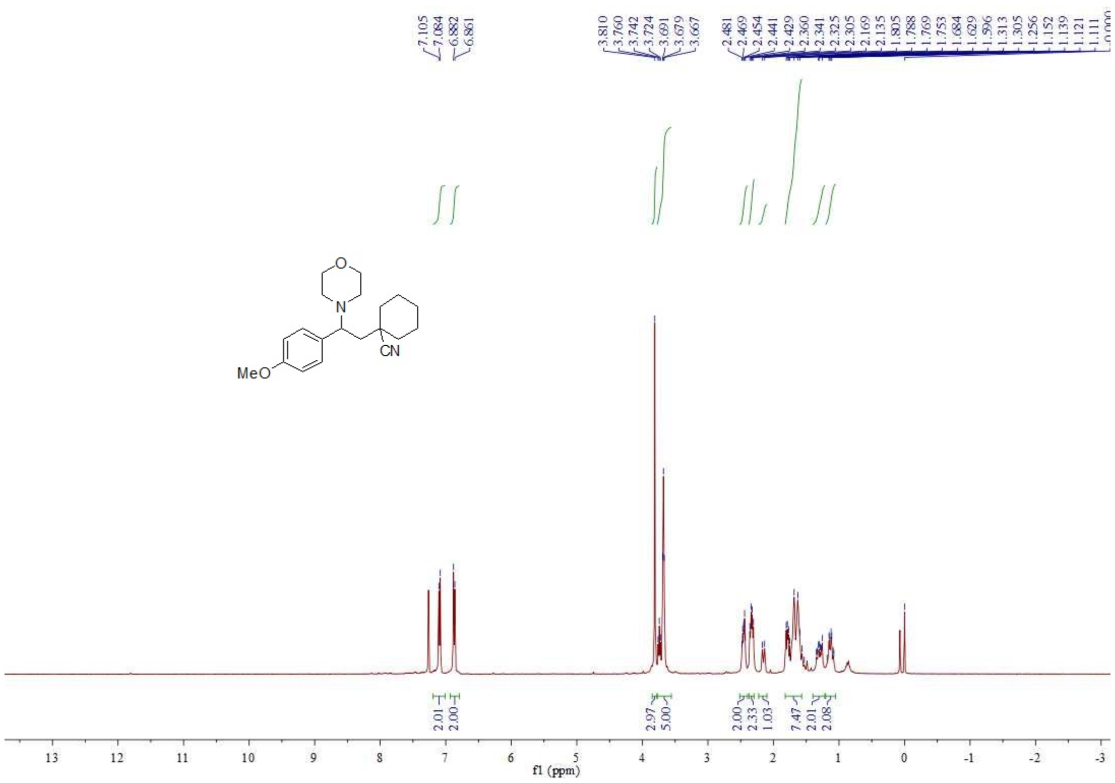

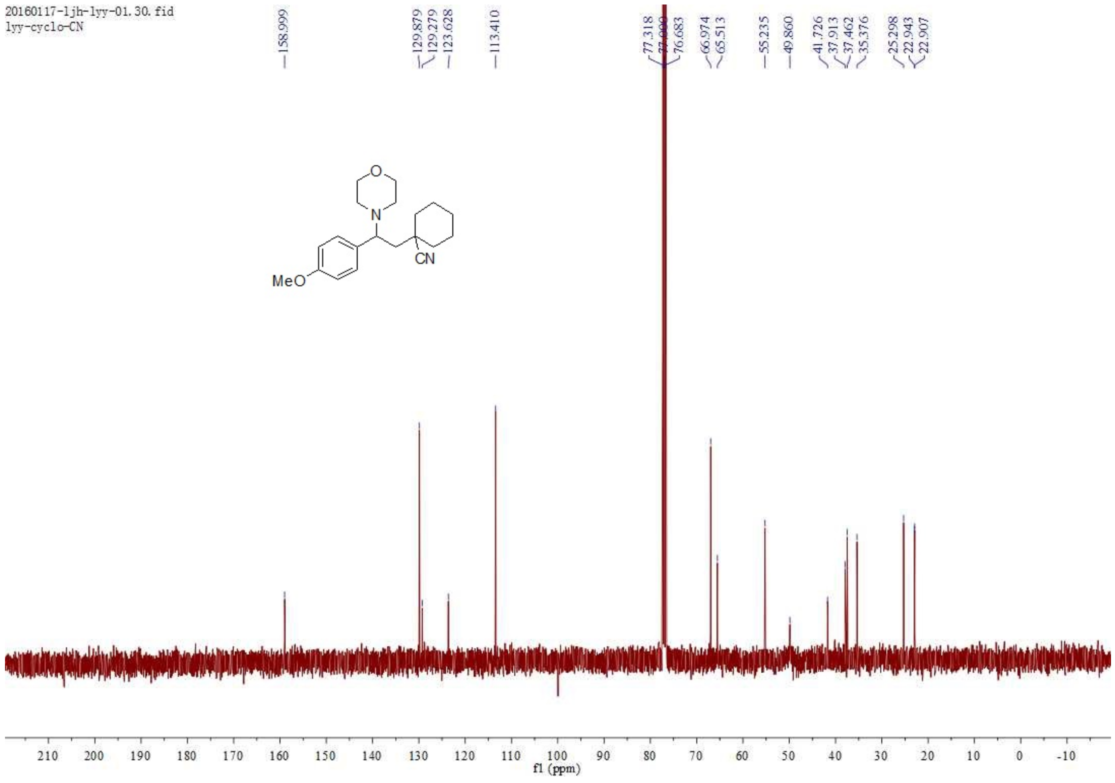

Supplementary Figure 18. ^{1}H, ^{13}C-NMR spectra of product **22**

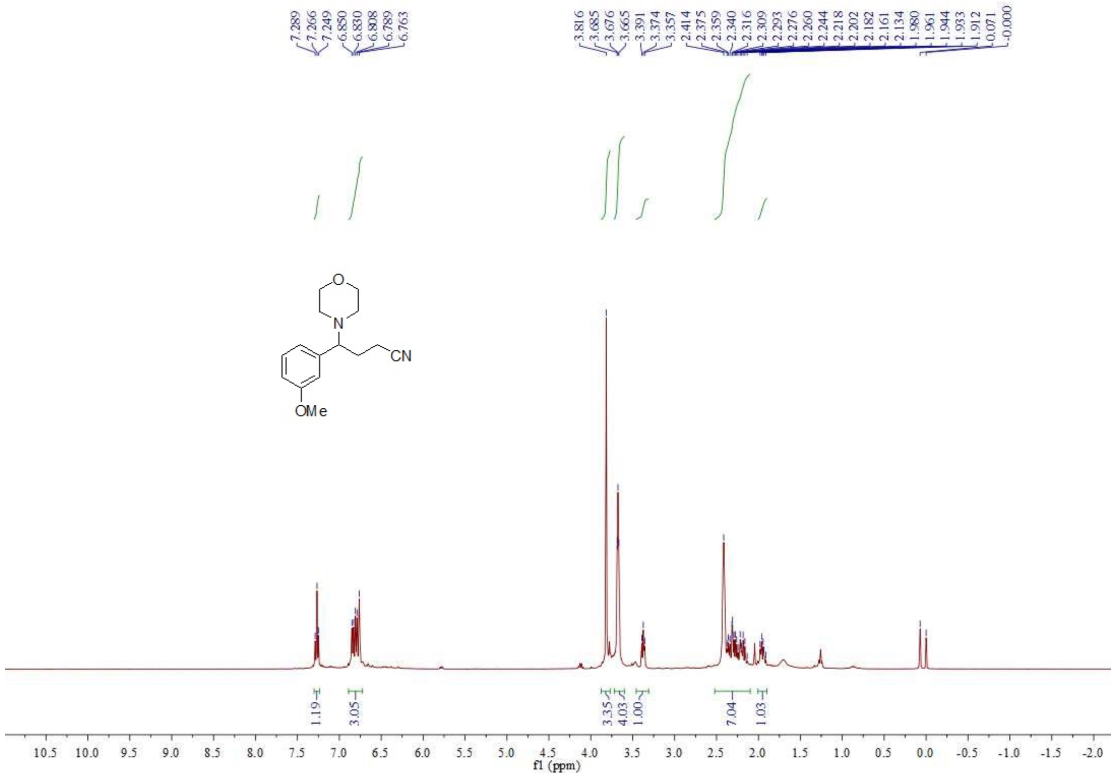

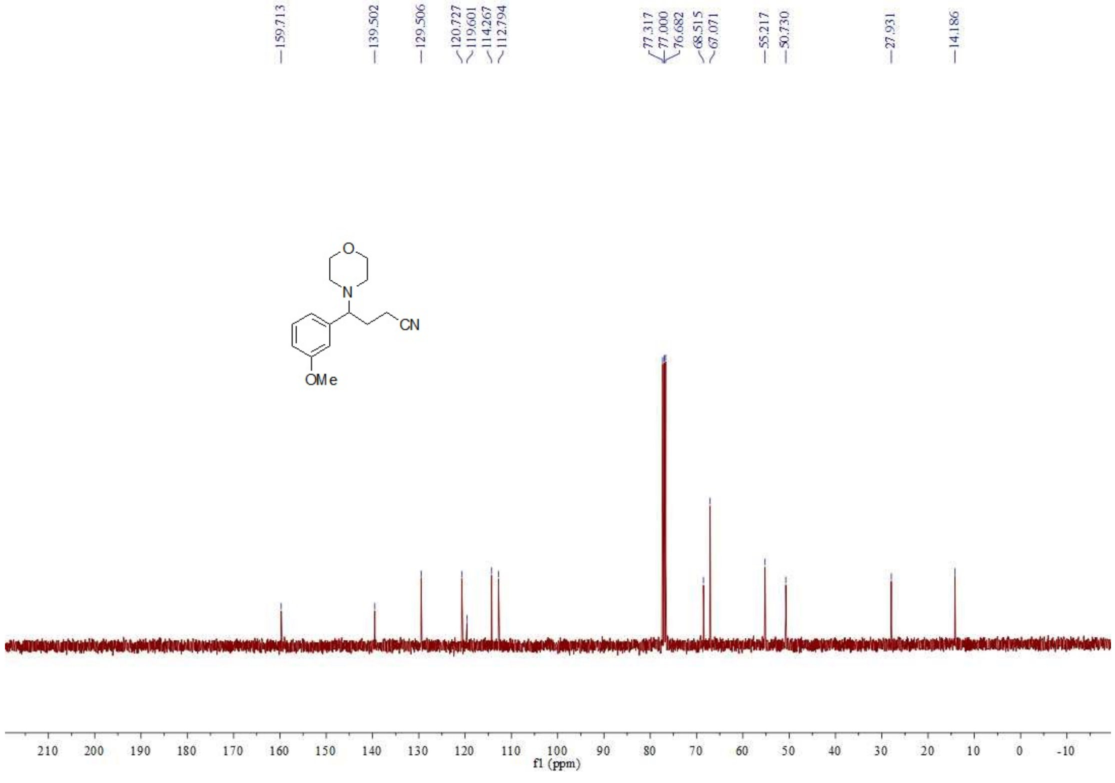

Supplementary Figure 19. ^{1}H, ^{13}C-NMR spectra of product **23**

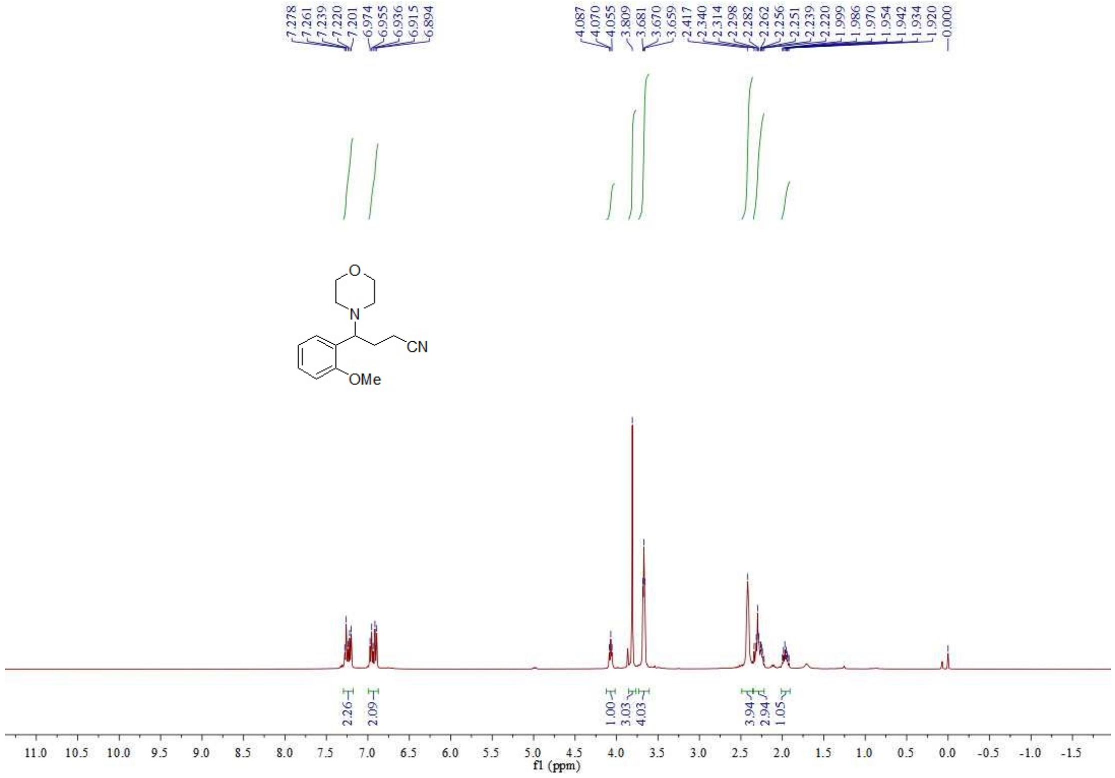

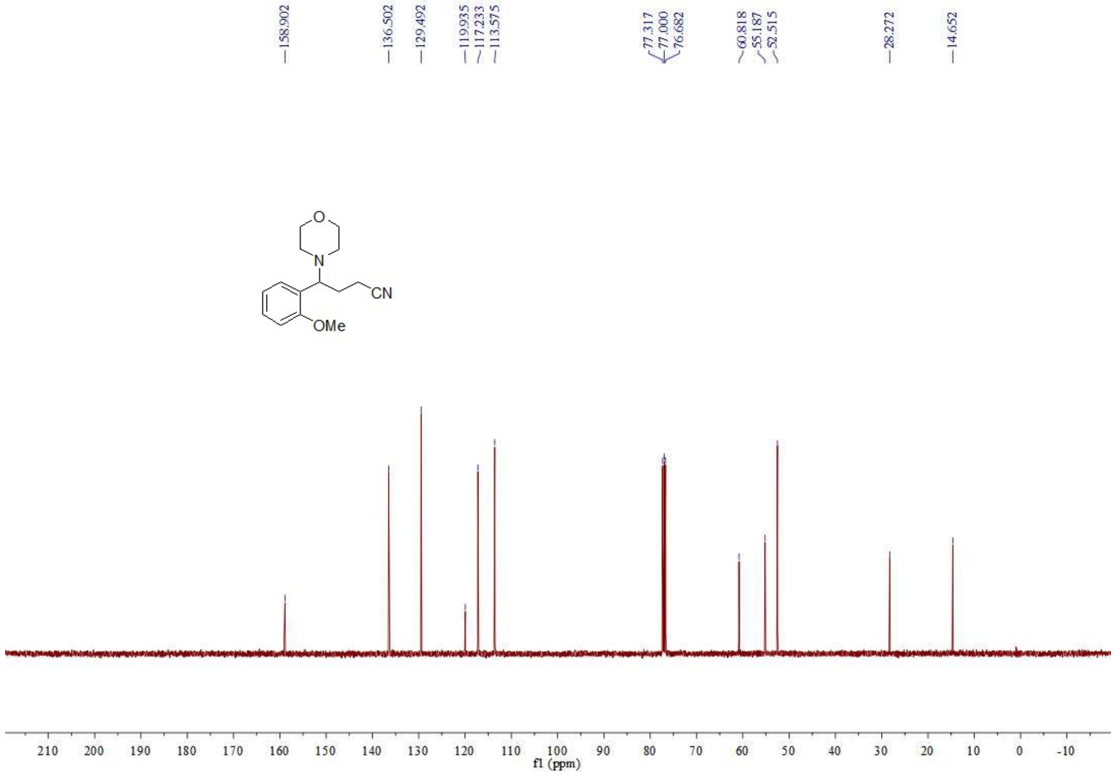

Supplementary Figure 20. ^{1}H, ^{13}C-NMR spectra of product **24**

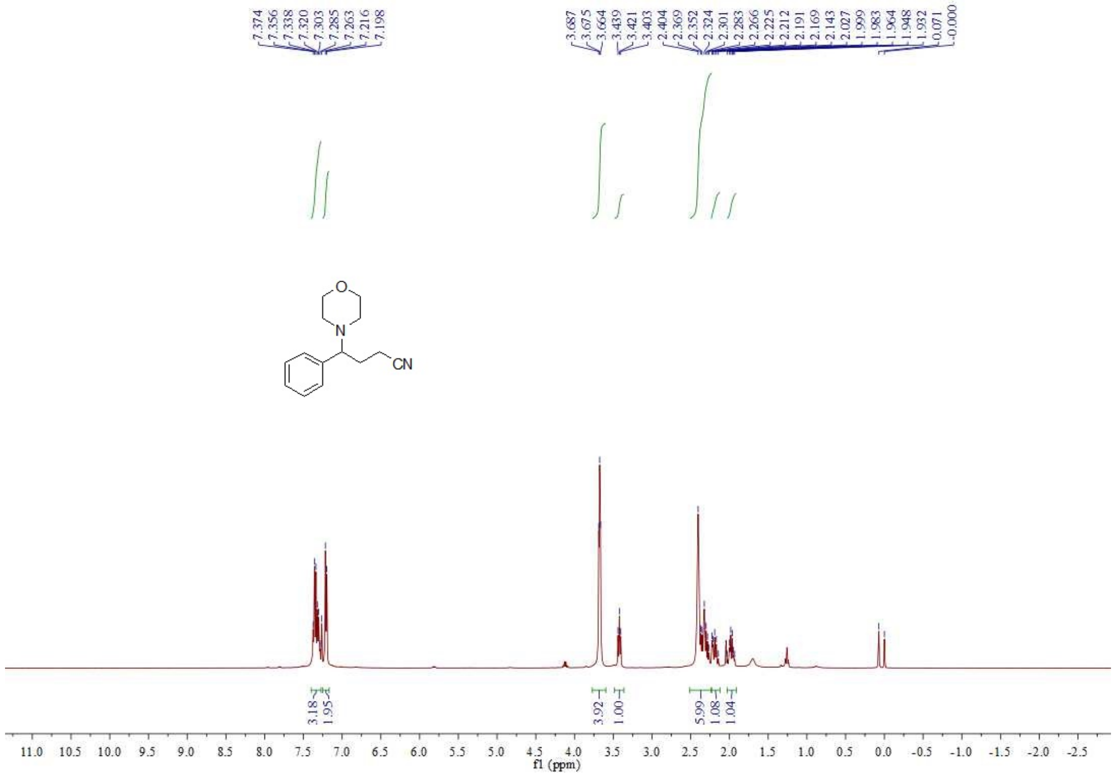

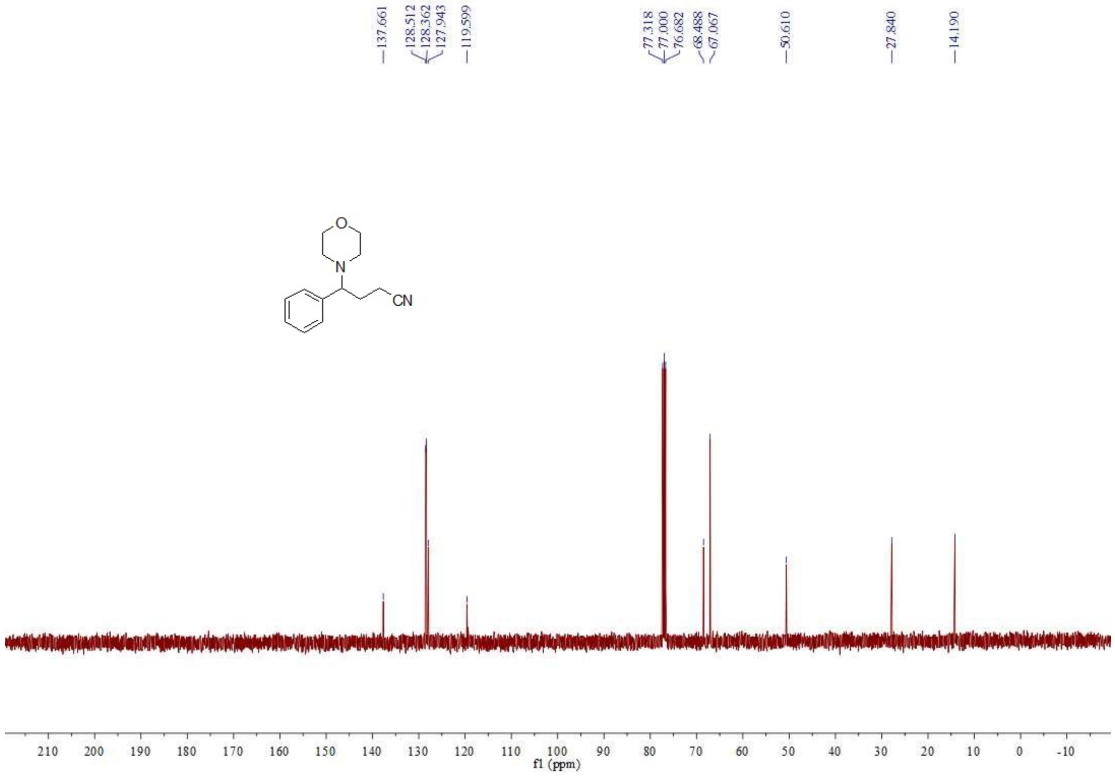

Supplementary Figure 21. ^{1}H, ^{13}C-NMR spectra of product **25**

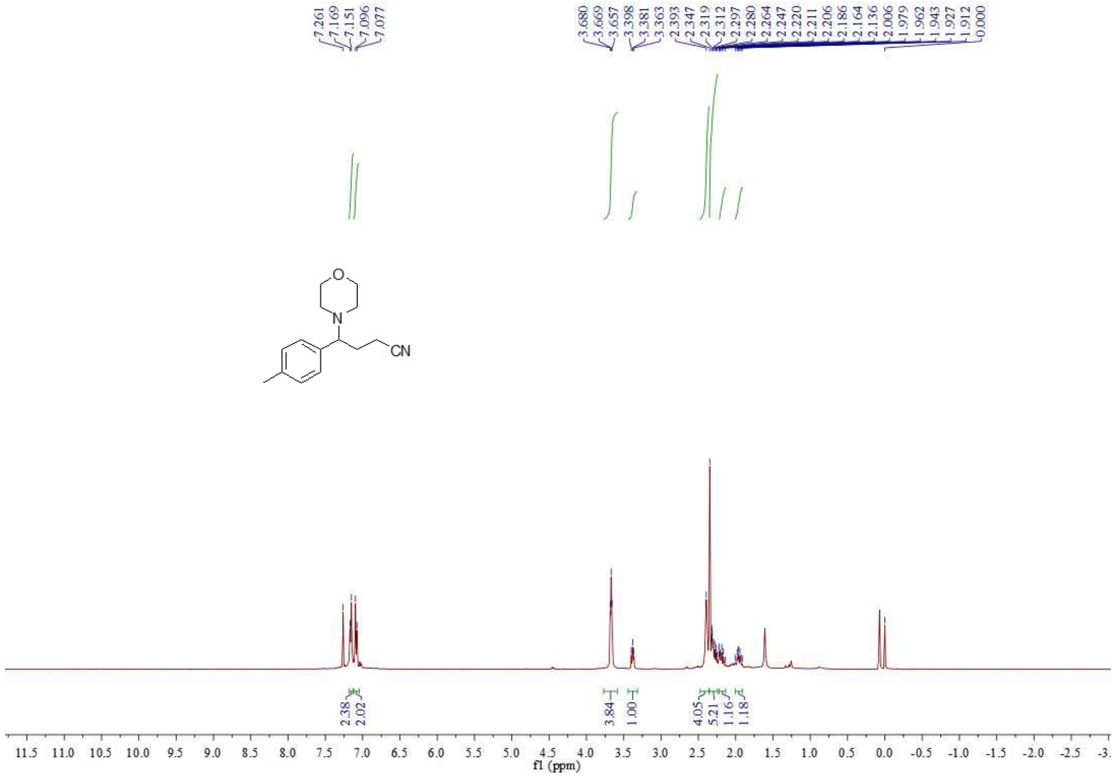

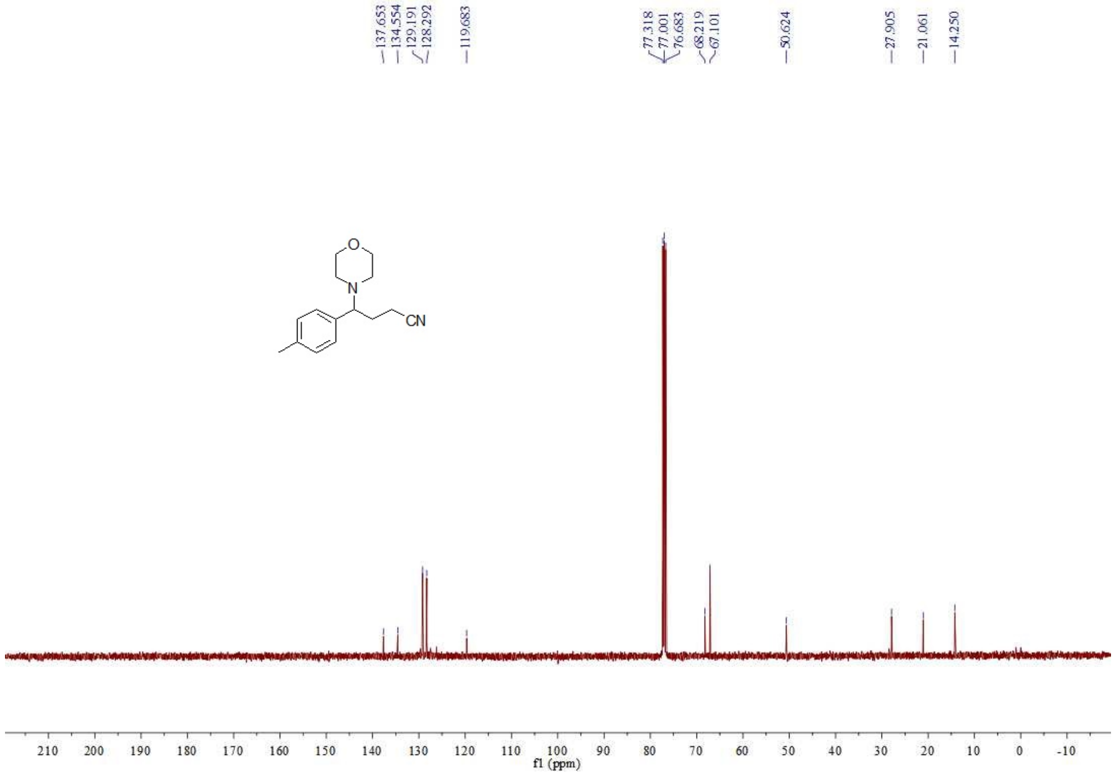

Supplementary Figure 22. ^{1}H, ^{13}C-NMR spectra of product **26**

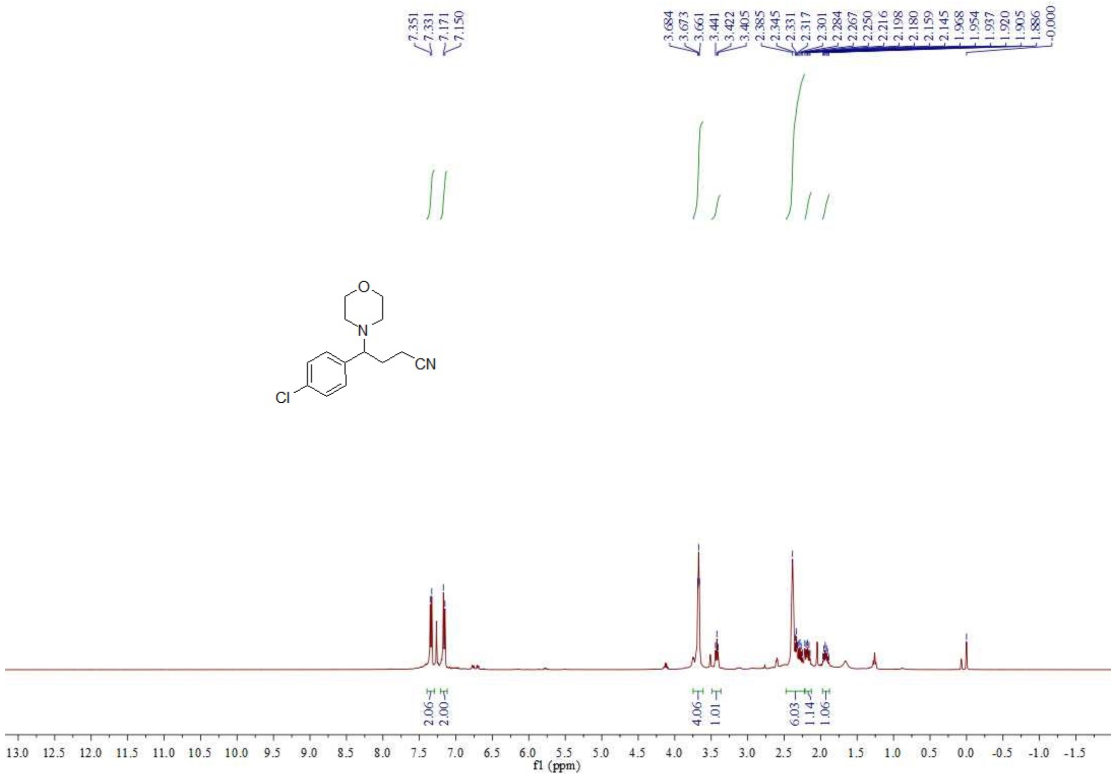

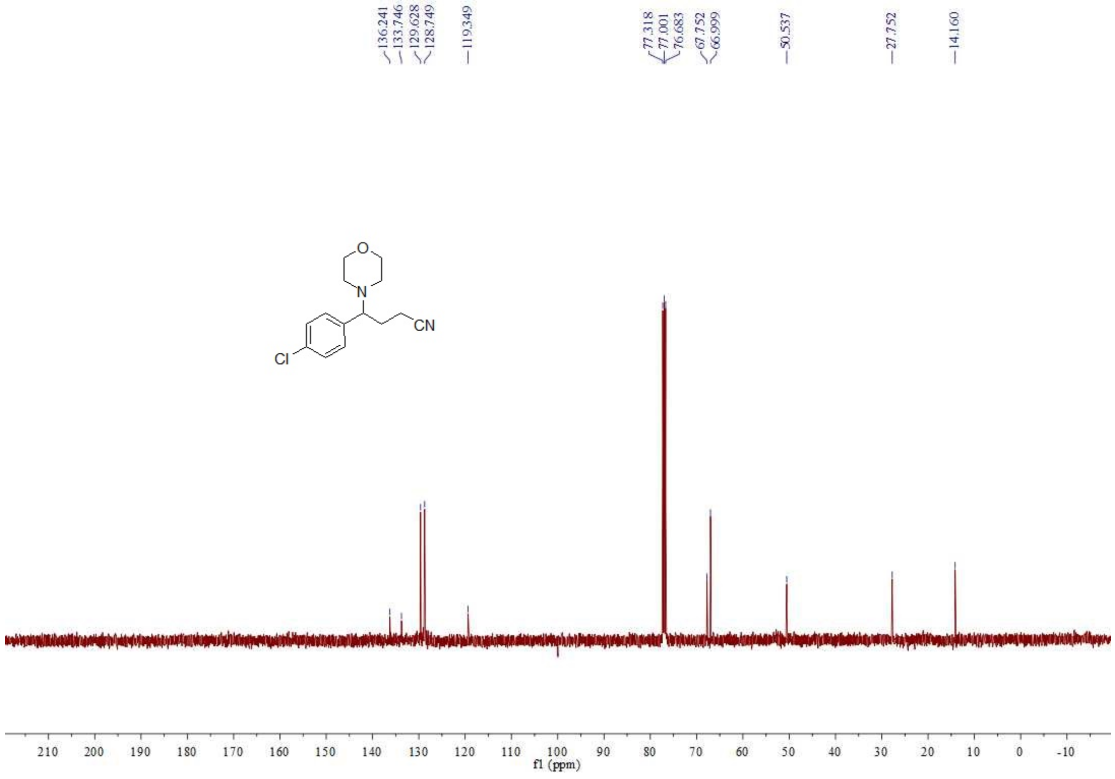

Supplementary Figure 23. ^{1}H, ^{13}C-NMR spectra of product **28**

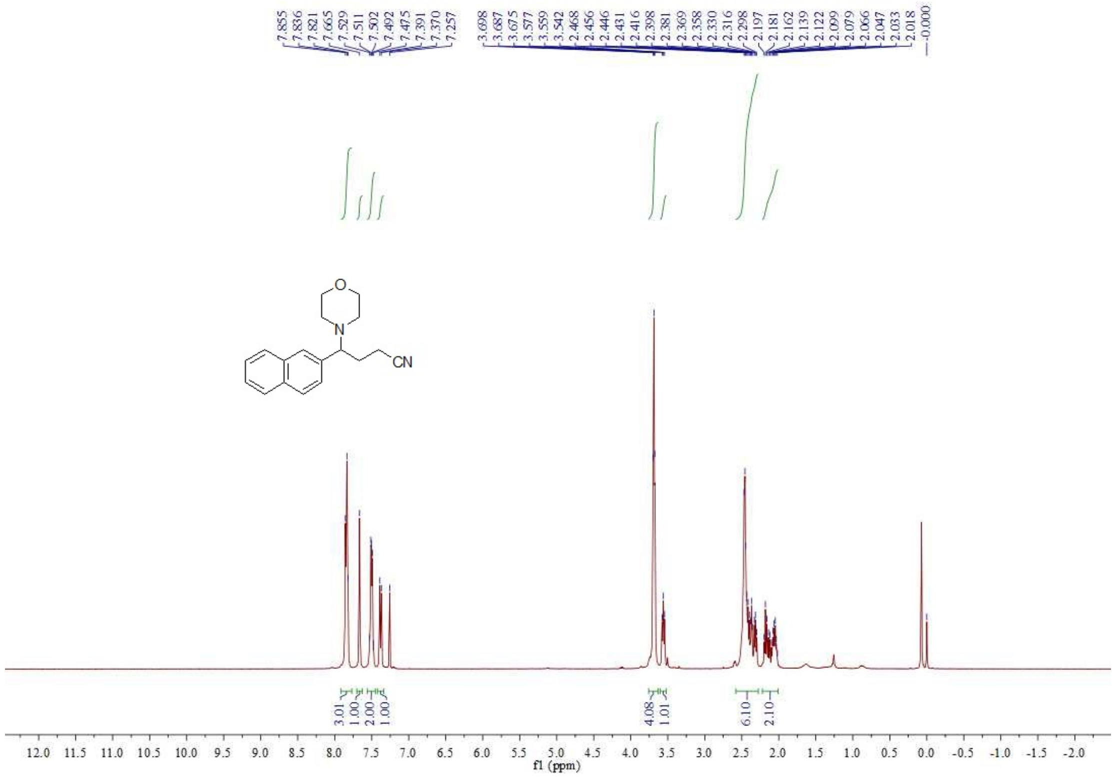

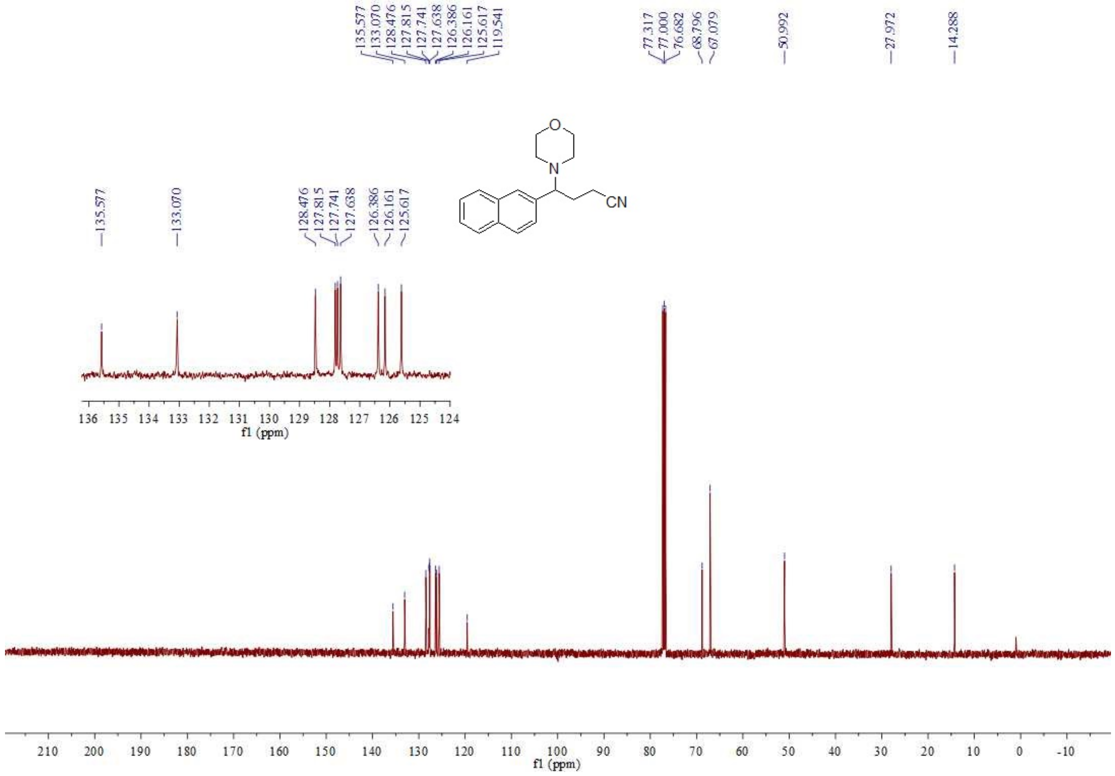

Supplementary Figure 24. ^{1}H, ^{13}C-NMR spectra of product **29**

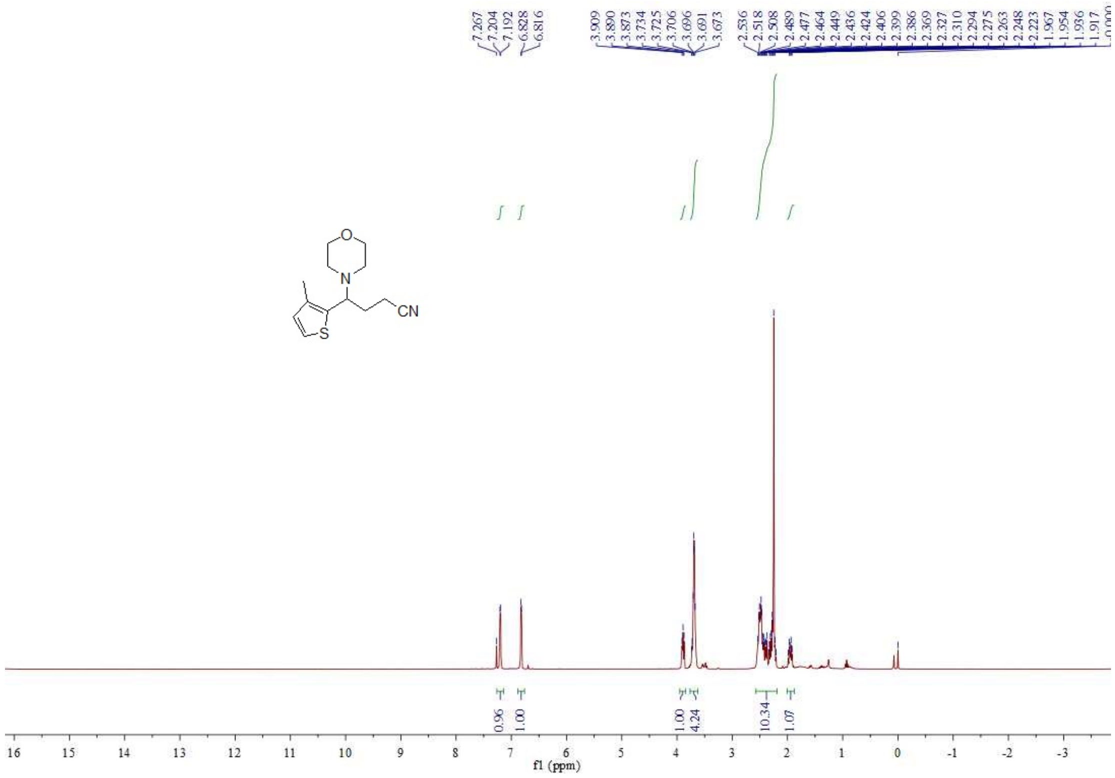

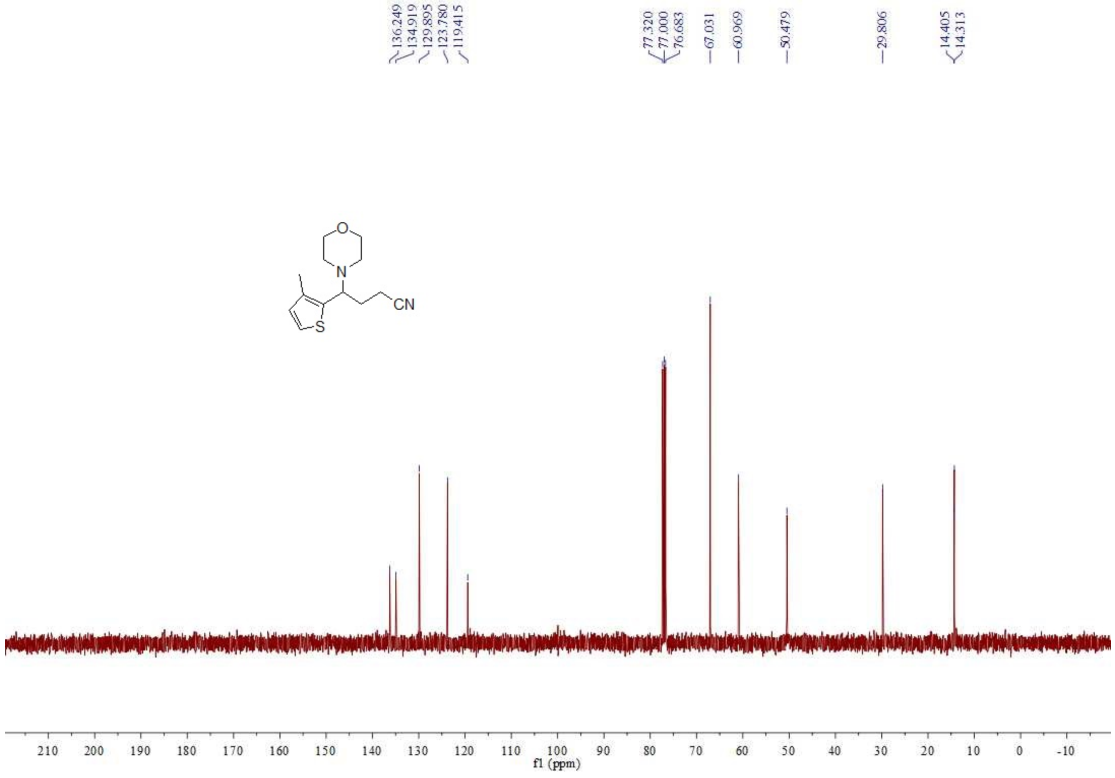

Supplementary Figure 25. ^{1}H, ^{13}C-NMR spectra of product **31**

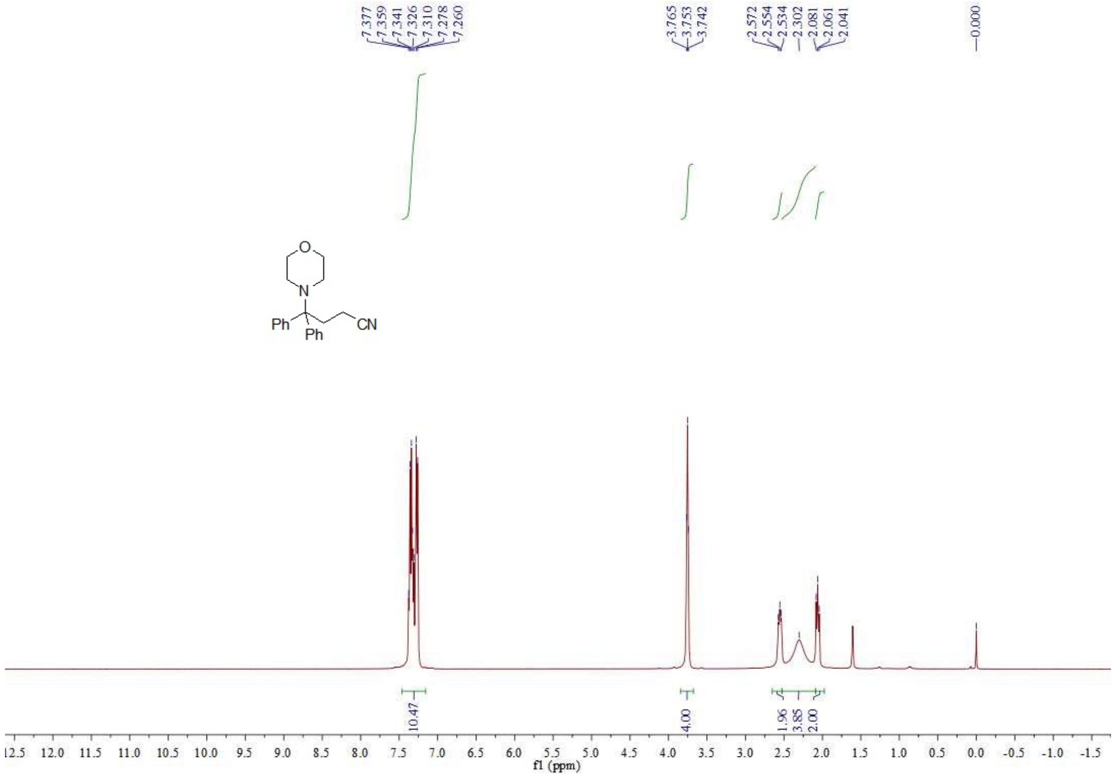

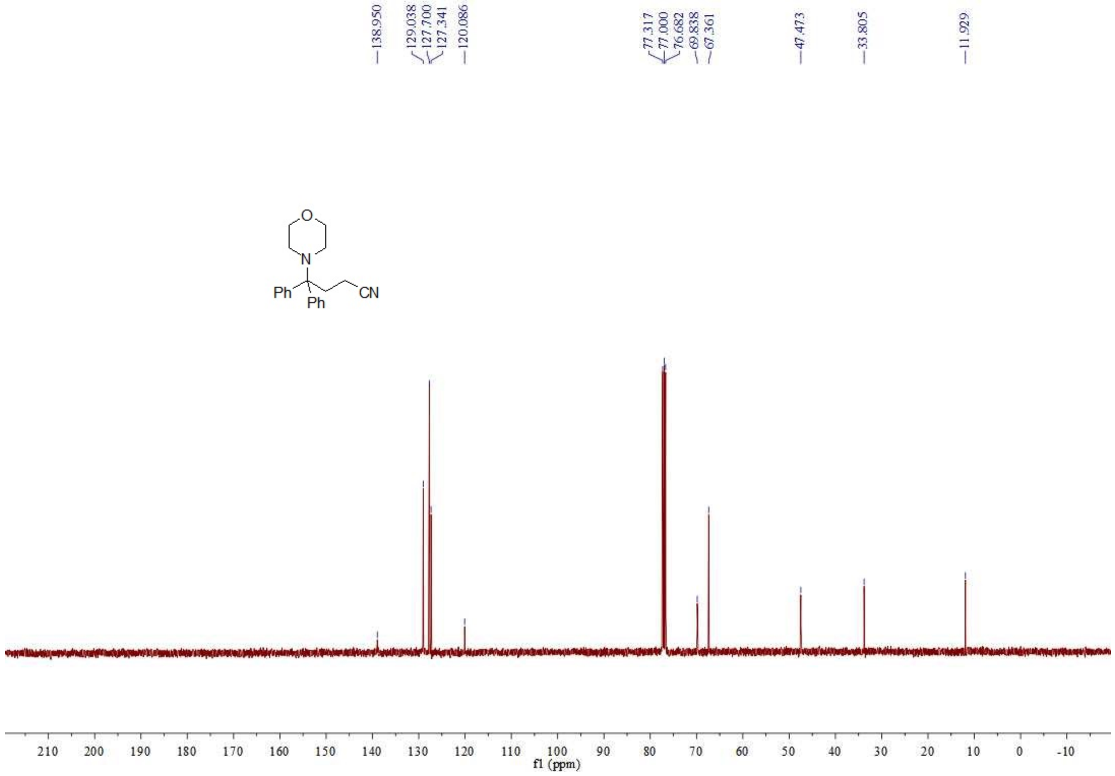

Supplementary Figure 26. ^{1}H, ^{13}C-NMR spectra of product **32**

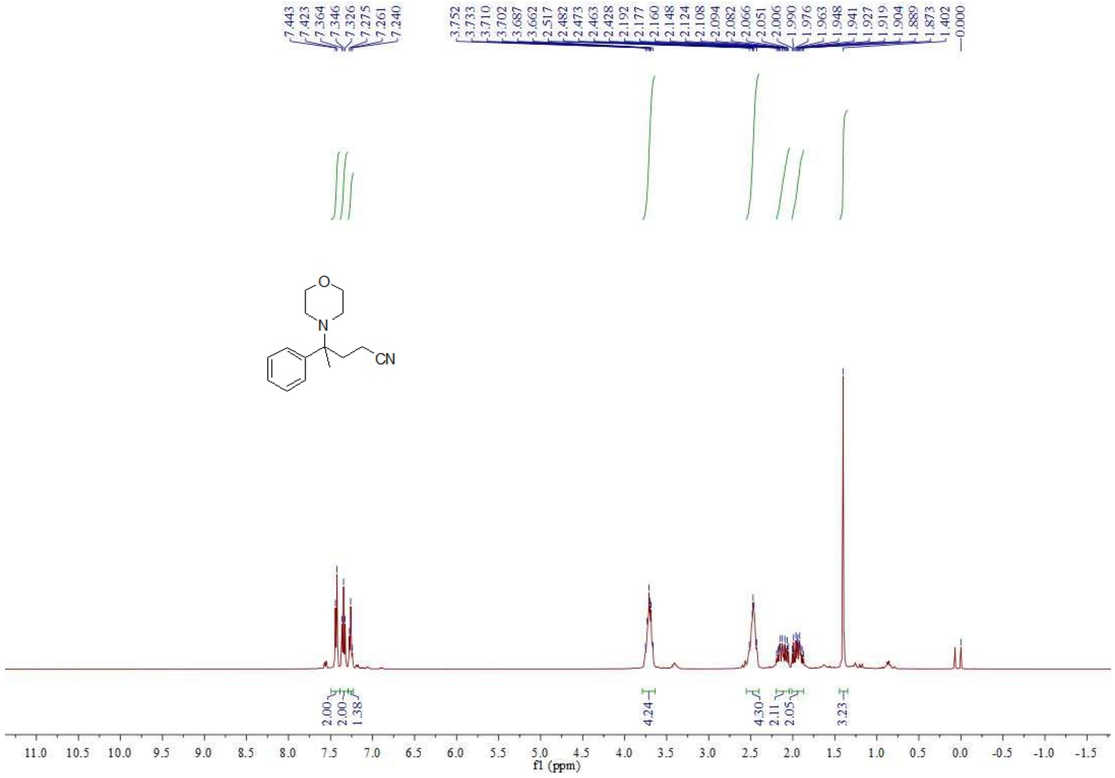

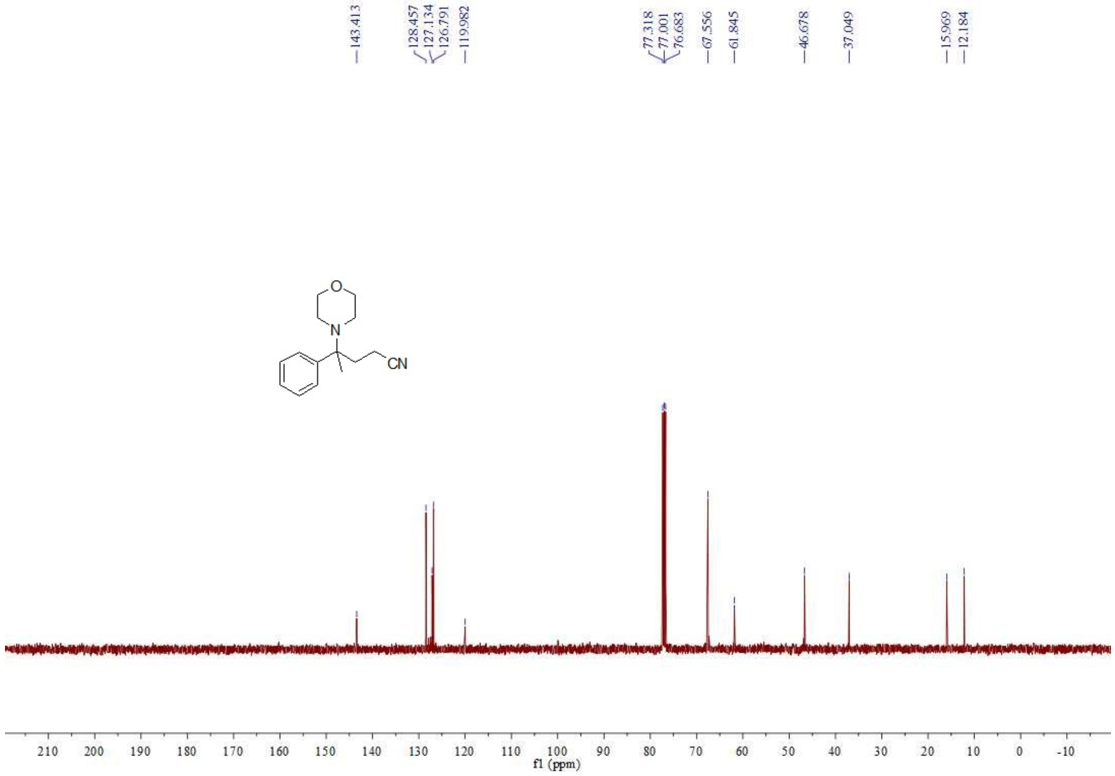

Supplementary Figure 27. ^{1}H, ^{13}C-NMR spectra of product **33**

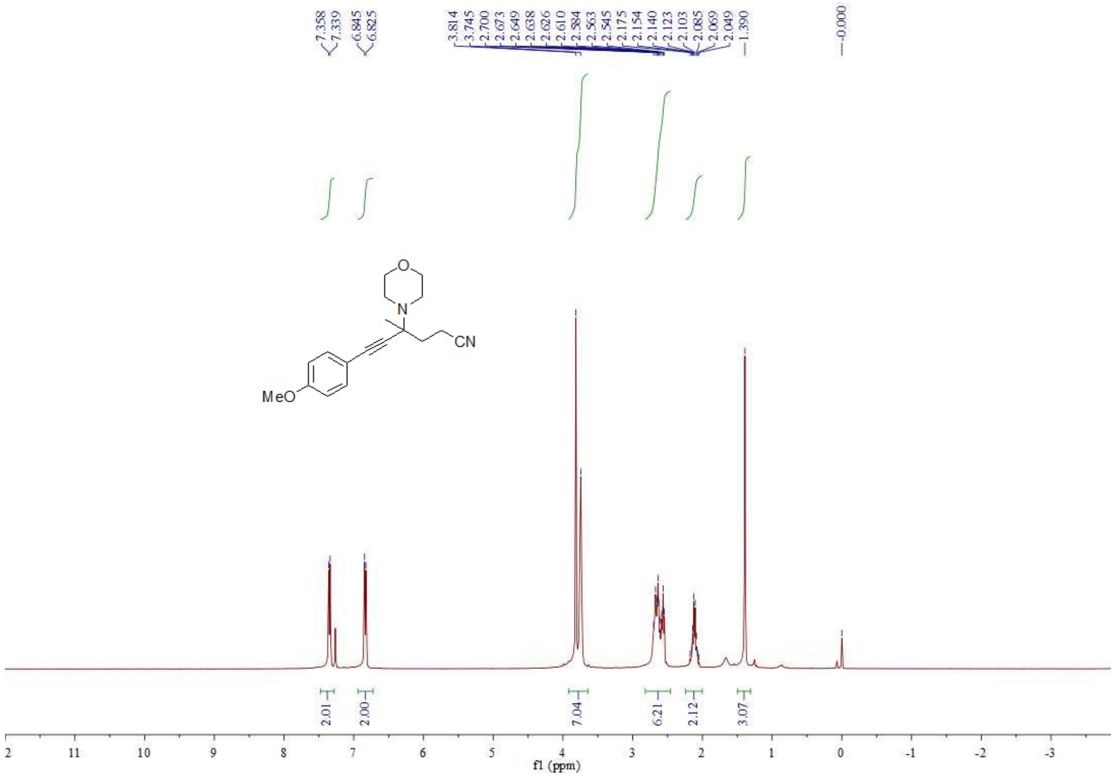

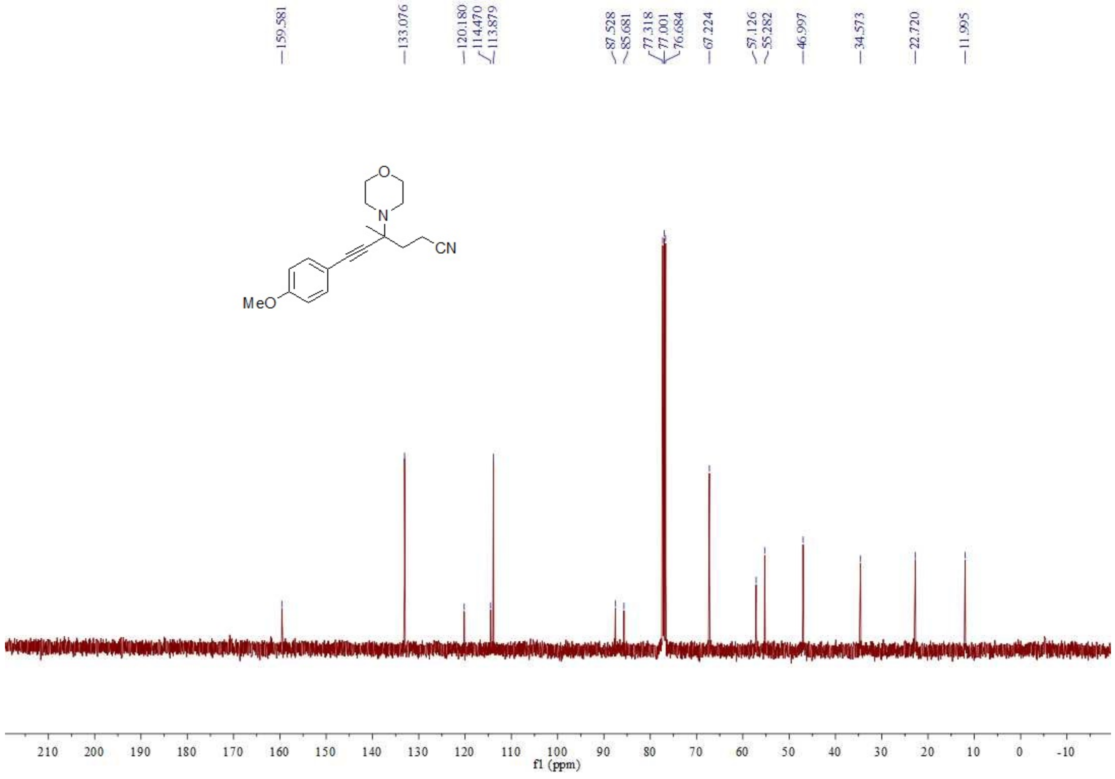

Supplementary Figure 28. ^{1}H, ^{13}C-NMR spectra of product **34**

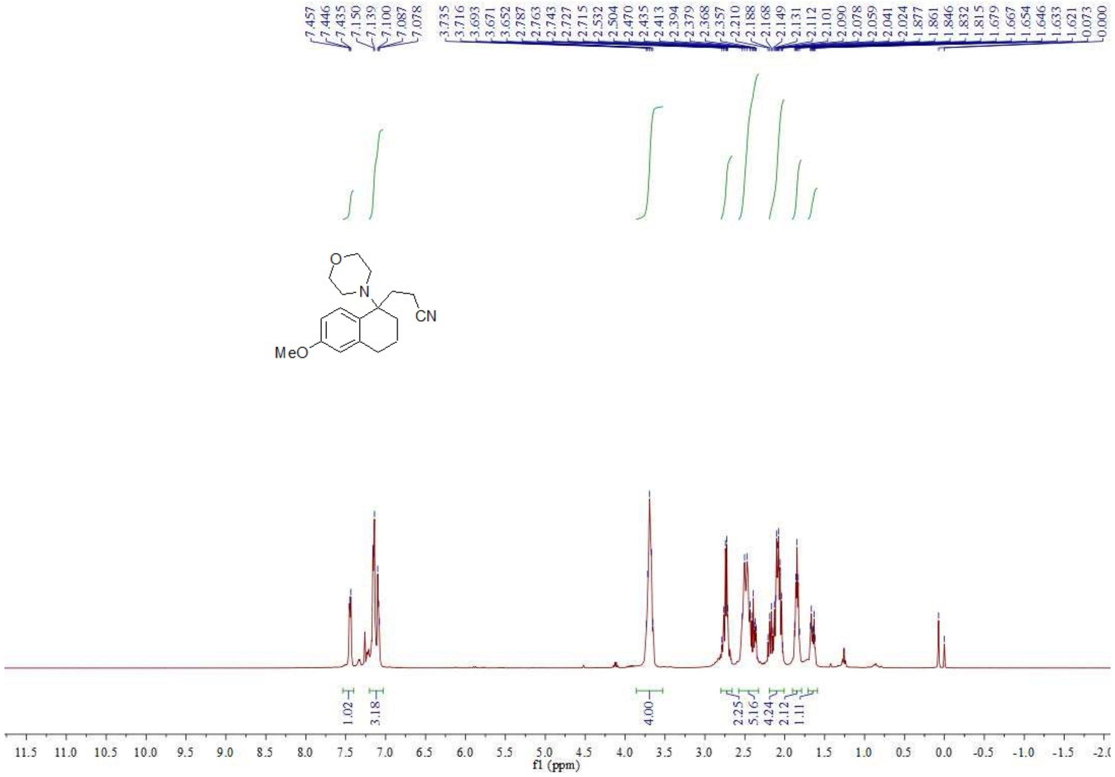

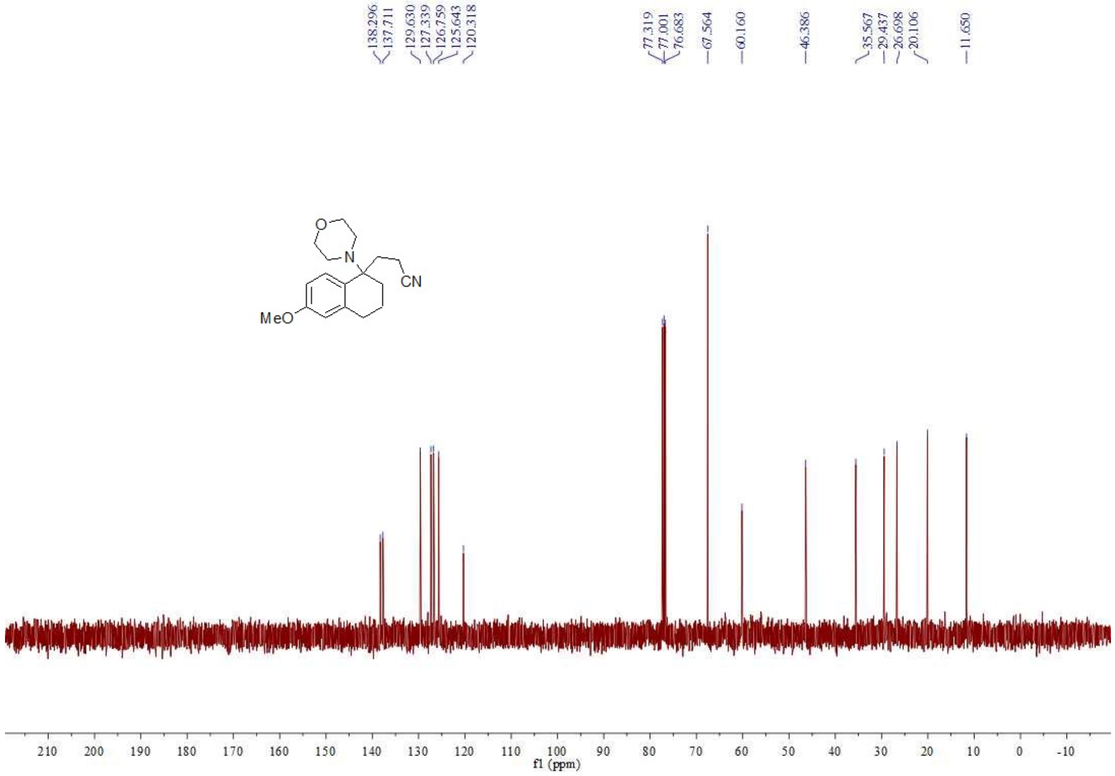

Supplementary Figure 29. ^{1}H, ^{13}C-NMR spectra of product **35**

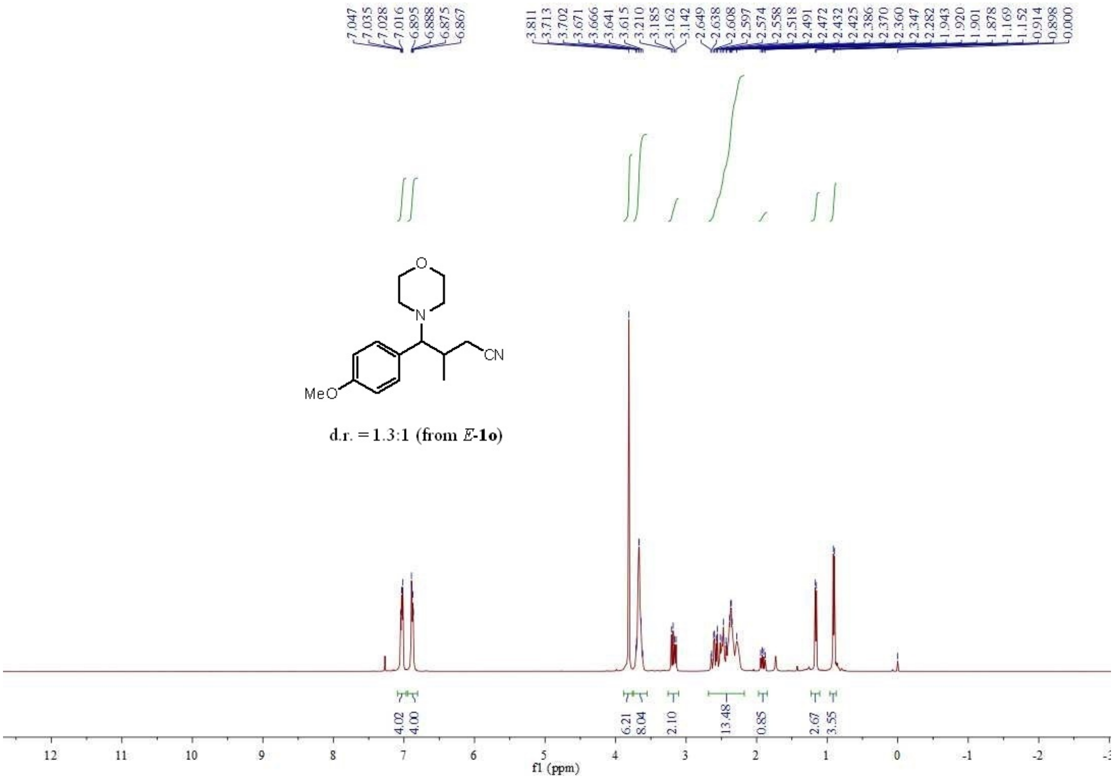

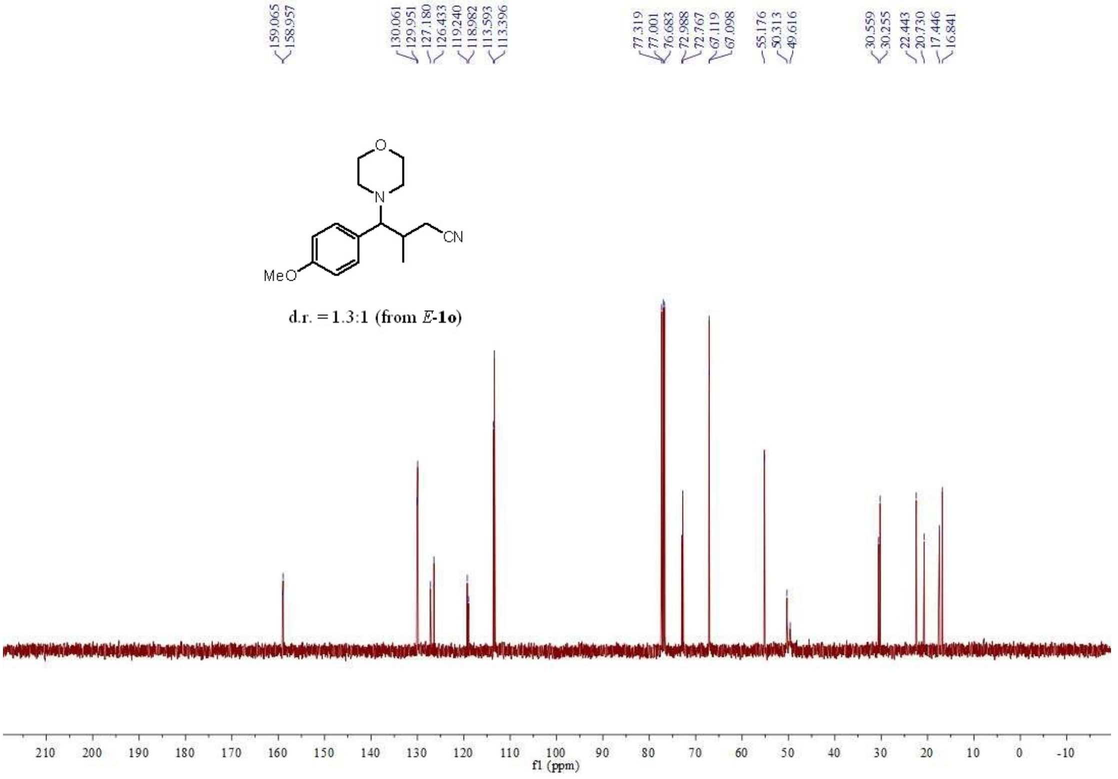

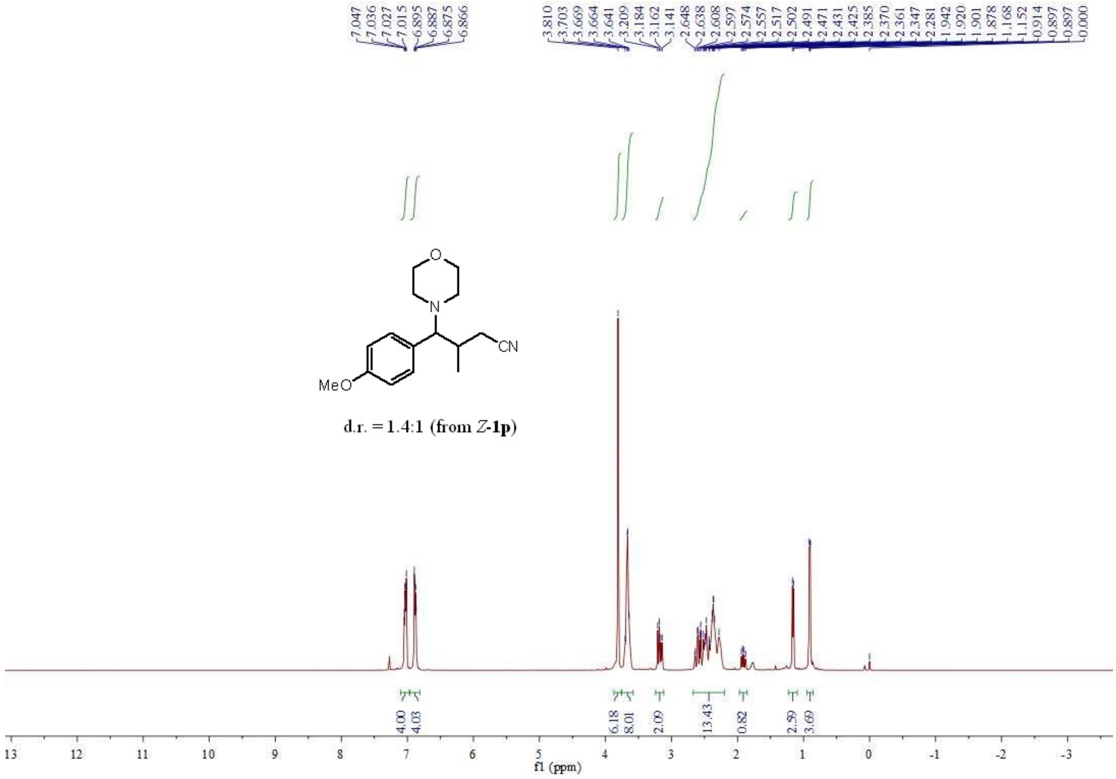

d.r. = 1.4:1 (from *Z*-**1p**)

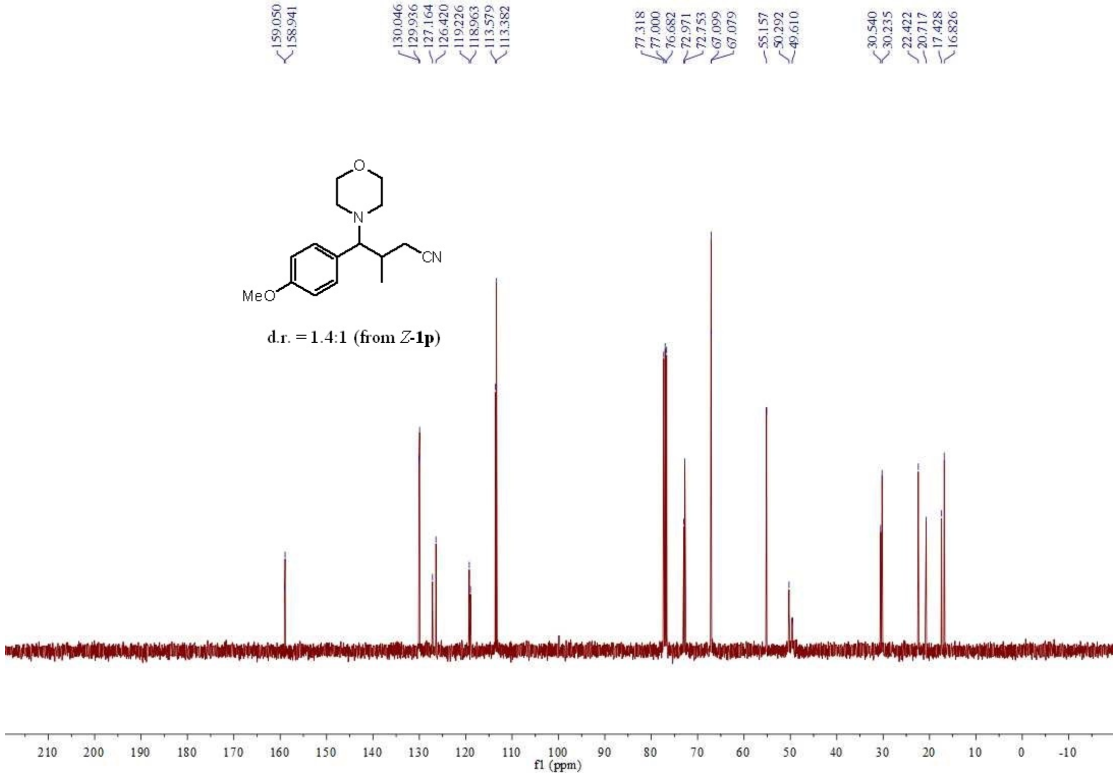

d.r. = 1.4:1 (from *Z*-**1p**)

Supplementary Figure 30. ^{1}H, ^{13}C-NMR spectra of product **36**

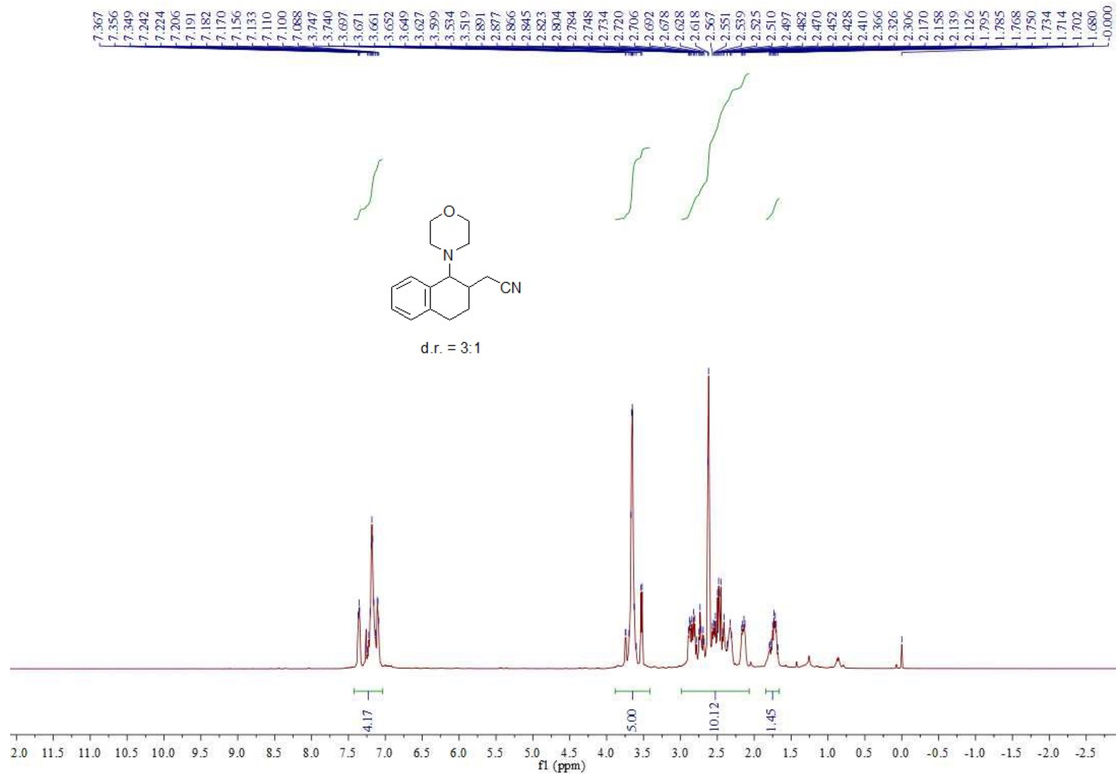

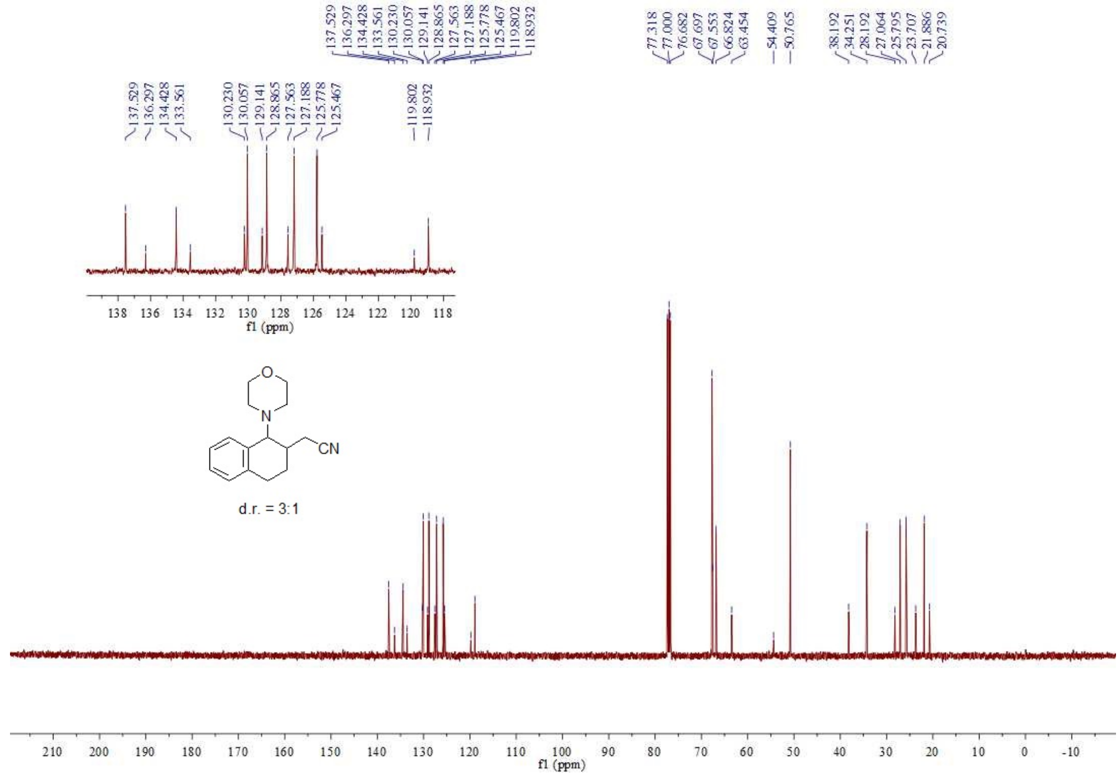

Supplementary Figure 31. ^{1}H, ^{13}C-NMR spectra of product **37**

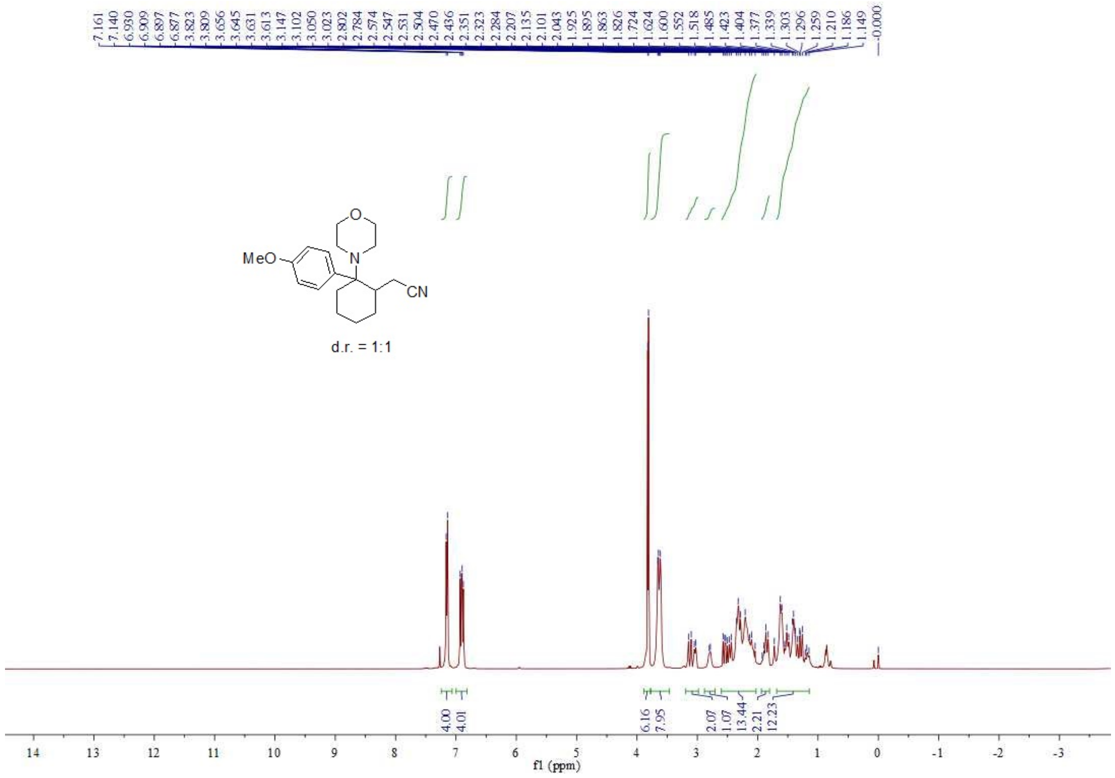

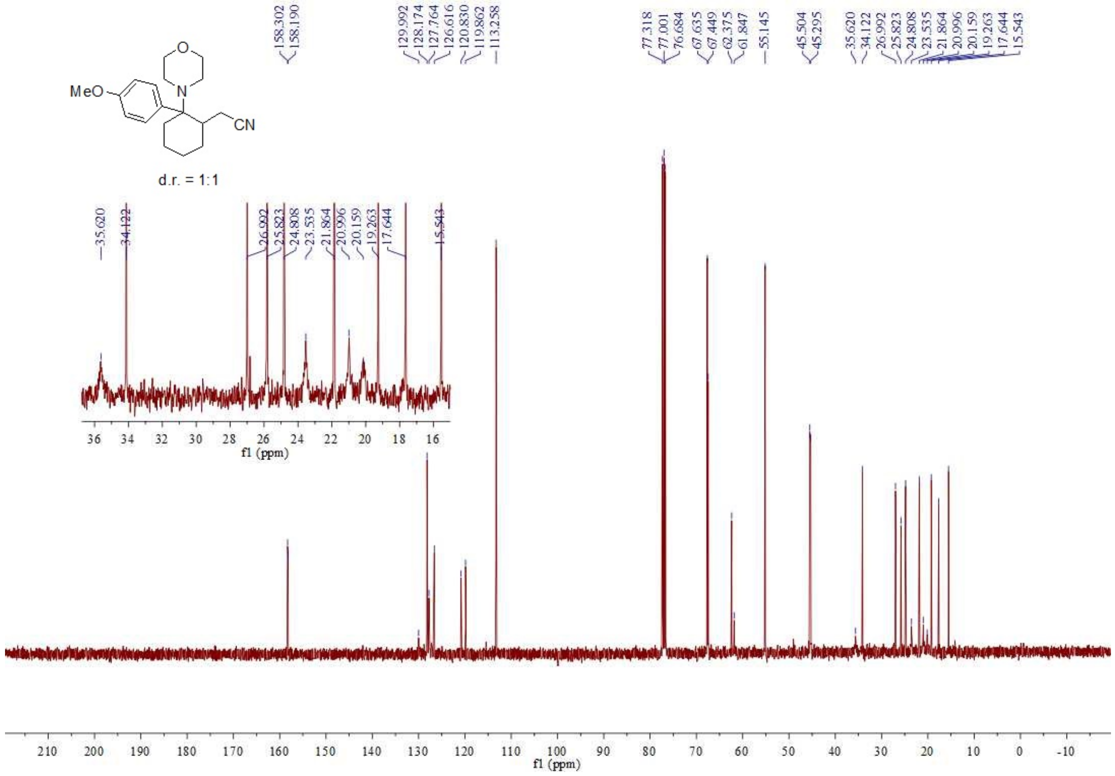

Supplementary Figure 32. ^{1}H, ^{13}C-NMR spectra of product **38**

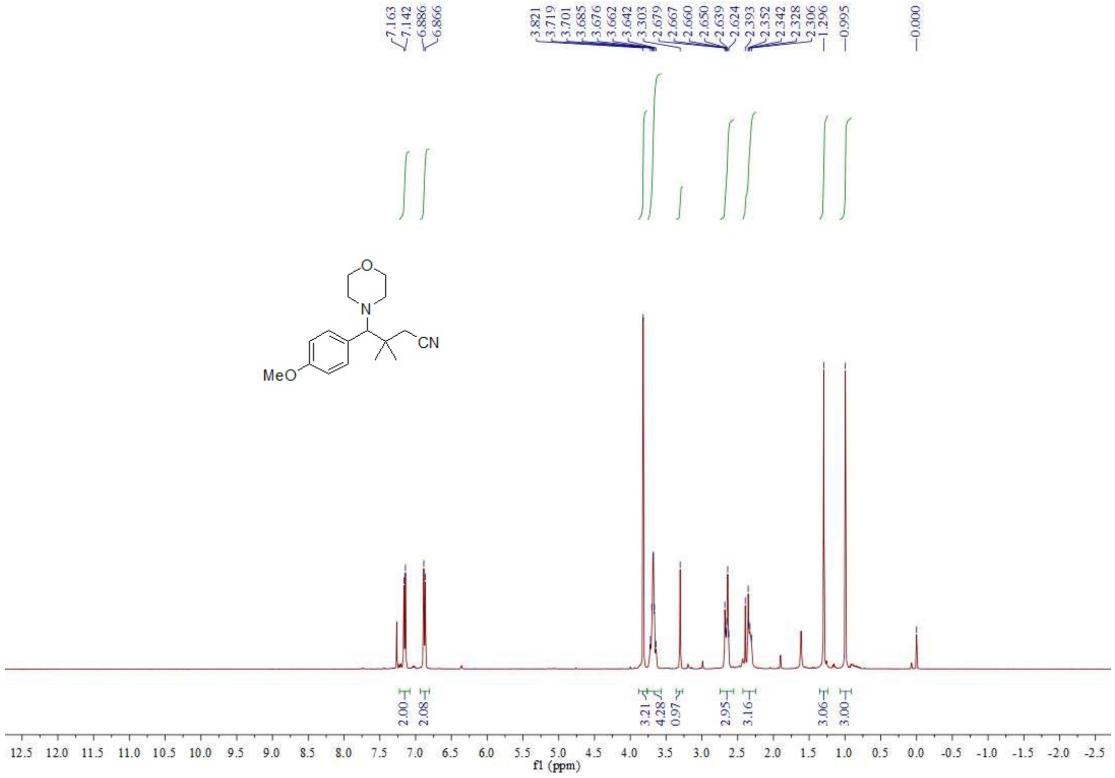

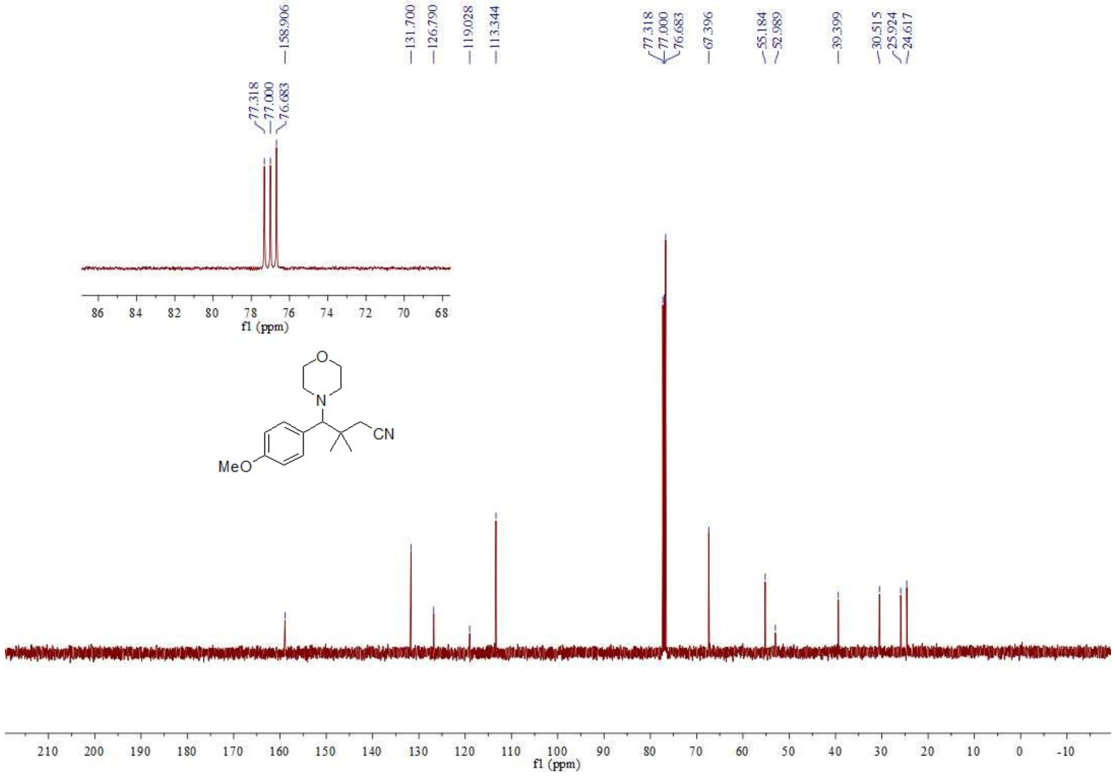

Supplementary Figure 33. ^{1}H, ^{13}C-NMR spectra of product **39**

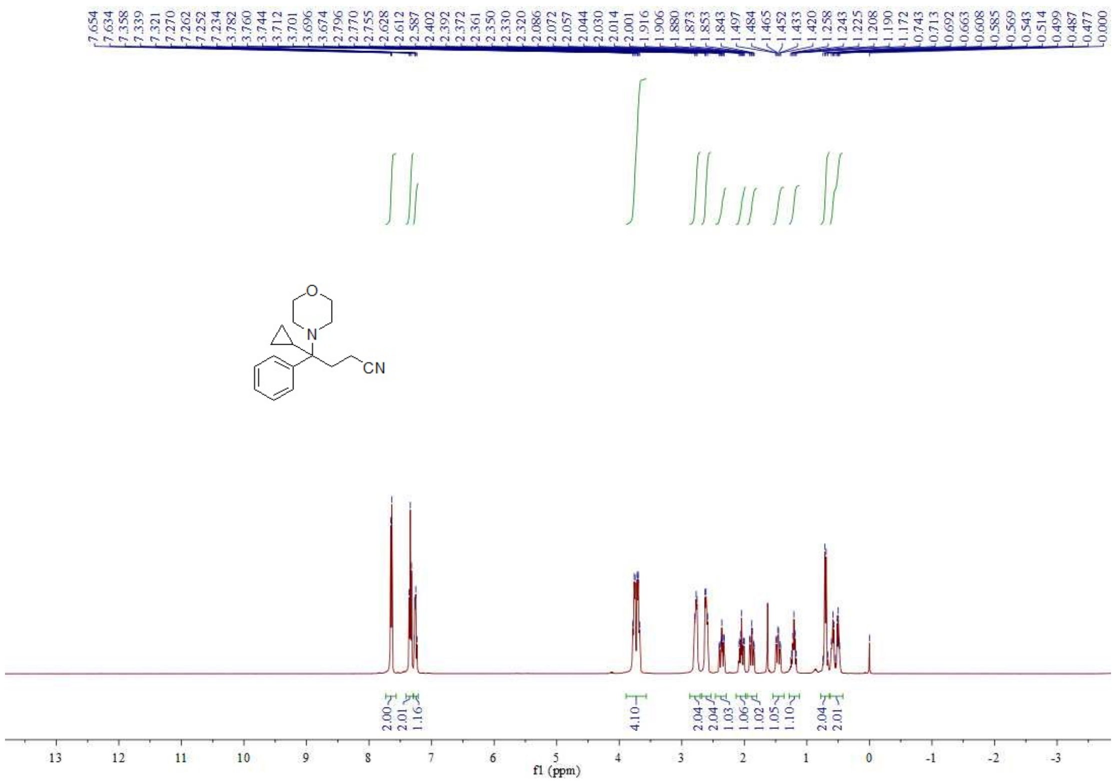

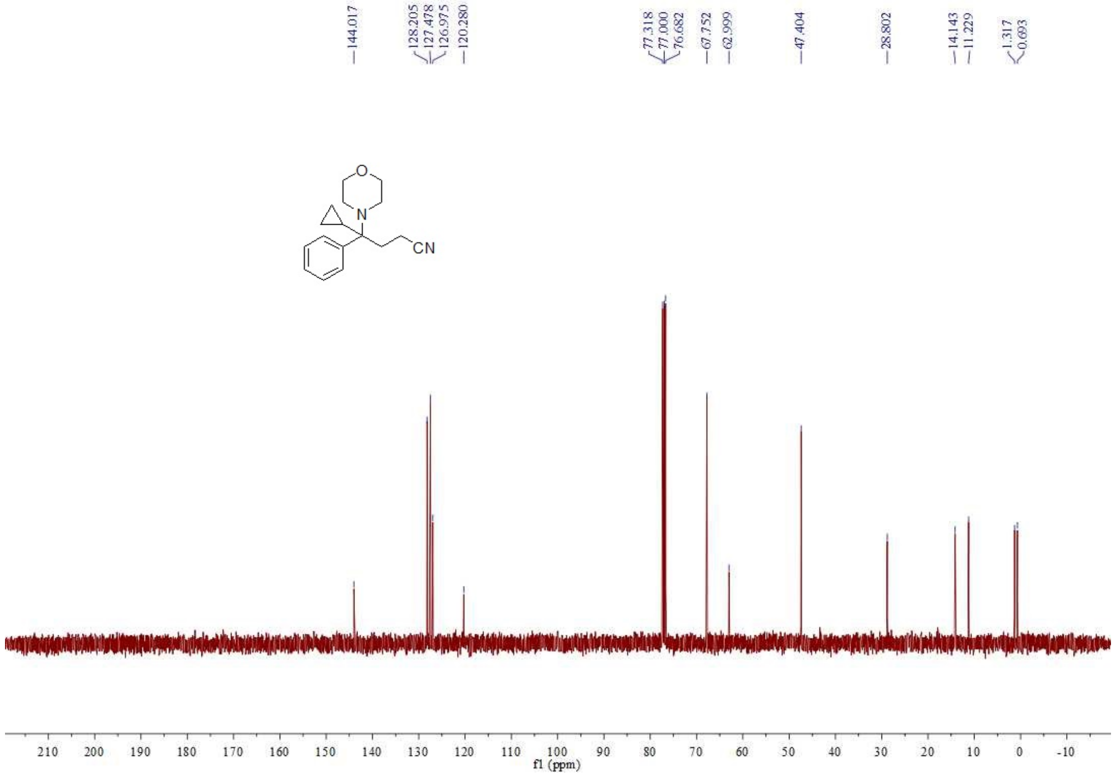

Supplementary Figure 34. ^{1}H, ^{13}C-NMR spectra of product **40**

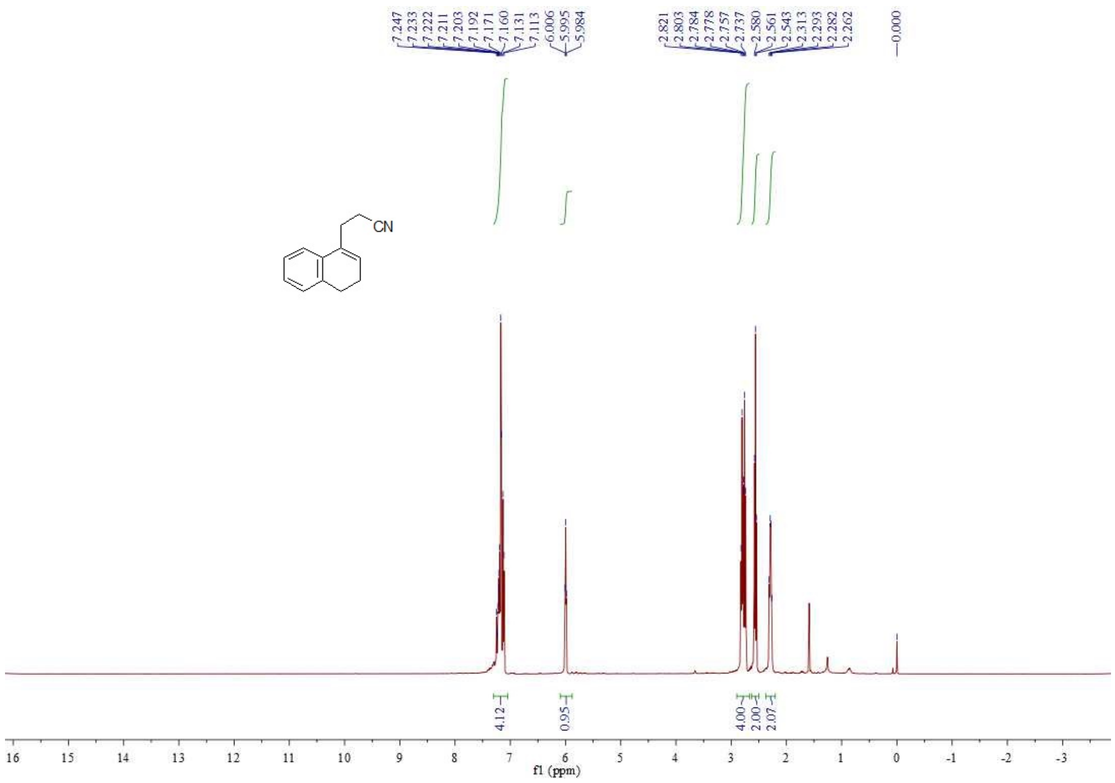

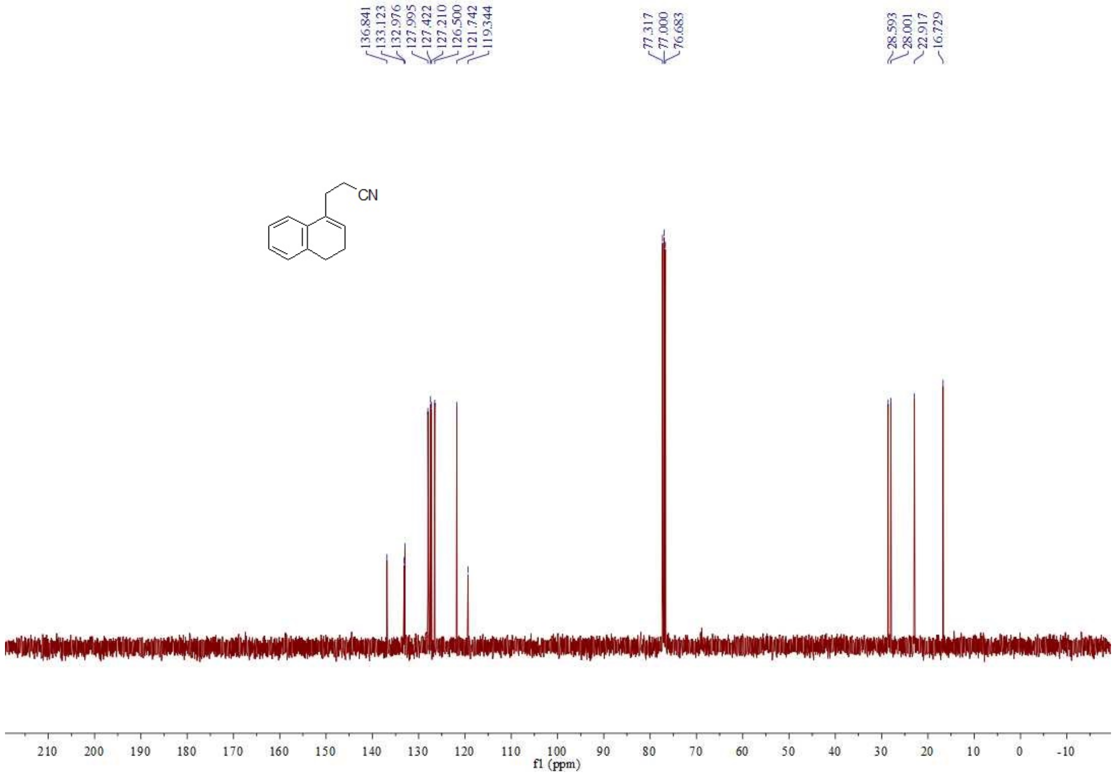

Supplementary Figure 35. ^{1}H, ^{13}C-NMR spectra of product **41**

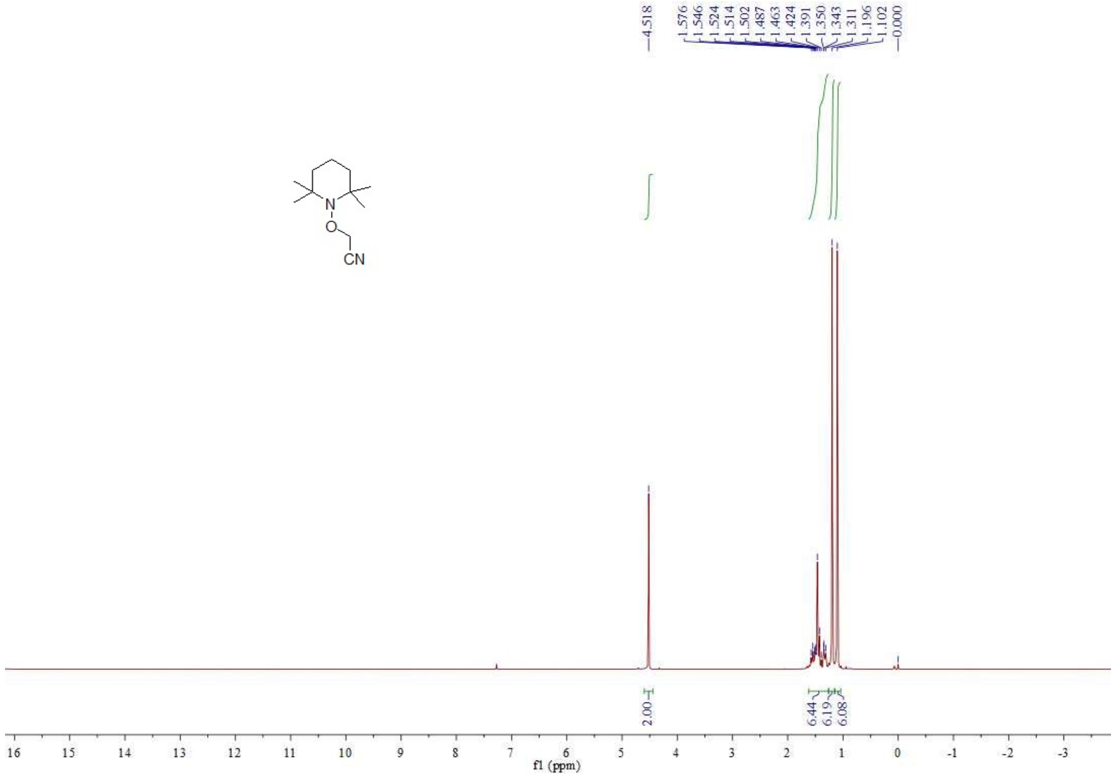

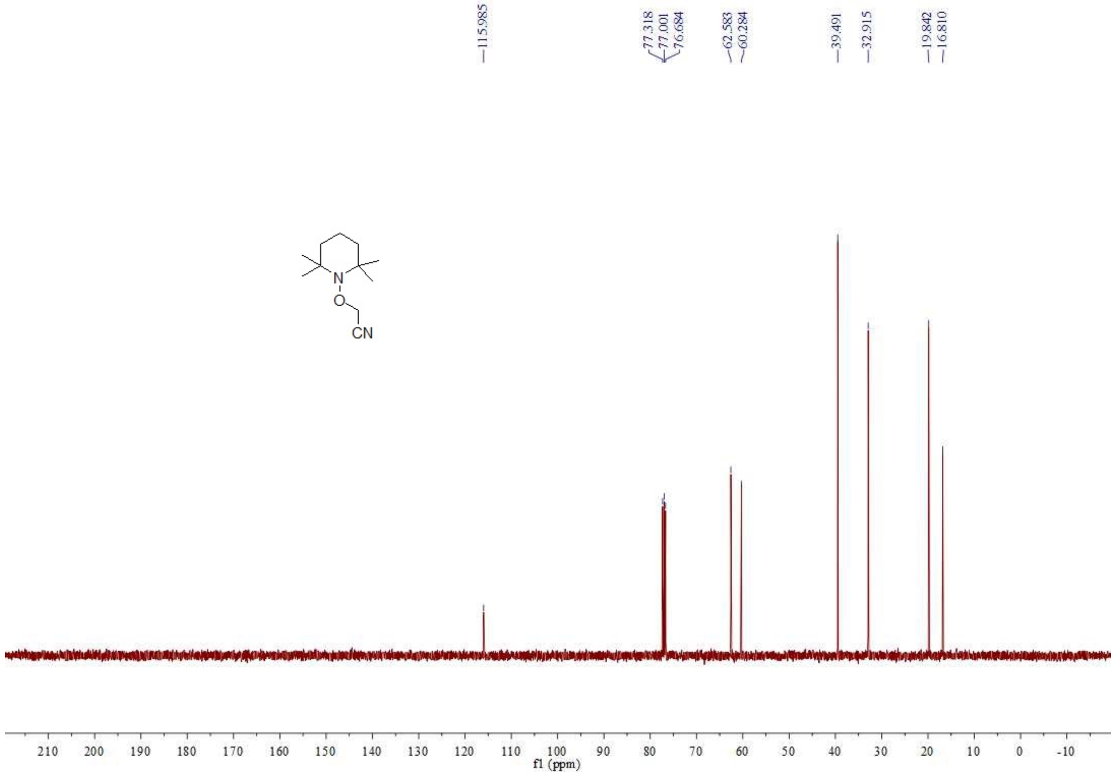

Supplementary Figure 36. ^{1}H, ^{13}C-NMR spectra of product **42**

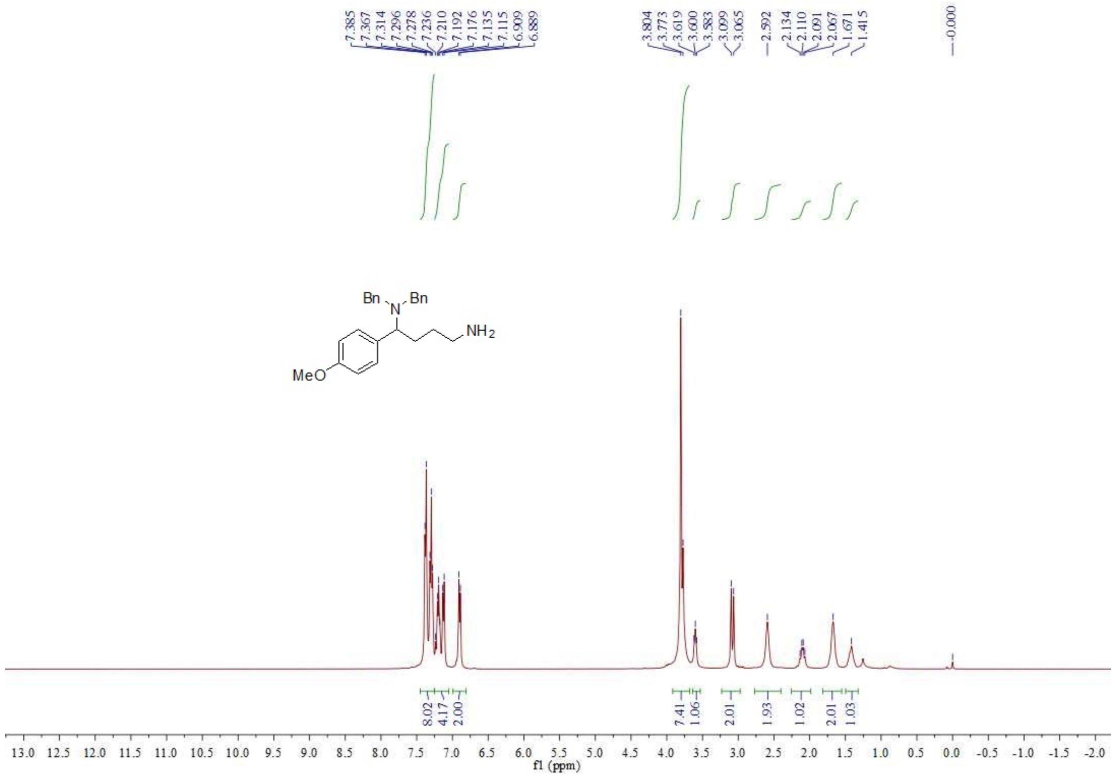

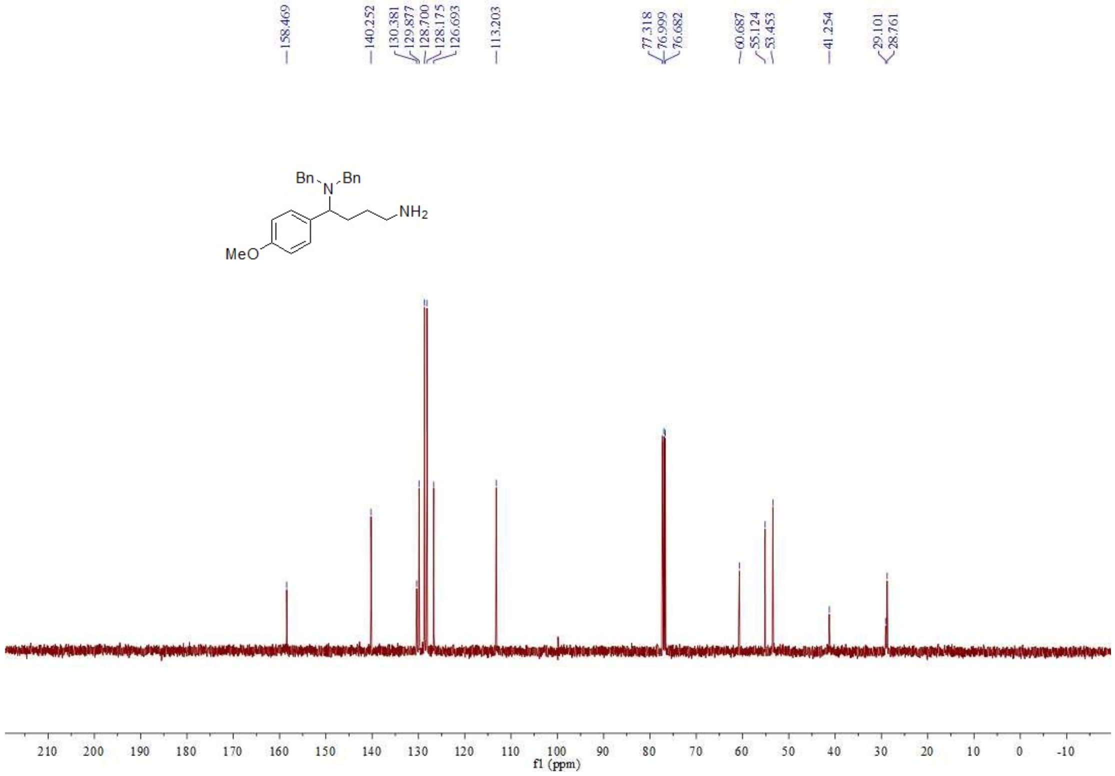

Supplementary Figure 37. ^{1}H, ^{13}C-NMR spectra of product **43**

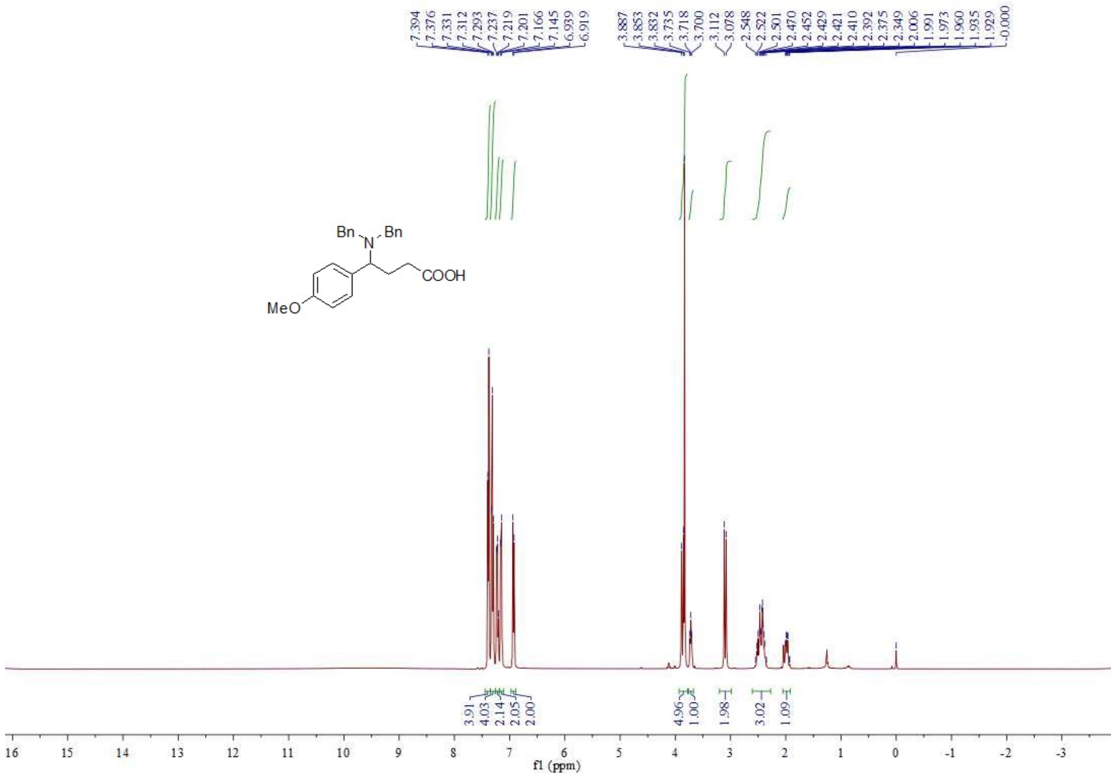

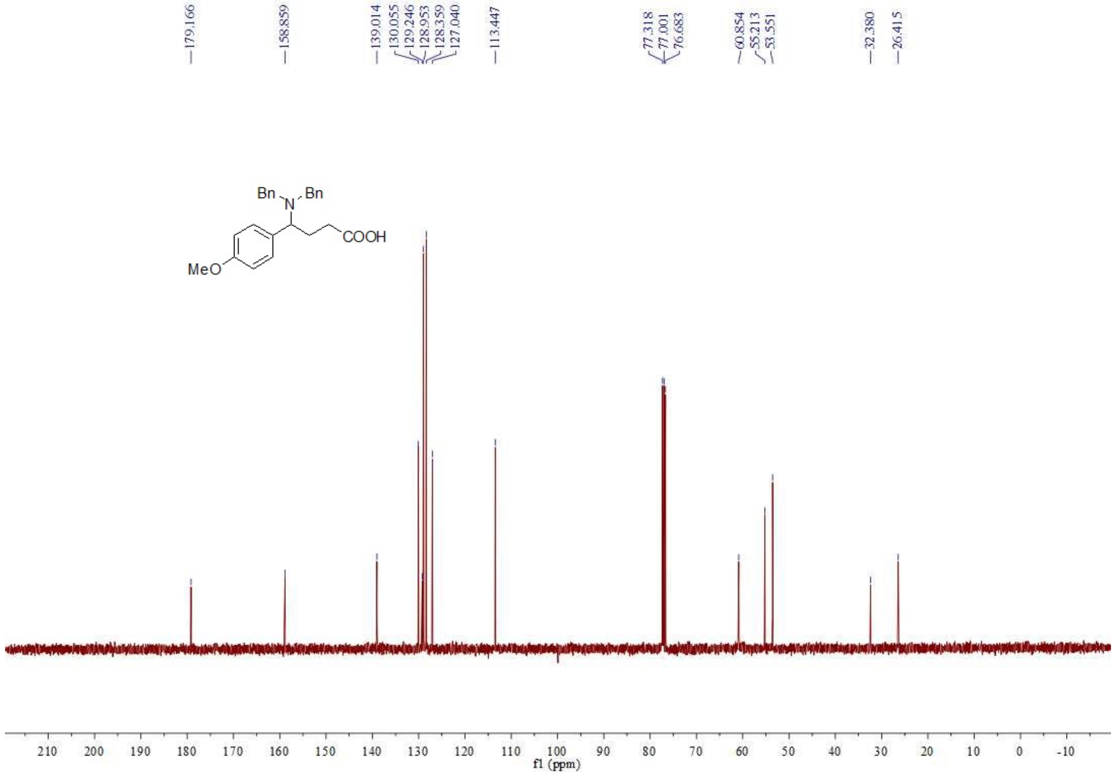

Supplementary Figure 38. ^{1}H, ^{13}C-NMR spectra of product **44**

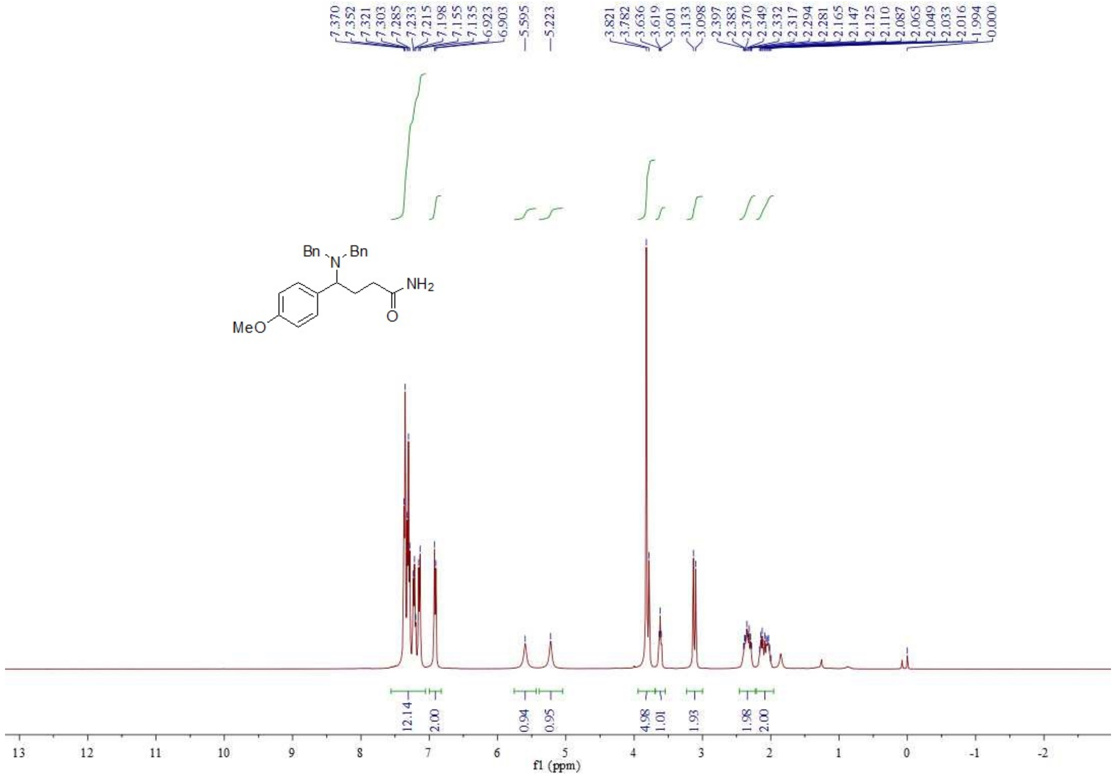

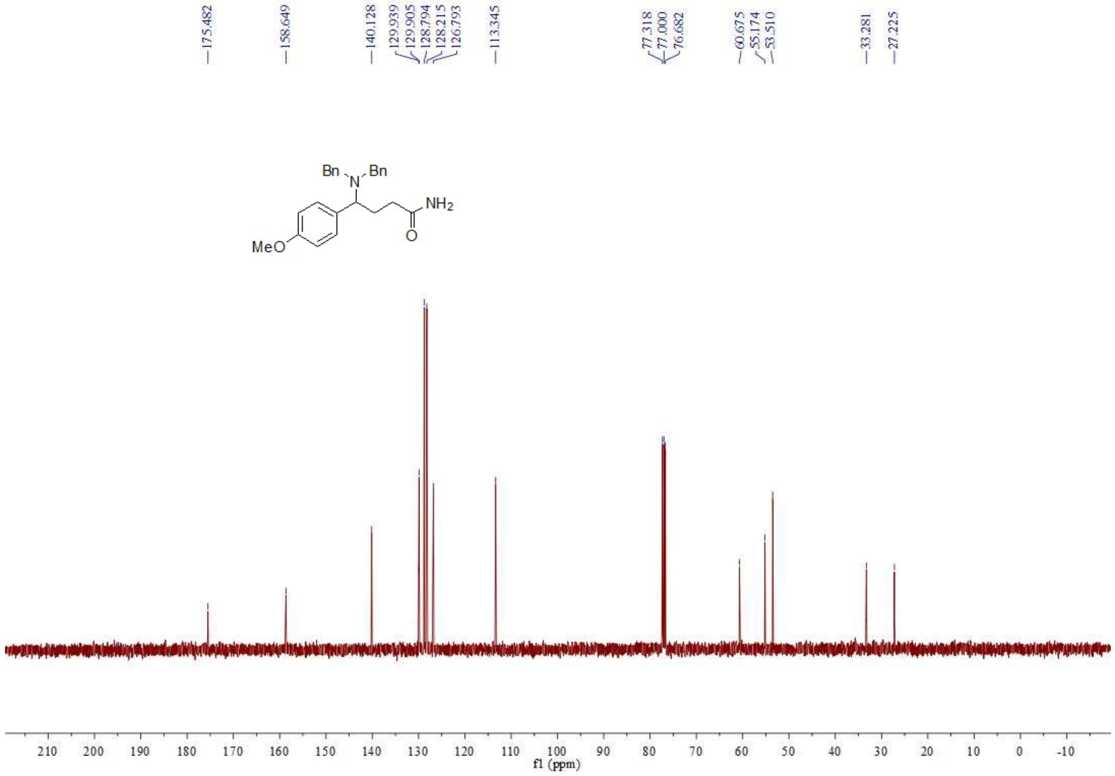

Kinetic Isotopic Effect (KIE) Study: To a Schlenk tube were added $Fe(OTf)_3$ (10 mol%), Ag_2CO_3 (0.6 mmol), **1a** (0.3 mmol), **2a**(1 mL), **2a-3D** (1 mL) and **3a** (0.6 mmol). Then the tube was recharged with argon, and the mixture was stirred at 120 ºC for 2 hours. After cooling to room temperature, the mixture was filtered through a small plugof silica gel to remove the precipitate and washed with with EtOAc (3×10 mL). The mixture was concentrated and subjected to column chromatography to give theproduct. The products were under ^{1}H-NMR analysis. The result was summarized in the equation below.

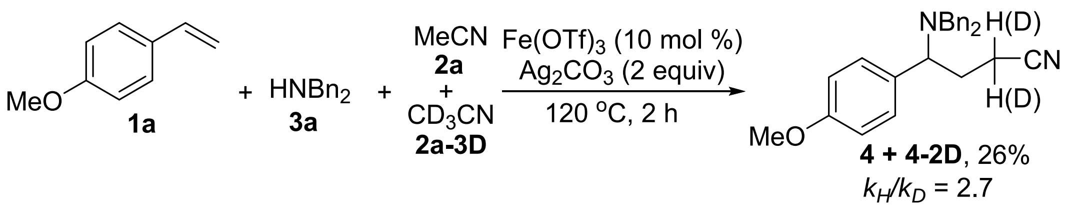

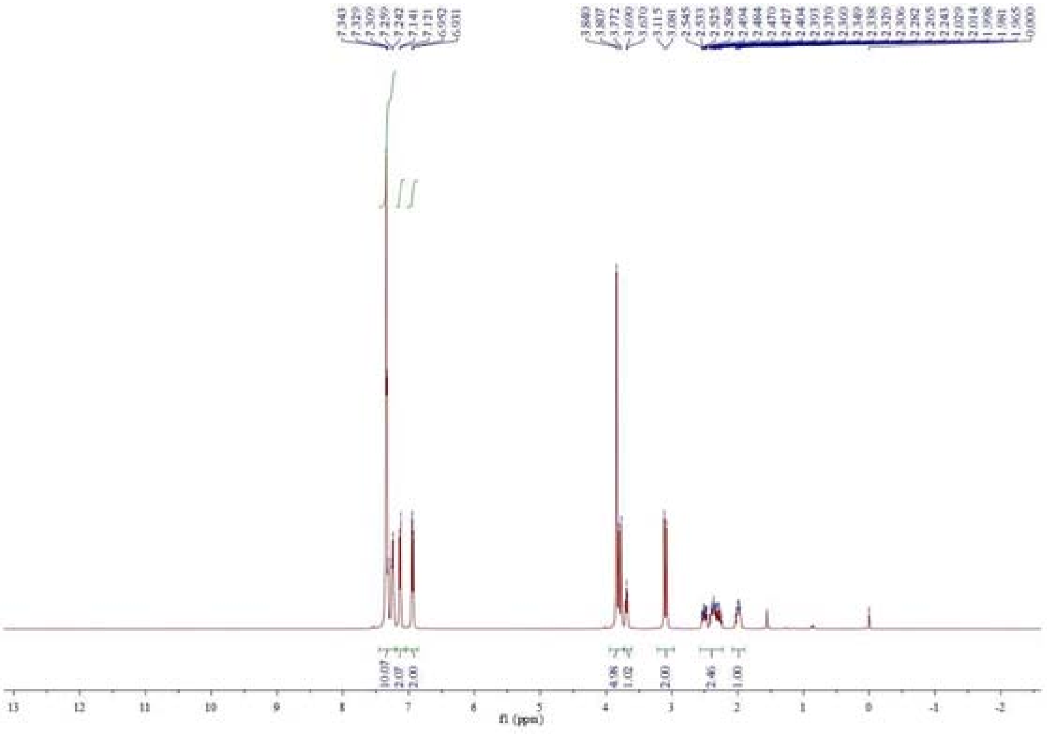

Supplementary Figure 39. ^{1}H-NMR spectrum for the reaction of **2a** and **2a-3D**

Supplementary Figure 40. The X-ray single-crystal diffraction analysis of 4

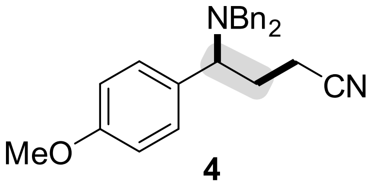

4

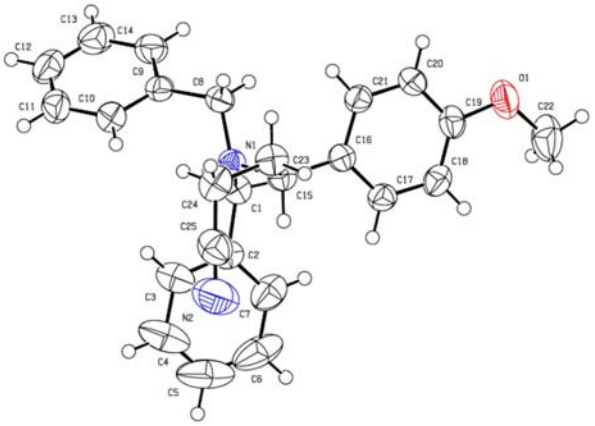

Supplementary Table 1. Crystal data and structure refinement for**4**.

Identification code	**4**
Empirical formula	C25 H26 N2O
Formula weight	370.48
Temperature	296(2) K
Wavelength	0.71073 Å
Crystal system, space group	Monoclinic, P2(1)/n
Unit cell dimensions	a = 8.0485(8) Å alpha = 90 deg.
	b = 10.3008(11) Åbeta = 90.873(2) deg.
	c = 24.936(2) Å gamma = 90 deg.
Volume	2067.1(4) Å^3
Z, Calculated density	4,1.190 Mg/m^3
Absorption coefficient	0.073 mm^-1
F(000)	792
Crystal size	0.23 x 0.20 x 0.20 mm^3
Theta range for data collection	1.63 to 27.42 deg.
Limiting indices	-10<=h<=10,-13<=k<=13,-32<=l<=24
Reflections collected / unique	12245/4672 [R(int) = 0.0321]
Completeness to theta = 27.42	99.0 %
Absorption correction	Semi-empirical from equivalents

Max. and min. transmission 0.9856 and 0.9835

Refinement method Full-matrix least-squares on F^2

Data/restraints/parameters 4672/0/254

Goodness-of-fit on F^2 1.028

Final R indices [I>2sigma(I)] R1 = 0.0511, wR2 = 0.1153

R indices (all data) R1 = 0.0902, wR2 = 0.1345

Largest diff. peak and hole 0.147 and -0.181 e.Å^-3

Supplementary Table 2. Atomic coordinates (x 10^4) and equivalent isotropic displacement parameters (A^2 x 10^3) for ljh041_0m. U(eq) is defined as one third of the trace of the orthogonalized Uij tensor.

	x	y	z	U(eq)
O(1)	12385(2)	2177(1)	51(1)	71(1)
N(1)	6143(2)	4398(1)	1375(1)	38(1)
C(1)	5213(2)	3410(2)	1075(1)	43(1)
N(2)	6696(3)	3317(2)	3079(1)	108(1)
C(2)	4474(2)	2377(2)	1419(1)	44(1)
C(3)	3360(3)	2702(2)	1811(1)	63(1)
C(4)	2576(3)	1763(3)	2104(1)	92(1)
C(5)	2897(4)	475(3)	2010(1)	108(1)

C(6)	4012(4)	134(2)	1628(1)	100(1)
C(7)	4799(3)	1080(2)	1332(1)	67(1)
C(8)	6287(2)	5591(2)	1056(1)	46(1)
C(9)	4622(2)	6256(2)	986(1)	43(1)
C(10)	3593(2)	6460(2)	1416(1)	51(1)
C(11)	2086(2)	7088(2)	1353(1)	59(1)
C(12)	1583(2)	7513(2)	852(1)	65(1)
C(13)	2585(3)	7310(2)	422(1)	67(1)
C(14)	4094(2)	6689(2)	487(1)	56(1)
C(15)	7727(2)	3868(2)	1592(1)	41(1)
C(16)	8961(2)	3394(2)	1180(1)	40(1)
C(17)	9287(2)	2090(2)	1119(1)	45(1)
C(18)	10420(2)	1631(2)	751(1)	49(1)
C(19)	11244(2)	2499(2)	429(1)	48(1)
C(20)	10944(2)	3808(2)	484(1)	49(1)
C(21)	9831(2)	4250(2)	854(1)	45(1)
C(22)	12718(3)	855(2)	-41(1)	86(1)
C(23)	8548(2)	4792(2)	1992(1)	52(1)
C(24)	7444(3)	5209(2)	2450(1)	59(1)
C(25)	7001(3)	4151(2)	2804(1)	67(1)

Supplementary Table 3. Bond lengths [A] and angles [deg] for ljh041_0m.

O(1)-C(19)	1.367(2)
O(1)-C(22)	1.408(2)
N(1)-C(1)	1.4630(19)
N(1)-C(8)	1.469(2)
N(1)-C(15)	1.4816(19)
C(1)-C(2)	1.496(2)
C(1)-H(1A)	0.9700
C(1)-H(1B)	0.9700
N(2)-C(25)	1.129(3)
C(2)-C(3)	1.378(2)
C(2)-C(7)	1.379(2)
C(3)-C(4)	1.372(3)
C(3)-H(3)	0.9300
C(4)-C(5)	1.372(4)
C(4)-H(4)	0.9300
C(5)-C(6)	1.366(4)
C(5)-H(5)	0.9300
C(6)-C(7)	1.382(3)

C(6)-H(6)	0.9300
C(7)-H(7)	0.9300
C(8)-C(9)	1.512(2)
C(8)-H(8A)	0.9700
C(8)-H(8B)	0.9700
C(9)-C(10)	1.380(2)
C(9)-C(14)	1.383(2)
C(10)-C(11)	1.381(2)
C(10)-H(10)	0.9300
C(11)-C(12)	1.379(3)
C(11)-H(11)	0.9300
C(12)-C(13)	1.367(3)
C(12)-H(12)	0.9300
C(13)-C(14)	1.380(3)
C(13)-H(13)	0.9300
C(14)-H(14)	0.9300
C(15)-C(16)	1.521(2)
C(15)-C(23)	1.523(2)
C(15)-H(15)	0.9800
C(16)-C(17)	1.377(2)
C(16)-C(21)	1.395(2)
C(17)-C(18)	1.386(2)

C(17)-H(17)	0.9300
C(18)-C(19)	1.378(2)
C(18)-H(18)	0.9300
C(19)-C(20)	1.377(2)
C(20)-C(21)	1.373(2)
C(20)-H(20)	0.9300
C(21)-H(21)	0.9300
C(22)-H(22A)	0.9600
C(22)-H(22B)	0.9600
C(22)-H(22C)	0.9600
C(23)-C(24)	1.520(2)
C(23)-H(23A)	0.9700
C(23)-H(23B)	0.9700
C(24)-C(25)	1.450(3)
C(24)-H(24A)	0.9700
C(24)-H(24B)	0.9700

C(19)-O(1)-C(22)	118.61(16)
C(1)-N(1)-C(8)	110.40(12)
C(1)-N(1)-C(15)	111.14(12)
C(8)-N(1)-C(15)	115.55(12)
N(1)-C(1)-C(2)	113.95(12)

N(1)-C(1)-H(1A)	108.8
C(2)-C(1)-H(1A)	108.8
N(1)-C(1)-H(1B)	108.8
C(2)-C(1)-H(1B)	108.8
H(1A)-C(1)-H(1B)	107.7
C(3)-C(2)-C(7)	118.25(18)
C(3)-C(2)-C(1)	120.18(16)
C(7)-C(2)-C(1)	121.45(17)
C(4)-C(3)-C(2)	121.0(2)
C(4)-C(3)-H(3)	119.5
C(2)-C(3)-H(3)	119.5
C(3)-C(4)-C(5)	120.2(3)
C(3)-C(4)-H(4)	119.9
C(5)-C(4)-H(4)	119.9
C(6)-C(5)-C(4)	119.7(2)
C(6)-C(5)-H(5)	120.2
C(4)-C(5)-H(5)	120.2
C(5)-C(6)-C(7)	120.1(3)
C(5)-C(6)-H(6)	119.9
C(7)-C(6)-H(6)	119.9
C(2)-C(7)-C(6)	120.7(2)
C(2)-C(7)-H(7)	119.6

C(6)-C(7)-H(7)	119.6
N(1)-C(8)-C(9)	111.36(13)
N(1)-C(8)-H(8A)	109.4
C(9)-C(8)-H(8A)	109.4
N(1)-C(8)-H(8B)	109.4
C(9)-C(8)-H(8B)	109.4
H(8A)-C(8)-H(8B)	108.0
C(10)-C(9)-C(14)	118.00(16)
C(10)-C(9)-C(8)	121.37(15)
C(14)-C(9)-C(8)	120.63(16)
C(9)-C(10)-C(11)	121.30(18)
C(9)-C(10)-H(10)	119.4
C(11)-C(10)-H(10)	119.4
C(12)-C(11)-C(10)	119.80(19)
C(12)-C(11)-H(11)	120.1
C(10)-C(11)-H(11)	120.1
C(13)-C(12)-C(11)	119.54(18)
C(13)-C(12)-H(12)	120.2
C(11)-C(12)-H(12)	120.2
C(12)-C(13)-C(14)	120.49(19)
C(12)-C(13)-H(13)	119.8
C(14)-C(13)-H(13)	119.8

C(13)-C(14)-C(9)	120.88(19)
C(13)-C(14)-H(14)	119.6
C(9)-C(14)-H(14)	119.6
N(1)-C(15)-C(16)	116.07(12)
N(1)-C(15)-C(23)	111.76(13)
C(16)-C(15)-C(23)	111.26(13)
N(1)-C(15)-H(15)	105.6
C(16)-C(15)-H(15)	105.6
C(23)-C(15)-H(15)	105.6
C(17)-C(16)-C(21)	117.07(15)
C(17)-C(16)-C(15)	120.98(14)
C(21)-C(16)-C(15)	121.95(14)
C(16)-C(17)-C(18)	122.23(16)
C(16)-C(17)-H(17)	118.9
C(18)-C(17)-H(17)	118.9
C(19)-C(18)-C(17)	119.43(16)
C(19)-C(18)-H(18)	120.3
C(17)-C(18)-H(18)	120.3
O(1)-C(19)-C(20)	115.17(16)
O(1)-C(19)-C(18)	125.38(16)
C(20)-C(19)-C(18)	119.45(16)
C(21)-C(20)-C(19)	120.49(16)

C(21)-C(20)-H(20)	119.8
C(19)-C(20)-H(20)	119.8
C(20)-C(21)-C(16)	121.32(15)
C(20)-C(21)-H(21)	119.3
C(16)-C(21)-H(21)	119.3
O(1)-C(22)-H(22A)	109.5
O(1)-C(22)-H(22B)	109.5
H(22A)-C(22)-H(22B)	109.5
O(1)-C(22)-H(22C)	109.5
H(22A)-C(22)-H(22C)	109.5
H(22B)-C(22)-H(22C)	109.5
C(24)-C(23)-C(15)	114.62(15)
C(24)-C(23)-H(23A)	108.6
C(15)-C(23)-H(23A)	108.6
C(24)-C(23)-H(23B)	108.6
C(15)-C(23)-H(23B)	108.6
H(23A)-C(23)-H(23B)	107.6
C(25)-C(24)-C(23)	113.36(16)
C(25)-C(24)-H(24A)	108.9
C(23)-C(24)-H(24A)	108.9
C(25)-C(24)-H(24B)	108.9
C(23)-C(24)-H(24B)	108.9

H(24A)-C(24)-H(24B)	107.7
N(2)-C(25)-C(24)	178.3(3)

Symmetry transformations used to generate equivalent atoms:

Supplementary Table 4. Anisotropic displacement parameters (A^2 x 10^3) for ljh041_0m.

The anisotropic displacement factor exponent takes the form:

-2 pi^2 [h^2 a*^2 U11 + ... + 2 h k a* b* U12]

	U11	U22	U33	U23	U13	U12
O(1)	59(1)	70(1)	85(1)	-14(1)	26(1)	5(1)
N(1)	37(1)	38(1)	40(1)	2(1)	0(1)	-2(1)
C(1)	42(1)	47(1)	40(1)	-3(1)	-2(1)	-2(1)
N(2)	125(2)	98(2)	103(2)	13(1)	34(2)	-20(2)
C(2)	43(1)	45(1)	45(1)	2(1)	-11(1)	-8(1)
C(3)	71(1)	66(1)	52(1)	2(1)	6(1)	-19(1)
C(4)	94(2)	120(2)	61(1)	25(1)	-1(1)	-46(2)
C(5)	97(2)	107(2)	118(2)	63(2)	-44(2)	-54(2)
C(6)	88(2)	49(1)	163(3)	26(2)	-55(2)	-22(1)
C(7)	57(1)	47(1)	97(2)	-5(1)	-21(1)	-5(1)
C(8)	42(1)	47(1)	50(1)	8(1)	4(1)	-2(1)

C(9)	41(1)	37(1)	50(1)	5(1)	2(1)	-3(1)
C(10)	51(1)	48(1)	55(1)	1(1)	3(1)	2(1)
C(11)	50(1)	47(1)	81(1)	-10(1)	10(1)	0(1)
C(12)	45(1)	44(1)	104(2)	5(1)	-10(1)	3(1)
C(13)	61(1)	62(1)	76(1)	19(1)	-13(1)	1(1)
C(14)	54(1)	58(1)	56(1)	11(1)	1(1)	-1(1)
C(15)	40(1)	40(1)	42(1)	4(1)	-2(1)	-2(1)
C(16)	34(1)	42(1)	43(1)	0(1)	-3(1)	-2(1)
C(17)	40(1)	40(1)	54(1)	4(1)	-1(1)	-3(1)
C(18)	43(1)	40(1)	64(1)	-5(1)	-6(1)	2(1)
C(19)	34(1)	54(1)	57(1)	-6(1)	2(1)	2(1)
C(20)	38(1)	49(1)	61(1)	7(1)	7(1)	-4(1)
C(21)	38(1)	38(1)	58(1)	2(1)	0(1)	-2(1)
C(22)	70(2)	80(2)	109(2)	-38(1)	17(1)	7(1)
C(23)	52(1)	52(1)	51(1)	-2(1)	-7(1)	-6(1)
C(24)	69(1)	60(1)	49(1)	-9(1)	-8(1)	6(1)
C(25)	69(1)	70(1)	62(1)	-11(1)	9(1)	-7(1)

Supplementary Table 5. Hydrogen coordinates (x 10^4) and isotropic displacement parameters (A^2 x 10^3) for ljh041_0m.

	x	y	z	U(eq)
H(1A)	5950	3005	820	51
H(1B)	4327	3831	872	51
H(3)	3137	3572	1879	75
H(4)	1825	1999	2366	110
H(5)	2357	-162	2207	129
H(6)	4243	-738	1567	121
H(7)	5555	840	1072	81
H(8A)	6725	5380	707	55
H(8B)	7061	6179	1233	55
H(10)	3920	6168	1754	62
H(11)	1414	7225	1648	71
H(12)	568	7934	806	78
H(13)	2247	7591	83	80

H(14)	4766	6561	192	67
H(15)	7422	3099	1799	49
H(17)	8727	1497	1332	54
H(18)	10623	745	722	59
H(20)	11500	4398	268	59
H(21)	9653	5139	887	54
H(22A)	13091	455	287	129
H(22B)	13565	776	-306	129
H(22C)	11724	432	-167	129
H(23A)	9532	4376	2142	62
H(23B)	8907	5561	1802	62
H(24A)	8015	5873	2658	71
H(24B)	6436	5591	2302	71

Supplementary Table 6. Torsion angles [deg] for ljh041_0m.

C(8)-N(1)-C(1)-C(2)	-158.92(14)
C(15)-N(1)-C(1)-C(2)	71.50(17)
N(1)-C(1)-C(2)-C(3)	59.3(2)
N(1)-C(1)-C(2)-C(7)	-124.88(17)
C(7)-C(2)-C(3)-C(4)	-0.8(3)
C(1)-C(2)-C(3)-C(4)	175.09(18)
C(2)-C(3)-C(4)-C(5)	0.2(3)
C(3)-C(4)-C(5)-C(6)	0.6(4)
C(4)-C(5)-C(6)-C(7)	-0.8(4)
C(3)-C(2)-C(7)-C(6)	0.7(3)
C(1)-C(2)-C(7)-C(6)	-175.21(18)
C(5)-C(6)-C(7)-C(2)	0.1(3)
C(1)-N(1)-C(8)-C(9)	68.74(17)
C(15)-N(1)-C(8)-C(9)	-164.08(13)
N(1)-C(8)-C(9)-C(10)	47.7(2)
N(1)-C(8)-C(9)-C(14)	-132.94(16)
C(14)-C(9)-C(10)-C(11)	-0.6(3)
C(8)-C(9)-C(10)-C(11)	178.80(16)

C(9)-C(10)-C(11)-C(12)	0.6(3)
C(10)-C(11)-C(12)-C(13)	-0.2(3)
C(11)-C(12)-C(13)-C(14)	-0.3(3)
C(12)-C(13)-C(14)-C(9)	0.3(3)
C(10)-C(9)-C(14)-C(13)	0.2(3)
C(8)-C(9)-C(14)-C(13)	-179.26(16)
C(1)-N(1)-C(15)-C(16)	61.96(16)
C(8)-N(1)-C(15)-C(16)	-64.84(17)
C(1)-N(1)-C(15)-C(23)	-168.99(13)
C(8)-N(1)-C(15)-C(23)	64.20(17)
N(1)-C(15)-C(16)-C(17)	-109.55(17)
C(23)-C(15)-C(16)-C(17)	121.16(16)
N(1)-C(15)-C(16)-C(21)	71.51(19)
C(23)-C(15)-C(16)-C(21)	-57.77(19)
C(21)-C(16)-C(17)-C(18)	-0.2(2)
C(15)-C(16)-C(17)-C(18)	-179.18(14)
C(16)-C(17)-C(18)-C(19)	-0.7(2)
C(22)-O(1)-C(19)-C(20)	-177.83(17)
C(22)-O(1)-C(19)-C(18)	2.9(3)
C(17)-C(18)-C(19)-O(1)	-179.89(16)
C(17)-C(18)-C(19)-C(20)	0.9(2)
O(1)-C(19)-C(20)-C(21)	-179.51(15)

C(18)-C(19)-C(20)-C(21)	-0.2(3)
C(19)-C(20)-C(21)-C(16)	-0.7(3)
C(17)-C(16)-C(21)-C(20)	0.9(2)
C(15)-C(16)-C(21)-C(20)	179.86(15)
N(1)-C(15)-C(23)-C(24)	54.14(19)
C(16)-C(15)-C(23)-C(24)	-174.32(14)
C(15)-C(23)-C(24)-C(25)	65.6(2)
C(23)-C(24)-C(25)-N(2)	46(8)
